# The genome sequence of the fish pathogen *Aliivibrio salmonicida *strain LFI1238 shows extensive evidence of gene decay

**DOI:** 10.1186/1471-2164-9-616

**Published:** 2008-12-19

**Authors:** Erik Hjerde, Marit Sjo Lorentzen, Matthew TG Holden, Kathy Seeger, Steinar Paulsen, Nathalie Bason, Carol Churcher, David Harris, Halina Norbertczak, Michael A Quail, Suzanne Sanders, Scott Thurston, Julian Parkhill, Nils Peder Willassen, Nicholas R Thomson

**Affiliations:** 1 Department of Molecular Biotechnology, Institute of Medical Biology, Faculty of Medicine, University of Tromsø, N-9037 Tromsø, Norway; 2 The Norwegian Structural Biology Centre, University of Tromsø, N-9037 Tromsø, Norway; 3 The Pathogen Sequencing Unit, The Wellcome Trust Sanger Institute, Wellcome Trust Genome Campus, Hinxton, Cambridge, CB10 1SA, UK

## Abstract

**Background:**

The fish pathogen *Aliivibrio salmonicida *is the causative agent of cold-water vibriosis in marine aquaculture. The Gram-negative bacterium causes tissue degradation, hemolysis and sepsis *in vivo*.

**Results:**

In total, 4 286 protein coding sequences were identified, and the 4.6 Mb genome of *A. salmonicida *has a six partite architecture with two chromosomes and four plasmids. Sequence analysis revealed a highly fragmented genome structure caused by the insertion of an extensive number of insertion sequence (IS) elements. The IS elements can be related to important evolutionary events such as gene acquisition, gene loss and chromosomal rearrangements. New *A. salmonicida *functional capabilities that may have been aquired through horizontal DNA transfer include genes involved in iron-acquisition, and protein secretion and play potential roles in pathogenicity. On the other hand, the degeneration of 370 genes and consequent loss of specific functions suggest that *A. salmonicida *has a reduced metabolic and physiological capacity in comparison to related *Vibrionaceae *species.

**Conclusion:**

Most prominent is the loss of several genes involved in the utilisation of the polysaccharide chitin. In particular, the disruption of three extracellular chitinases responsible for enzymatic breakdown of chitin makes *A. salmonicida *unable to grow on the polymer form of chitin. These, and other losses could restrict the variety of carrier organisms *A. salmonicida *can attach to, and associate with. Gene acquisition and gene loss may be related to the emergence of *A. salmonicida *as a fish pathogen.

## Background

*Aliivibrio salmonicida *(formerly *Vibrio salmonicida*) is a facultative pathogen of fish responsible for causing cold-water vibriosis (CV) in farmed Atlantic salmon (*Salmo salar*), sea farmed rainbow trout (*Oncorhynchus mykiss*) and captive Atlantic cod (*Gadus morhua*) [1]. At the peak of its prevalence in the 1980s infected fish farms suffered heavy losses reaching 50–90% [2]. CV appeared to be effectively controlled in 1998 [3] but before vaccination was introduced, *A. salmonicida *was estimated to have been responsible for over 80% of disease related losses to the Norwegian aquaculture industry [4]. Although the impact of *A. salmonicida *on the aquaculture industry is primarily on salmonoids there is concern it poses a risk to new commercially important species for which farming is at an early stage or is planned. The decline in the wild Atlantic cod population has lead to a massive expansion of cod aquaculture. In Norway alone 7410 tons of farmed cod were sold in 2005, which is more than twice the amount from previous year [5]. So far the cod farming industry has only suffered a few outbreaks of CV, and only in unvaccinated fish. However, despite this successful treatment the CV vaccine is administered by intraperitoneal injection and its use is associated with severe side-effects such as impaired growth, intra-abdominal lesions [6] and adhesions in the abdominal cavity of the fish that may affect physiological functions and reduce the quality of the final product [7]. Hence, alternative approaches and vaccines are essential.

The halophilic and psychrophilic bacterium belongs to *Vibrionaceae*, which includes 85 species found in a wide range of aquatic environments in free-living forms and attached to both biotic and abiotic surfaces. Plankton organisms, mainly copepods, host large populations of bacteria. The attachment to zooplankton may enhance environmental survival of *Vibrionaceae *which are able to break down the chitinaceous exoskeleton and utilize the polysaccharides as an abundant source of carbon and nitrogen [8]. *Vibrionaceae *are also found associated with, and are pathogens of, other aquatic organisms such as fish, mussels, corals, molluscs, seagrass, shrimps and squid [9]. Currently the genome sequences of nine *Vibrionaceae *have been published. We report here the complete genome sequence of the first fish pathogenic *Vibrionaceae*.

During an infection *A. salmonicida *elicits tissue degradation, hemolysis and sepsis. Clinical symptoms such as severe anaemia and extensive haemorrhages on the surface of all internal organs of the fish are commonly observed. However, very little is known about the molecular mechanisms that produce the pathology of these infections and the genome should provide an insight into evolution and mechanisms involved in mediating the disease. The cod isolate *A. salmonicida *strain LFI1238 taken from the head kidney (lymphoid organ) of a diseased fish was chosen for sequencing in order to better understand pathogen-host interactions.

## Results and discussion

### I. General features of the genome

**Figure 1:**
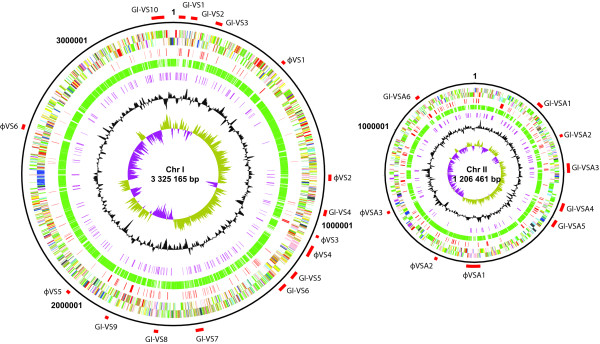
**Schematic circular diagrams of chromosomes I and II of *A. salmonicida *LFI1238, where appropriate categories are shown as pairs of concentric circles representing both coding strands**. Key to the chromosomal circular diagrams (outside to inside): scale (in Mb), annotated CDS, unique CDSs compared to the other *Vibrionaceae *species (red), orthologues shared with the other *Vibrionaceae *species (green), IS element transposases (purple), % G+C content, G+C deviation (>0% olive, <0% purple). Colour coding for CDSs (according to predicted function): dark blue, pathogenicity/adaptation; black, energy metabolism; red, information transfer; dark green, surface associated; cyan, degradation of large molecules; magenta, degradation of small molecules; yellow, central/intermediary metabolism; pale green, unknown; pale blue, regulators; orange, conserved hypothetical; brown, pseudogenes; pink, phage + IS elements; grey, miscellaneous. The positions of phage elements and GIs larger than 5 kb are marked (red) and labelled in accordance with Table 2.

The general features of the *A. salmonicida *strain LFI1238 (LFI1238) genome are summarized in Table 1. The genomic G+C content of 39.6% is relatively low in comparison to other sequenced *Vibrionaceae*. Characteristically for members of *Vibrionaceae *[10] the *A. salmonicida *genome consists of two circular chromosomes of 3.3 and 1.2 Mb (chr I and chr II respectively) (Figure 1). The presence of essential genes on chr II indicates that this replicon is not a dispensable megaplasmid [11]. However unlike the other *Vibrionaceae *sequenced LFI1238 also carries four circular plasmids designated pVSAL840 (83.5 kb), pVSAL320 (30.8 kb), pVSAL54 (5.4 kb) and pVSAL43 (4.3 kb) which represent 2.7% of the total genomic DNA and harbour 111 protein coding sequences (CDSs; Table 1 and [Additional file 1A]).

**Table 1:** General overview of the *A. salmonicida *genome.

	**Chr I**	**Chr II**	**pVSAL840**	**pVSAL320**	**pVSAL54**	**pVSAL43**	**Total**
**Number of bases**	3 325 165	1 206 461	83 540	30 807	5 360	4 327	4 655 660
**GC percentage**	39.83	39.06	40.07	37.28	38.1	35.61	
							
**Number of CDS**	3 070	1 105	72	33	3	3	4 286
**Coding percentage**	86.8	87.5	85.3	77	65.8	62.3	
**Average CAI**	0.59	0.56	0.48	0.53	0.49	0.52	
							
**tRNA**	94	13	0	0	0	0	107
**rRNA operons**	11	1	0	0	0	0	12
**Misc RNA**	18	2	0	0	0	0	20
							
**Pseudo-/partial genes**	245	119	2	4	0	0	370
**Transposases**	350	164	2	4	0	1	521
**IS elements**	188	93	2	4	0	1	288

**Figure 2:**
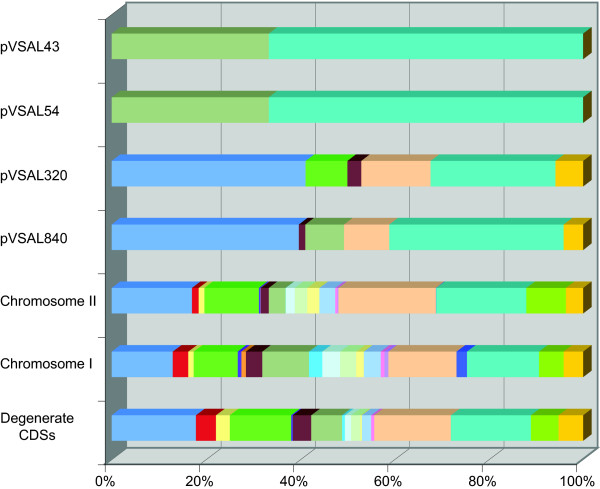
**Representation of the functional distribution of CDSs encoded by the six replicons of *A. salmonicida *strain LFI1238**. The bottom bar summarizes the functional distribution of degenerated genes from all six replicons. Colour coding for the CDSs based on a modified hierarchical protein class coding scheme of Monica Riley's [78] are: light blue, unknown function; red, information transfer; yellow, protection responses; green, transport and binding proteins; violet, adaptation; orange, cell division; plum, macromolecule metabolism; grey, macromolecule synthesis and modification; sky blue, amino acid biosynthesis; light turquoise, biosynthesis of cofactors; light green, central intermediary metabolism; light yellow, degradation of small molecules; pale blue, energy metabolism – carbon; pink, fatty acid biosynthesis; purple, nucleotide biosynthesis; light orange, cell envelope; blue, ribosome constituents; aqua, foreign DNA; lime, regulation; gold, miscellaneous.

The functional distribution of CDSs between the chromosomes is similar to that reported for other *Vibrionaceae *[12]: chr I carries the majority of CDSs needed for DNA replication, cell division, biosynthesis of amino acids and nucleotides. Conversely, the majority of CDSs involved in adapting to environmental changes, such as stress response functions, proteins associated with the cell envelope and proteins that could not be assigned any function are encoded on chr II (Figure 2). From similarity searches comparing all of the LFI1238 CDSs against the CDSs from the other published *Vibrionaceae *genomes, it is apparent that *A. salmonicida *shares more orthologous genes with *Aliivibrio fischeri *(70%) than the other *Vibrionaceae *compared (average 55–60% shared orthologs). These observations are consistent with 16S rRNA gene sequence analysis data [13] and support the reclassification of these two species, together with *Aliivibrio wodanis *and *Aliivibrio logei *as a separate genus [14].

The presence of multiple plasmids is characteristic of *A. salmonicida *[15] with many which are common to isolates from diverse geographical areas in the North Atlantic Ocean (Norway, Canada, the Shetland Islands, Faroe Islands). From plasmid profiles plasmids of the same size as pVSAL43, pVSAL54 and pVSAL320 are common to isolates from all of the above regions. However, LFI1238 pVSAL840 appears to be restricted to isolates from the northern parts of Norway where it is found in strains alongside either pVSAL43, pVSAL54 and pVSAL320, or together with pVSAL320 [16]. pVSAL840 harbours a *tra *locus containing 21 CDSs with functions related to plasmid conjugation. This region is highly syntenic with the *tra *locus of the conjugation plasmid pYJ016 identified in *Vibrio vulnificus *[17] and plasmid pES100 in *A. fischeri *[18], and suggests a similar function involved in conjugation for pVSAL840.

Plasmids pVSAL43 and pVSAL54 are predicted to encode acyltransferases. Acyltransferases have the potential to change the acetylation state of the lipopolysaccharide (LPS) and so maybe important in providing antigenic variability of the cell surface to give better protection against the host antibody immune recognition [19]. In a recent study, the expression of an iron ABC transporter harboured on pVSAL320 was shown to be dependent upon iron and probably regulated by the ferric uptake regulator Fur [20]. pVSAL320 may therefore be important for the non-siderophore based uptake of ferrous iron. Valla and colleagues (1992) showed that a plasmid cured strain of *A. salmonicida *when injected through the intraperetoneal route was still able to cause CV in salmon [21]. Therefore although these plasmids may contribute to colonisation and virulence they are not essential, at least by this route of infection.

The most striking feature of the *A. salmonicida *genome is the high number of insertion sequence (IS) elements relative to other *Vibrionaceae*. In total *A. salmonicida *carry 521 CDSs (12.2% of all CDSs) representing 288 whole and partial IS elements (Table 1), compared to only one IS element in *A. fischeri*. These IS elements can be subdivided into 20 different types (denoted VSa1 – VSa20), and fall into 12 different IS families based on sequence similarities with defined families in the IS Finder database [22] [see Additional file 2]. The relative proportion of transposases is slightly larger in chr II than in chr I (14.8 and 11.4%, respectively). IS element insertions have disrupted 183 CDSs (4.3% of the total CDSs) in the chromosomes and plasmids. Most of these "natural knock-outs" are probably not translated to give functional products. The distribution of the IS elements suggests that the IS elements present in high numbers on the chromosomes have spread to the plasmids by transposition. However, VSa3 and VSa4 are found exclusively on pVSAL840, VSa19 is restricted to pVSAL320 and pVSAL54 carries none of the *A. salmonicida *IS elements. This suggests that these plasmids do not tolerate insertions or that they are relatively recent acquisitions and that pVSAL54 is the most recently acquired. However, Codon Adaptation Index (CAI) analysis, which measures the relative adaptiveness of the codon usage of genes towards the codon usage of highly expressed genes [23], revealed that genes on pVSAL840 (0.48) deviate more from the average genome composition (0.58) than the other plasmids (Table 1). In addition the Codon Bias Index (CBI) versus CAI plot described in [24] for *A. salmonicida *clearly showed that genes on pVSAL840 deviate most from the genome background, suggesting that this is likely to be the most recent acquired plasmid [see Additional file 3].

### II. Genome structure

Compositional asymmetries (GC deviation) in the leading and lagging strand of DNA, with bias towards G on the leading strand of the bidirectional replication fork, is a common characteristic of bacterial genomes [25]. It is evident from Figure 1 that both of the *A. salmonicida *chromosomes show anomalies in their GC deviation. Significantly IS elements are found flanking all large regions showing an aberant GC deviation. Since their homologous DNA can serve as recombinational cross-over points they are likely to be largely responsible for the apparent anomalies [26]. Consistent with this, whole genome comparison with *A. fischeri *also shows that these anomalous regions represent breaks in synteny [see Additional file 4].

By designing PCR primers to amplify across the borders of these anomalous regions we discovered that several genomic configurations may exist within a population of any given isolate (data not shown). It has been suggested that this type of interreplichore recombinations have an effect on the gene dosage, whereby the continual initiation of replication folks leads to genes closer to the origin being at a higher relative gene dosage than those at the terminus. It has also been shown that gene orientation is under selection, with essential genes being preferentially encoded on the leading strand; this is hypothesised to be due to avoidence of the deleterious effects of collisions between the transcription and translation machinery [27]. How stable any given genomic configuration is and what affect this has on transcription in *A. salmonicida *is yet to be determined, but similar rapid rearrangements have been reported in other genomes with high IS element loads [28].

Interestingly in addition to mediating homologous recombination, IS elements also border three regions in the chromosome that are found duplicated in the plasmids [see Additional file 1B]. Two such regions, each encoding three CDSs from pVSAL840 and pVSAL320 respectively, are the flanking parts of the genomic island GI-VS1 (Table 2). The duplicated CDSs displayed nucleotide sequence identity up to 100%, and the functions of most are unknown. The third region carries four CDSs of which two encode a hemolysin co-regulated protein (Hcp) and a VgrG protein. Both Hcp and VgrG are virulence effector proteins secreted by the Type VI secretion system. Codon usage analysis clearly showed that the duplicated genes cluster more closely to the plasmid genes than to the genome background [see Additional file 3], which suggests that the genes originated from the plasmids. Thus this recombination between IS elements represents a mechanism by which to introduce new functions into the chromosome from a highly variable complement of plasmids.

**Table 2:** Regions larger than 5 kb showing some of the characteristics^1 ^of bacteriophages (ϕ) and GIs.

**Type**	**Size (kb)**	**GC content**	**CDSs**	**Description relevant content**
**Chr I**				
				
ϕ VS1	8.8	33.9	VSAL_I0388-I0398	Putative remnant harbouring shufflon-specific DNA recombinase
ϕ VS2	15.2	40.5	VSAL_I0764-I0783	Compound transposon harbouring phage remnants
ϕ VS3	6.8	34.3	VSAL_I0966-I0969	Putative phage remnant harbouring a phage integrase
ϕ VS4	34.1	40.8	VSAL_I1008-I1051	Complete K139-like phage. Flanked by one IS element
ϕ VS5	10.7	31.5	VSAL_I1921-I1930	Phage remnant harbouring putative exported proteins. Flanked by one IS element
ϕ VS6	12.2	36.2	VSAL_I2480-I2489	Putative phage remnant harbouring a phage integrase. Flanked by one IS element
GI-VS1	18.9	36.7	VSAL_I0019-I0035	Compound transposon harbouring duplicated plasmid genes
GI-VS2	13.7	38.3	VSAL_I0056-I0070	Compound transposon encoding chromosome partitioning proteins
GI-VS3	18.8	39.8	VSAL_I0129-I0144	Compound transposon encoding proteins involved in siderophore synthesis and uptake
GI-VS4	13.9	40.6	VSAL_I0884-I0872	Putative GI, encoding exported proteins, a peptidase and a nuclease
GI-VS5	24.2	38.2	VSAL_I1105-I1125	Putative GI encoding a putative protein secretion system (T6SSI). Flanked by one IS element
GI-VS6	23.5	36.8	VSAL_I1166-I1185	Compound transposon encoding a putative protein secretion system (T6SSII)
GI-VS7	19.7	35.9	VSAL_I1463-I1492	Compound transposon encoding a antibiotic resistance protein and a cold-shock protein
GI-VS8	10.5	33.3	VSAL_I1620-I1633	Compound transposon encoding various proteins with unknown function
GI-VS9	10.9	39.9	VSAL_I1787-I1794	Putative GI, encoding a restriction enzyme and a DNA methylase
GI-VS10	38.2	36.2	VSAL_I3009-I3042	Compound transposon encoding proteins involved in LPS and CPS biosynthesis
**Chr II**				
				
ϕ VSA1	26.4	36.4	VSAL_II0534-II0566	Putative phage remnant harbouring a toxin and an antitoxin
ϕ VSA2	6.5	34.6	VSAL_II0629-II0638	Putative phage remnant harbouring a phage integrase
ϕ VSA3	5.1	35.7	VSAL_II0739-II0745	Putative remnant harbouring genes for phage replication
GI-VSA1	13.7	39.1	VSAL_II0117-II0128	Compound transposon encoding an ABC transporter. Flanked by one IS element
GI-VSA2	6.4	34.6	VSAL_II0202-II0208	Compound transposon encoding an acyltransferase and hypothetical proteins
GI-VSA3	16.2	38.2	VSAL_II0257-II0272	Compound transposon encoding exported and membrane proteins
GI-VSA4	15.8	37.9	VSAL_II0321-II0335	Putative GI encoding a glycosyl transferase and membrane proteins. Flanked by one IS element
GI-VSA5	15.4	35.3	VSAL_II0362-II0380	Putative GI encoding a protein secretion system (Flp-type pilus)
GI-VSA6	8.9	37.9	VSAL_II0986-II0990	Putative GI encoding hypothetical proteins. Flanked by IS element

The genome position, sizes, number of CDSs and G+C content of the phages and GIs are listed, together with a description of the most important content^2^.

^1^Abnormal G+C content; GC deviation; repeats flanking the ends; insertion next tRNAs; and the presence of phage genes.

^2^A complete list of the CDSs are provided in [Additional file 10].

#### 1. Gene acquisition

In addition to the plasmids and the IS elements, the genome of *A. salmonicida *carries other mobile genetic elements, including nine prophages as well as 16 regions which have the characteristics of genomic islands (Table 2) [29]. The tailed phage ϕ VS4 present on chr I has an overall GC content of 40.8%, slightly higher than the chromosome average (39.8%). The majority of the 43 CDSs show considerable homology and synteny to the K139 phage of *Vibrio cholerae *strain O139 [30] [see Additional file 5], but this phage is not found in any of the other sequenced *Vibrionaceae *genomes. ϕ VS4 is likely to be the only complete prophage within the *A. salmonicida *genome (Table 2). The remaining 8 prophage-like regions are likely to be remnants.

In total, 25 regions larger than 5 kb were identified as having atypical DNA compositional and being present in *A. salmonicida *but absent the other *Vibrionaceae *genomes (Table 2). Although the majority of CDS encoded on these regions of difference are of no known function, some encode proteins involved in secretion, biosynthesis of capsular polysaccharides (CPS) and biosynthesis and uptake of siderophores [see Additional file 6].

#### 2. Duplications

In addition to the regions duplicated on the plasmids and chromosomes Chr I also carries an additional perfect duplication of approximately 29 kb. PCR analysis of 27 different *A. salmonicida *isolates confirmed the duplication at the same locations in all tested isolates [see Additional file 7]. Each duplicate contains 27 genes, the majority encoding products involved in the biosynthesis of constituents of the LPS. L-rhamnose is present in the O-antigens of Gram-negative bacteria [31]. Four genes, *rmlBADC*, necessary for the conversion of D-glucose 1-phosphate to dTDP-L-rhamnose are present in the repeat. Seven genes are similar to those found in the *wav *gene cluster of *V. cholerae*. The *wav *genes are responsible for the synthesis of LPS core oligosaccharides [32]. Nesper and colleges suggested that genetic exchange of *wav *genes could improve outer membrane stability by altering the structure of the core LPS. In such case, it would provide for better adaptation to different niches. However, the duplicates in LFI1238 are identical at the nucleotide level and homologous recombination would therefore not increase the variety of the surface molecules expressed in the bacteria. On the other hand, in *Haemophilus influenzae *genes involved in the capsule expression are located within an 18 kb *cap *locus. Up to five copies of the locus have been detected, and a relationship between the number of copies of the *cap *locus and the production of capsule has been demonstrated [33]. We speculate that the amplification may increase the gene expression and lead to increased LPS production. Espelid *et al*. observed that ball-shaped aggregates containing the protein/lipopolysaccharide VS-P1, the dominant immunoreactive antigen of *A. salmonicida*, were released in large quantities from the bacterial membrane inside the host [34]. It has been suggested that much of the specific immune response of the fish may be directed against this "smoke screen" [35]. Increased LPS production by *A. salmonicida *could therefore be advantageous when entering a host.

#### 3. Gene loss

In total we identified 185 pseudogenes (4.3% of the total CDSs) containing frameshift and nonsense mutations that might disrupt expression of functional products [see Additional file 3]. Loss of functions seems to occur across all functional classes of products, but a striking number of transposases, transport proteins and proteins associated with the cell envelope are included in this list (Figure 2 and [Additional file 3]).

The accumulation of pseudogenes is high for genes involved in the utilisation of the polysaccharide chitin. Chitin (GlcNAc)_n _is an insoluble homopolymer of N-acetyl-D-glucosamine (GlcNAc), and is highly abundant in marine environments as constituents of the exoskeleton of crustaceans and zooplankton. Chitin is important for the attachment of bacteria to a carrier organism such as copepods [36,37] and known to be an important as a nutrient source [38]. Furthermore in a recent study Hunt *et al*. showed that the majority of genes involved in chitin degradation are conserved among the *Vibrionaceae *[39].

**Figure 3:**
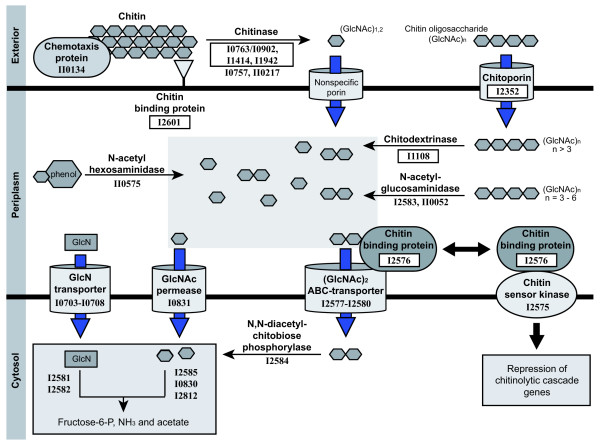
**Key steps in the chitinolytic cascade**. Functional assignment of *A. salmonicida *CDSs are derived from bioinformatical analysis, and the abbreviated gene IDs are indicated at each step. Putative non-functional *A. salmonicida *products are boxed. Initially, a methyl chemotaxis protein (VSAL_II0134) and a chitin binding protein (VSAL_I2601) are involved in sensing and attachment to chitin respectively. Extracellular chitinases (VSAL_I0763/I0902, VSAL_I1414, VSAL_I1942, VSAL_I0757 and VSAL_II0217) partly break down chitin. Chitin oligomers (GlcNAc)_n _are translocated across the outer membrane by chitoporin (VSAL_I2352), while the transport of monomers and dimers (GlcNAc)_1,2 _is mediated by unspecific porins. In the periplasm, the chitin oligomers are further degraded to yield (GlcNAc)_1,2 _by chitodextrinase (VSAL_I1108), N-acetylglucosamidase (VSAL_I2583 and VSAL_II0052) and N-acetylhexosamidase (VSAL_II0575). (GlcNAc)_1 _are transported across the inner membrane by a permease (VSAL_I0831), while the transport of (GlcNAc)_2 _are mediated by an ABC-transporter (VSAL_I2577- I2580). Deacetylated monomers (GlcN) are transported into the cytosol by a PTS transporter (VSAL_I0703- I0708). Cytoplasmic enzymes (VSAL_I2581- I2582, VSAL_I2584- I2585, VSAL_I0830 and VSAL_I2812) convert the transport products into fructose-6-P, acetate and ammonia. In the absence of chitin, the perisplasmic chitin oligosaccharide binding protein CBP (VSAL_I2576) binds to the chitin sensor/kinase ChiS (VSAL_I2575) and represses transcription of chitinolytic genes. In presence of chitin, the complex dissociates as CBP binds to the chitin polysaccharides and chitinolytic genes are expressed.

In *A. salmonicida *seven of the pseudogenes represent key components in the chitinolytic cascade (Figure 3 and [Additional file 8]) including a methyl accepting chemotaxis gene (VSAL_I2601) that may be involved in motility toward chitin [40], three chitinases (VSAL_I1942, VSAL_I0902/I0763 and VSAL_I1414) involved in the extracellular breakdown of chitin to chitin oligosaccharides [41,42], a chitoporin (VSAL_I2352) responsible for mediating transport of chitin oligosaccharides into the periplasma [43], and a chitodextrinase (VSAL_I1108) involved in the periplasmic breakdown of chitin oligosaccharides [44].

In addition, several genes involved in the chitinolytic cascade are regulated by chitin oligosaccharides and a two-component chitin catabolic sensor/kinase encoded by *chiS *[40,45]. The gene regulation on the transcriptional level is not known, but the periplasmic chitin binding protein (CBP) is required for ChiS-regulation (Figure 3). The CBP orthologue in *A. salmonicida *(VSAL_I2576) contains a frameshift. The functional loss of genes thought to be regulated by ChiS/CBP is likely to have preceded, and perhaps facilitated, the degeneration of this gene.

To investigate whether the loss of these genes has impaired the ability of *A. salmonicida *to utilize chitin, six *A. salmonicida *isolates including LFI1238 were grown on a minimal media containing either α-chitin (GlcNAc)_n _or GlcNAc as the only source of carbon. As a control, *A. wodanis *and *Vibrio splendidus *were grown in parallel. None of the *A. salmonicida *isolates showed growth on (GlcNAc)_n _nor on GlcNAc [see Additional file 9]. In contrast, the majority of controls grew on both the homopolymeric and monomeric form of GlcNAc. This implies that the loss of seven genes involved in the chitinolytic cascade have probably affected processes such as sensing, degradation and transport of chitin and suggests that the ability to catabolise chitin is no longer required by *A. salmonicida*. Consistent with these findings preliminary studies looking for *A. salmonicida *in the environment have failed to find this species associated with copepods (personal communication B. Landfald). Accordingly, this could also confine the variety of carrier organisms *A. salmonicida *can attach to, and associate with.

It should be mentioned that programmed frameshifting and readthrough of premature stop codons are often used as methods of bacterial gene regulation [46]. In addition, homopolymeric DNA tracts can give rise to slipped-strand mispairing during replication [47]. It is therefore possible that some of the predicted pseudogenes could be translated into functional products, and are retained in the genome for selective reasons. Two flagellar biosynthesis genes, *fliF *(VSAL_I2308) and *flaG *(VSAL_I2316) are disrupted by premature stop codons. While the function of *flaG *is unknown, the product of *fliF *is the major component of the M-ring, a central motor component of the flagellum. Despite the disruption of *fliF *and *flaG *the sequenced strain is still motile. This could imply that these genes are not essential in *A. salmonicida*, or that the translational machinery is able to read through the premature stop codons and produce functional products.

### IV. Quorum sensing

Bacterial cell-to-cell communication, or quorum sensing (QS) is a sophisticated mechanism that can allow for a synchronized gene expression of a whole community. Bacteria can respond to environmental changes by monitoring the presence of other bacteria in the surroundings by producing and responding to extracellular signal molecules (autoinducers). *A. salmonicida *has five QS systems (AinR/S, LuxI/R, VarS/A, LuxM/N and LuxS/PQ), which is more than reported in any other *Vibrionaceae *[48]. However, there is extensive evidence of gene loss in these systems: *luxN *and *luxP *encoding the autoinducer receptors of the LuxM/N and LuxS/PQ systems, respectively are pseudogenes. In addition, *A. salmonicida *lacks *luxM *and *luxL*, required for the production of N-(3-hydroxylbutanyol)-L-homoserine lactone (HHL), the autoinducer of the LuxM/N system [49] further indicating that this system is non-functional. However, since the frameshift within *luxN *occurs within a homopolymeric tract (of 6 bp) it is possible that the function of this gene could be restored by programmed frameshifting. In the absence of LuxM and LuxL this would allow the system to function as a "mute" system monitoring the presence of HHLs produced by other bacteria.

### V. Potential virulence factors

Little is known about the molecular mechanisms by which *A. salmonicida *causes disease. Through detailed analysis of the genome possible functions that may be associated with mediating CV have been predicted [see Additional file 10].

The roles of several important virulence factors have been described for other *Vibrionaceae*, such as the cholera toxin (CT) of *V. cholerae *[50], the thermostable hemolysin (TDH) of *V. parahaemolyticus *[51] and the metalloprotease (VVP) of *V. vulnificus *[52]. Common for CT, TDH and VVP is that they act extracellularly, and are exported from the cell by various secretion mechanisms. Although none of these factors were found in *A. salmonicida*, the tissue damage observed in fish with CV suggests that *A. salmonicida *secrets proteins during an infection like these other pathogens. Several protein secretion systems were identified in the genome, including three Type I secretion systems (T1SS), one Type II secretion system (T2SS), two Type VI secretion systems (T6SS) and one Flp-type pilus system.

The CDSs of the Flp-type pilus are harboured on GI-VSA5 and show sequence similarities and high synteny to the Tad (tight adherence) macromolecular transport system of *Actinobacillus actinomycetemcomitans*. The *tad *system is widely distributed in bacteria and secrets a pilus that is involved in adherance to surfaces [53]. This function is necessary for colonization and pathogenesis by *A. actinomycetemcomitans*. The *tad *genes are present and intact in *A. salmonicida, A. fischeri*,*V. parahaemolyticus *and an incomplete operon is found in both *V. vulnificus *strains sequenced.

Functional gene-loss is evident in one T1SS, and both T6SSs gene clusters [see Additional file 10]. The products of the pseudogenes of the T6SSs are not predicted to be structural components of the secretion apparatus [54]. It is therefore possible that these systems are functional in *A. salmonicida*. T6SSI and T6SSII are located on GI-VS5 and GI-VS6, respectively. Both systems show sequence similarities as well as considerable synteny to the *V. cholerae *T6SS [54]. Virulence effector proteins secreted by T6SS lack an N-terminal signal sequence and include a hemolysin co-regulated protein (Hcp) and a VgrG protein [54]. By sequence similarity we identified three VgrG (VSAL_I1358, VSAL_p840_36 and VSAL_I1744) and three Hcp (VSAL_I1357 and VSAL_I1202) homologs in the genome. VSAL_I1744 is disrupted by the insertion of an IS-element and is probably not expressed.

Among the predicted CDSs with the potential to cause tissue degradation and hemolysis in the fish, we have identified two CDSs, VSAL_I0993 and VSAL_I0411 with 77% and 52% sequence identity to *V. anguillarum *hemolysins VAH2 and VAH5 respectively [see Additional file 10]. VAH2 and VAH5 showed hemolytic activity against fish erythrocytes and are suggested to contribute to the hemolytic activity of *V. anguillarum *[55]. To what extent VSAL_I0993 and VSAL_I0411 can cause hemolysis of fish blood cells, as observed in fish with CV is unknown. Similar to VAH2 and VAH5, no export signal sequence was found for VSAL_I0993 and VSAL_I0411. It is possible that the two putative hemolysins are exported by one or several of the *A. salmonicida *T1SS. In *E. coli*, export of hemolysin HlyA is mediated by the hemolysin secretion system, which has been described as one of the prototypes of T1SS [56].

*A. salmonicida *uses the siderophore bisucaberin to acquire iron [57]. A complete siderophore biosynthesis/acquisition system is contained on GI-VSA3 (VSAL_I0141-I0135), but whether it could be responsible for the production of bisucaberin remains to be clarified. We have also identified a heme uptake system with high sequence similarity and synteny to that of many other *Vibrionaceae *[58]. Both transport of heme complexes, and ferric-siderophores across the outer membrane require a functional TonB system. Several members of *Vibrionaceae *possess two TonB systems [59,60]. *A. salmonicida *harbours three TonB systems, named TonB1, TonB2 and TonB3. In *V. cholerae *both TonB systems corresponding to *A. salmonicida *TonB1 and TonB2 are capable of mediating the transport of heme and siderophores [59], while in *V. anguillarum *only the TonB system homologous to *A. salmonicida *TonB3 is essential for the ferric-siderophore transport and virulence [60]. In the TonB1 system, *tonB1 *(VSAL_I1751) contains a translational frameshift and is probably not translated into a functional product. All three TonB systems in *A. salmonicida *are located adjacent to CDSs with functions associated with iron-uptake. This indicates that more than one system may be involved in iron acquisition.

## Conclusion

The *A. salmonicida *genome displays a mosaic structure (Figure 1) caused by large intra-chromosomal rearrangements, gene acquisition, deletion and duplication of DNA within the chromosomes and between the chromosomes and the plasmids. From our sequence analysis it is clear that many of these events are mediated by homologous recombination between IS elements.

Multiple lines of evidence, such as compositional sequence differences, were used to identify recent gene acquisitions. The majority of the horizontally acquired DNA is flanked by IS elements. Although the direct influence the gene acquisitions have had on the evolution and adaptation of *A. salmonicida *is not clear, some of the GIs carry genes that may have provided new functions to the bacteria. For example, two T6SS and one Flp-type pilus system that are involved in the export of proteins are located on DNA segments that have the typical characteristics of GIs. T6SS have been recognized as a major virulence determinant in other pathogens were they have been shown to be involved in the extracellular translocation of proteins required for cytotoxicity [54,61]. The Flp-pilus system is similar to the Tad macromolecular transport system of *A. actinomycetemcomitans*. The Tad system has been proposed to represent a new subtype of T2SS and is essential for biofilm formation, colonization and pathogenesis [53]. Phylogenetic analysis of the Tad system shows a complex history of gene shuffling and multiple HGT among prokaryotes [62]. Our findings support the hypothesis that the distribution of the *tad *genes is explained by their location on a mobile GI (widespread colonisation island, WCI) [53]. Whether the protein secretion systems are important for the virulence towards fish remains to be elucidated.

Over 300 CDSs are disrupted by IS elements or contain point mutations causing frameshifts or premature stop codons [Additional file 11]. A large fraction of the degenerate CDSs have roles in the response to environmental changes and in modulating the host-cell interaction. The extensive loss of the same types of genes has been reported for the pathogen species *Mycobacterium leprae*, *Salmonella Typhi*, *Bordetella pertussis*, and others which have become host adapted [26,63]. The DNA sequences of these CDSs are still intact, which indicates that the gene losses are relatively recent events in *A. salmonicida*. IS expansion has been related to genome reduction in the evolution and emergence of pathogenicity [64], and accumulation of pseudogenes has been described for several other host-restricted pathogens [26,28,65], supporting the hypothesis that *A. salmonicida *may have also become host-restricted through gene loss.

Taken together, the acquisition of novel genes and loss of old functions may be related to the emergence of *A. salmonicida *as a pathogenic species for salmonids. The outcome of the horizontal acquisition of genes could have allowed for an expansion to a previously unexplored niche, and the accumulation of pseudogenes and IS expansion resulting in massive loss of functional genes observed in *A. salmonicida *may be a result of selection against the expression of genes not required in the new niche, or a neutral process associated with the relaxation of selective pressure due to the evolutionary bottleneck associated with niche adaptation. The observations made for the *A. salmonicida *genome are similar to those of other recently-evolved host-restricted pathogens, suggesting that *A. salmonicida *has recently made the transition to the specific niche of fish pathogenicity.

## Methods

We applied the whole-genome shotgun strategy to sequence an environmental isolate of *A. salmonicida *(strain LFI1238) from cod provided by Elin Sandaker at The Norwegian Institute of Fisheries and Aquaculture Ltd. A single colony of LFI1238 grown on blood agar containing 2.5% NaCl was transferred to marine broth and grown overnight with shaking at 12°C. Cells were collected and total DNA (10 mg) was isolated using proteinase K treatment followed by phenol extraction. The DNA was fragmented by sonication, and several libraries were generated in pUC19 and pMAQ1Sac using size fractions ranging from 2.2 to 4.0 kb and 4.0 to 12.0 kb, respectively. The whole genome was sequenced to a depth of 10 times coverage using dye terminator chemistry on ABI3700 automated sequencers. End sequences from larger insert plasmid (pBeloBACII, 50–70 kb insert size) libraries were used as a scaffold.

The sequence was annotated using Artemis software [66]. Initial CDS predictions were performed using Orpheus [67] and Glimmer2 [68] software. These predictions were amalgamated, and codon usage, positional base preference methods and comparisons to the non redundant protein databases using BLAST [69] and FASTA [70] software were used to refine the predictions. The entire DNA sequence was also compared in all six reading frames against the nonredundant protein databases, using BLASTX to identify any possible coding sequences previously missed. Protein motifs were identified using Pfam [71] and Prosite [72], transmembrane domains were identified with TMHMM [73], and signal sequences were identified with SignalP version 2.0 [74]. Stable RNAs were identified using Rfam [75]. GIs and bacteriophages were predicted using Alien Hunter [76]. The sequence is available from EMBL/GenBank/DDBJ with the accession numbers [EMBL: FM178379, FM178380, FM178381, FM178382, FM178383 and FM178384].

Comparison of the genome sequences was facilitated by using the Artemis Comparison Tool (ACT) [77], which enabled the visualization of BLASTN and TBLASTX comparisons [69] between the genomes. Orthologous proteins were identified as reciprocal best matches using FASTA with subsequent manual curation. Pseudogenes had one or more mutations that would prevent correct translation and each of the inactivating mutations were subsequently checked against the original sequencing data.

In order to determine if duplicated genes originated from the plasmids or from the chromosomes and to predict the order in which the plasmids were acquired we performed a CAI and a CBI analysis: CAI and a CBI analysis: CAI used the Highly expressed genes (encoding all ribosomal proteins and tRNA synthetases in the genome) as the reference; CBI used the codon usage of all the genes in the genome and measured the adaptation of each gene to that. The CAI was done via EMBOSS cai and the CBI was done via EMBOSS codcmp [27].

Amplification of genes from other isolates was performed by PCR using Platinum Pfx DNA Polymerase (Invitrogen, Carlsbad, CA) according to the protocol supplied by the manufacturer. PCR amplification products were analyzed in 0.8% agarose gels stained with ethidium bromide.

Isolates of *A. salmonicida*, *A. wodanis *and *V. splendidus *were grown in LB medium containing 2.5% NaCl, diluted in *A. salmonicida *minimal medium (Vsmm [100 mM KH_2_PO_4_, 15 mM (NH_4_)_2_SO_4_, 3.9 μM FeSO_4_, 2.5% NaCl, 0.81 mM MgSO_4_, 2 mM Valin, 0.5 mM Isoleucin, 0.5 mM Cystein, 0.5 mM Methionin, 40 mM Glutamate]) and transferred to Vsmm agar supplemented with 10 mg/ml α-chitin (Sigma-Aldrich) or N-acetyl-α-D-glucosamine (Calbiochem). Plates were incubated from 2 to 7 days at 12°C (*A. salmonicida *and *A. wodanis*) and 22°C (*V. splendidus*) and growth evaluated by visual examination.

## Abbreviations

IS: insertion sequence; CV: cold-water vibriosis; chr: chromosome; CDS: protein coding sequence; CAI: Codon Adaptation Index; CBI: Codon Bias Index; CPS: capsular polysaccharides; LPS: lipopolysaccharide; (GlcNAc): N-acetyl-D-glucosamine; CBP: chitin binding protein; QS: quorum sensing; HGT: horizontal gene transfer; GI: genomic island.

## Authors' contributions

EH: study conception, data analysis, research design, manuscript writing. MSL: research design, data collection, manuscript production. MTGH: research design, manuscript production. KS: data collection. SP: research design, manuscript production. NB: data collection. CC: data collection. DH: data collection. HN: data collection. MAQ: data collection. SS: data collection. ST: data collection. JP: study conception, manuscript production. NPW: study conception, manuscript production. NRT: research design, study conception, manuscript writing.

## Supplementary Material

Additional file 1 **A: Schematic circular diagrams of *A. salmonicida *LFI1238 plasmids; B: Putative duplicated regions of the plasmids in comparison to chromosome I.** A: Appropriate categories are shown as pairs of concentric circles representing both coding strands. Key to the chromosomal circular diagrams (outside to inside): scale (in kb), annotated CDSs, unique CDSs compared to the other *Vibrionaceae *species (red), orthologues shared with the other *Vibrionaceae *species (green), IS element transposases (purple), % G+C content, G+C deviation (>0% olive, <0% purple). Colour coding for CDSs (according to predicted function): dark blue, pathogenicity/adaptation; black, energy metabolism; red, information transfer; dark green, surface associated; cyan, degradation of large molecules; magenta, degradation of small molecules; yellow, central/intermediary metabolism; pale green, unknown; pale blue, regulators; orange, conserved hypothetical; brown, pseudogenes; pink, phage + IS elements; grey, miscellaneous. B: CDSs are represented as blocked arrows showing the direction of transcription. Identity at nucleotide level is indicated in grey boxes. Click here for file

Additional file 2 **Types and distribution of IS elements encoded in the *A. salmonicida *genome.** The data provided shows the distribution of the different types of IS elements identified in the six replicons. Click here for file

Additional file 3 **Codon Adaptation Index (CAI) of genes plotted against Codon Bias Index (CBI).** Colour coding for the genes are: grey, chromosomal genes; light blue, highly expressed genes (encoding ribosomal proteins and tRNA synthetases); red, pVSAL840; dark green, pVSAL320; yellow, pVSAL54; light green, pVSAL43; black triangles, duplicated genes. Click here for file

Additional file 4 **Linear DNA comparison between the chromosomes of *A. salmonicida *and *A. fischeri*.** The grey bars represent the forward and reverse strands, and red and blue lines between the genomes indicate regions with similarity and inversions, respectively. Black boxes represent IS elements in *A. salmonicida*. Click here for file

Additional file 5 **Genomic organisation of the *A. salmonicida *inserted phage ϕ VS4 and comparison with the related phage K139.** The red lines between the phages indicate regions with amino acid similarity. CDSs are represented as blocked arrows showing the direction of transcription, with colour codes according to their functional annotation. The length of the arrows approximately reflects the sizes of the CDSs. The G+C content and G+C average was analysed using Artemis with a window size of 500 nt. Click here for file

Additional file 6 **CDSs harboured on putative genomic islands and phages in *A. salmonicida*.** The data in this table is additional to the data in Table 2, and provides a complete overview of all CDSs harboured on the putative genomic islands and phages listed in Table 2. Click here for file

Additional file 7 **A: The two duplicated regions in *A. salmonicida *LFI1238; B: Agarose gel electrophoresis of PCR products confirming the presence of the duplication in all tested *A. salmonicida *isolates.** The first gene of each duplicate and the flanking genes are indicated as grey boxes (not drawn to scale). B) Agarose gel electrophoresis of duplication products VSAL_I0408-I0407 (PCR product 1) and VSAL_I0264-I0265 (PCR product 2), and the corresponding regions of different *A. salmonicida *isolates derived by PCR from genomic DNA. Primers are designed from LFI1238 and are indicated by arrows (p1 5'-CGACATGATCGTGTTTTGCT-3', p2 and p3 5'-GGAAAATAGCATCAATTGTA-3', p4 5'-CCATTGTAGAGGTGAGTTTA-3'). The *A. salmonicida *isolates were obtained from various outbreaks of cold water vibriosis from salmon and cod. The isolate numbers are indicated above each lane. S, 100 bp DNA ladder. Click here for file

Additional file 8 **Functional and putative non-functional *A. salmonicida *genes involved in key steps of the chitinolytic cascade, together with orthologues from other sequenced *Vibrionaceae*.** The data provided in this table lists all identified *A. salmonicida *CDSs that are known to be involved in the chitinolytic cascade in other *Vibrionaceae*. Disrupted CDSs and pseudogenes are labelled in the table. Click here for file

Additional file 9 **Growth of different *Vibrionaceae *isolates on α-chitin and GlcNAc.** The data provided shows that the *A. salmonicida *isolates investigated in this study are not able to utilise α-chitin or GlcNAc. Click here for file

Additional file 10 **Predicted CDSs of *A. salmonicida *with potential roles in pathogenicity.** The data in this table gives a comprehensive list of all CDSs with possible functions that may be associated with mediating cold-water vibriosis in fish. Click here for file

Additional file 11 **Functional distribution of putative inactivated genes in the *A. salmonicida *genome.** The data provided in this table includes all CDSs disrupted or truncated by IS elements and CDSs containing translational frameshifts of premature stop codons. Click here for file
