# Supplementary material for: The genome sequence of the fish pathogen Aliivibrio salmonicida strain LFI1238 shows extensive evidence of gene decay
Source: BMC Genomics. 2008 Dec 19;9:616. doi: 10.1186/1471-2164-9-616 (PMC2627896; doi:10.1186/1471-2164-9-616)

Additional file 1. A: Schematic circular diagrams of *A. salmonicida* LFI1238 plasmids; **B:** Putative duplicated regions of the plasmids in comparison to chromosome I.

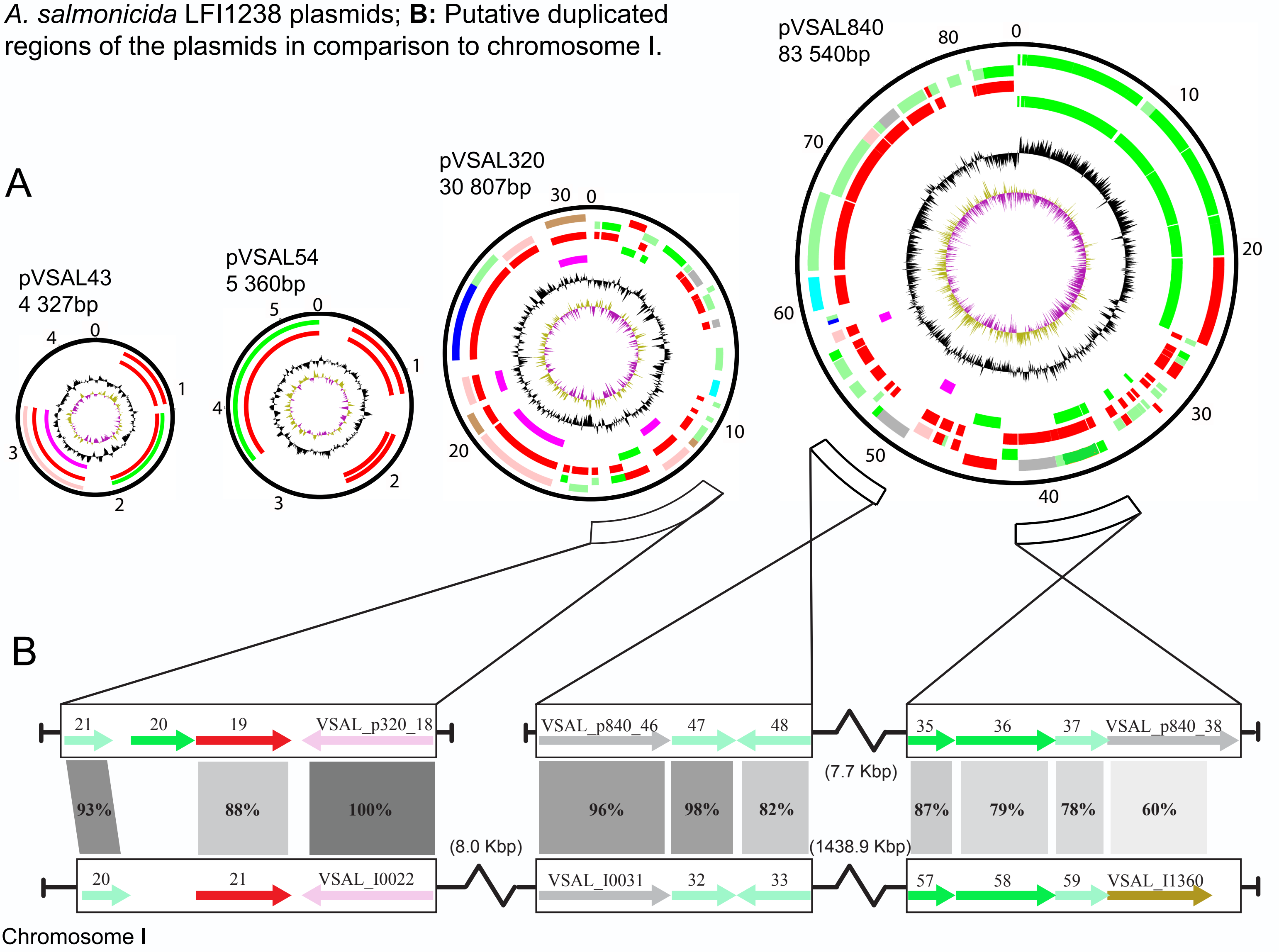

Supplement: Additional file 1 — A: Schematic circular diagrams of A. salmonicida LFI1238 plasmids; B: Putative duplicated regions of the plasmids in comparison to chromosome I. A: Appropriate categories are shown as pairs of concentric circles representing both coding strands. Key to the chromosomal circular diagrams (outside to inside): scale (in kb), annotated CDSs, unique CDSs compared to the other Vibrionaceae species (red), orthologues shared with the other Vibrionaceae species (green), IS element transposases (purple), % G+C content, G+C deviation (>0% olive, <0% purple). Colour coding for CDSs (according to predicted function): dark blue, pathogenicity/adaptation; black, energy metabolism; red, information transfer; dark green, surface associated; cyan, degradation of large molecules; magenta, degradation of small molecules; yellow, central/intermediary metabolism; pale green, unknown; pale blue, regulators; orange, conserved hypothetical; brown, pseudogenes; pink, phage + IS elements; grey, miscellaneous. B: CDSs are represented as blocked arrows showing the direction of transcription. Identity at nucleotide level is indicated in grey boxes. [file 1471-2164-9-616-S1.pdf]
