# Supplementary material for: The genome sequence of the fish pathogen Aliivibrio salmonicida strain LFI1238 shows extensive evidence of gene decay
Source: BMC Genomics. 2008 Dec 19;9:616. doi: 10.1186/1471-2164-9-616 (PMC2627896; doi:10.1186/1471-2164-9-616)
Supplement: Additional file 2 — Types and distribution of IS elements encoded in the A. salmonicida genome. The data provided shows the distribution of the different types of IS elements identified in the six replicons. [file 1471-2164-9-616-S2.pdf]

Additional file 2. Types and distribution of IS elements encoded in the *A. salmonicida* genome.

Type	IS family*	CDSs	Chromosome I	Chromosome II	pVSAL840	pVSAL320	pVSAL54	pVSAL43	Total copies	Total ORFs
VSa1	IS4	1	12 (2)	2 (1)					14 (3)	14 (3)
VSa2	IS4, IS66	3	74 (10)	37 (13)					111 (23)	313 (15)
VSa3	IS91	1			1				1	1
VSa4	IS6	1			1				1	1
VSa5	IS4	1	1						1	1
VSa6	IS982	1	45 (2)	14					59 (2)	59 (2)
VSa7	IS30	1	6	3					9	9
VSa8	IS630	2	17	10					27	54
VSa9	IS91	1	22 (1)	23 (3)		3		1	49 (4)	49 (4)
VSa10	IS91	1		2					2	2
VSa11	IS3	2	1	1					2	4
VSa12	IS3	1		1 (1)					1 (1)	1 (1)
VSa13	ISAs1	1	1						1	1
VSa14	ISAs1	1	3						3	3
VSa15	IS5	1	1						1	1
VSa16	IS630	1	1						1	1
VSa17	ISNCY	1	3						3	3
VSa18	IS5	2	1						1	2
VSa19	IS5	1				1			1	1
VSa20	IS5	1	1 (1)						1 (1)	1 (1)
			**188 (13)**	**93 (18)**	**2**	**4**		**1**	**288 (33)**	**521 (26)**

*Based on similarities to defined families in the IS Finder database (http://www-is.biotoul.fr/is.html)

() Disrupted or partial IS elements and transposase genes
