# Supplementary figures and images for: The genome sequence of the fish pathogen Aliivibrio salmonicida strain LFI1238 shows extensive evidence of gene decay
Source: BMC Genomics. 2008 Dec 19;9:616. doi: 10.1186/1471-2164-9-616 (PMC2627896; doi:10.1186/1471-2164-9-616)

**Additional file 3.** Codon Adaptation Index (CAI) of genes plotted against Codon Bias Index (CBI).

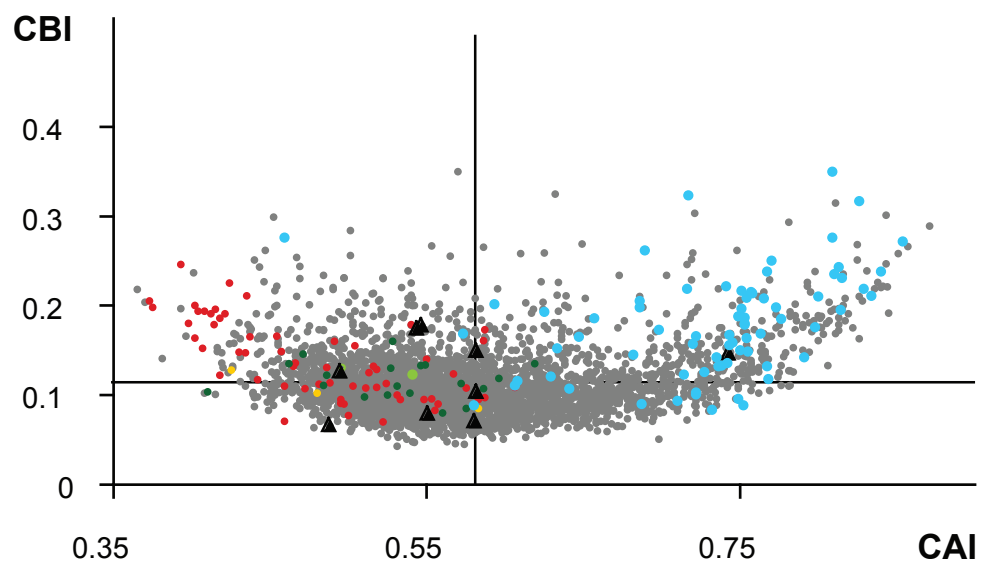

Supplement: Additional file 3 — Codon Adaptation Index (CAI) of genes plotted against Codon Bias Index (CBI). Colour coding for the genes are: grey, chromosomal genes; light blue, highly expressed genes (encoding ribosomal proteins and tRNA synthetases); red, pVSAL840; dark green, pVSAL320; yellow, pVSAL54; light green, pVSAL43; black triangles, duplicated genes. [file 1471-2164-9-616-S3.pdf]
