# Supplementary material for: The genome sequence of the fish pathogen Aliivibrio salmonicida strain LFI1238 shows extensive evidence of gene decay
Source: BMC Genomics. 2008 Dec 19;9:616. doi: 10.1186/1471-2164-9-616 (PMC2627896; doi:10.1186/1471-2164-9-616)

Additional file 4. Linear DNA comparison between the chromosomes of *A. salmonicida* and *A fischeri*.

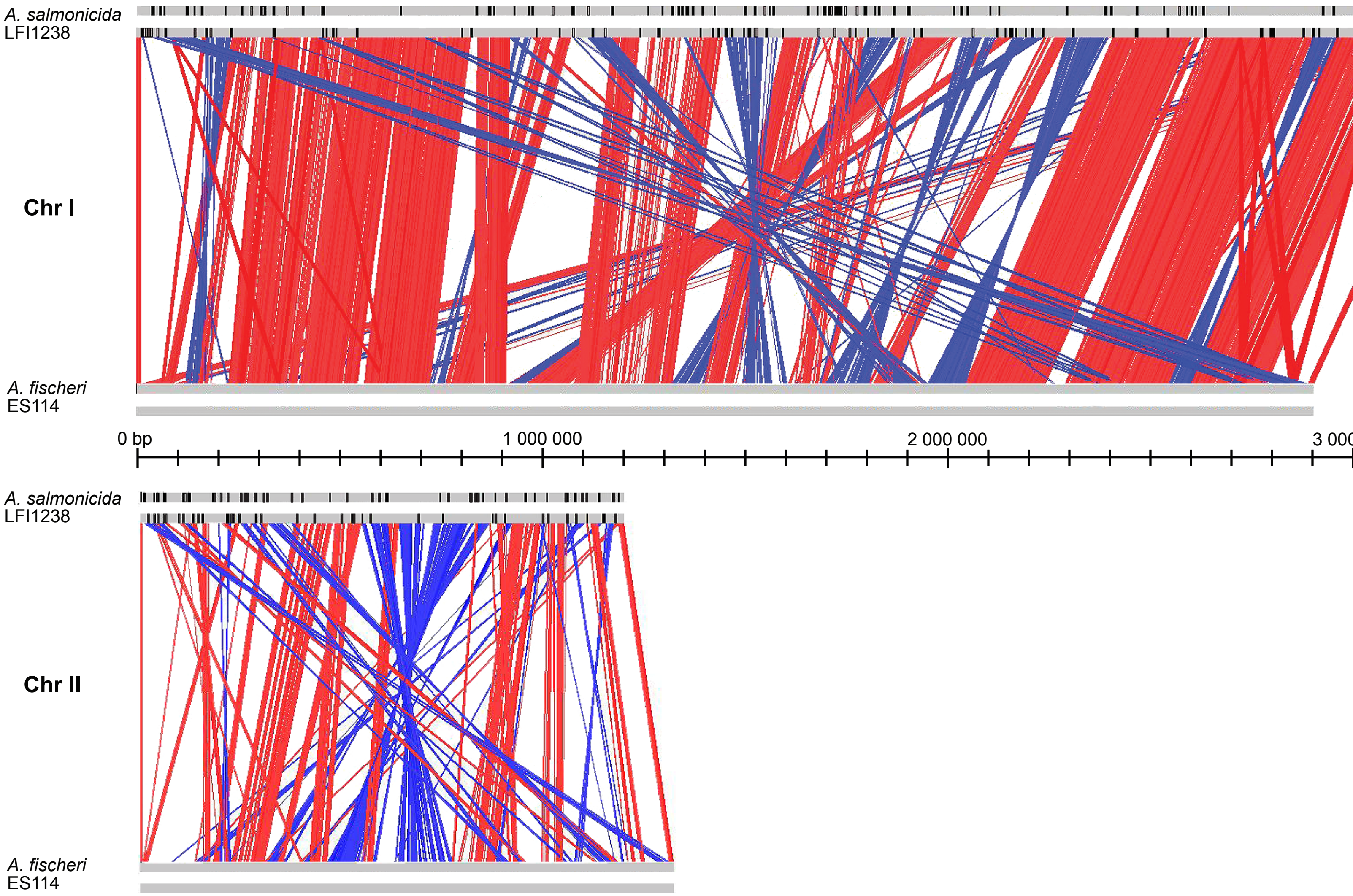

Supplement: Additional file 4 — Linear DNA comparison between the chromosomes of A. salmonicida and A. fischeri. The grey bars represent the forward and reverse strands, and red and blue lines between the genomes indicate regions with similarity and inversions, respectively. Black boxes represent IS elements in A. salmonicida. [file 1471-2164-9-616-S4.pdf]
