# Supplementary material for: The genome sequence of the fish pathogen Aliivibrio salmonicida strain LFI1238 shows extensive evidence of gene decay
Source: BMC Genomics. 2008 Dec 19;9:616. doi: 10.1186/1471-2164-9-616 (PMC2627896; doi:10.1186/1471-2164-9-616)

Additional file 5. Genomic organisation of the *A. salmonicida* inserted phage ϕVS4 and comparison with the related phage K139.

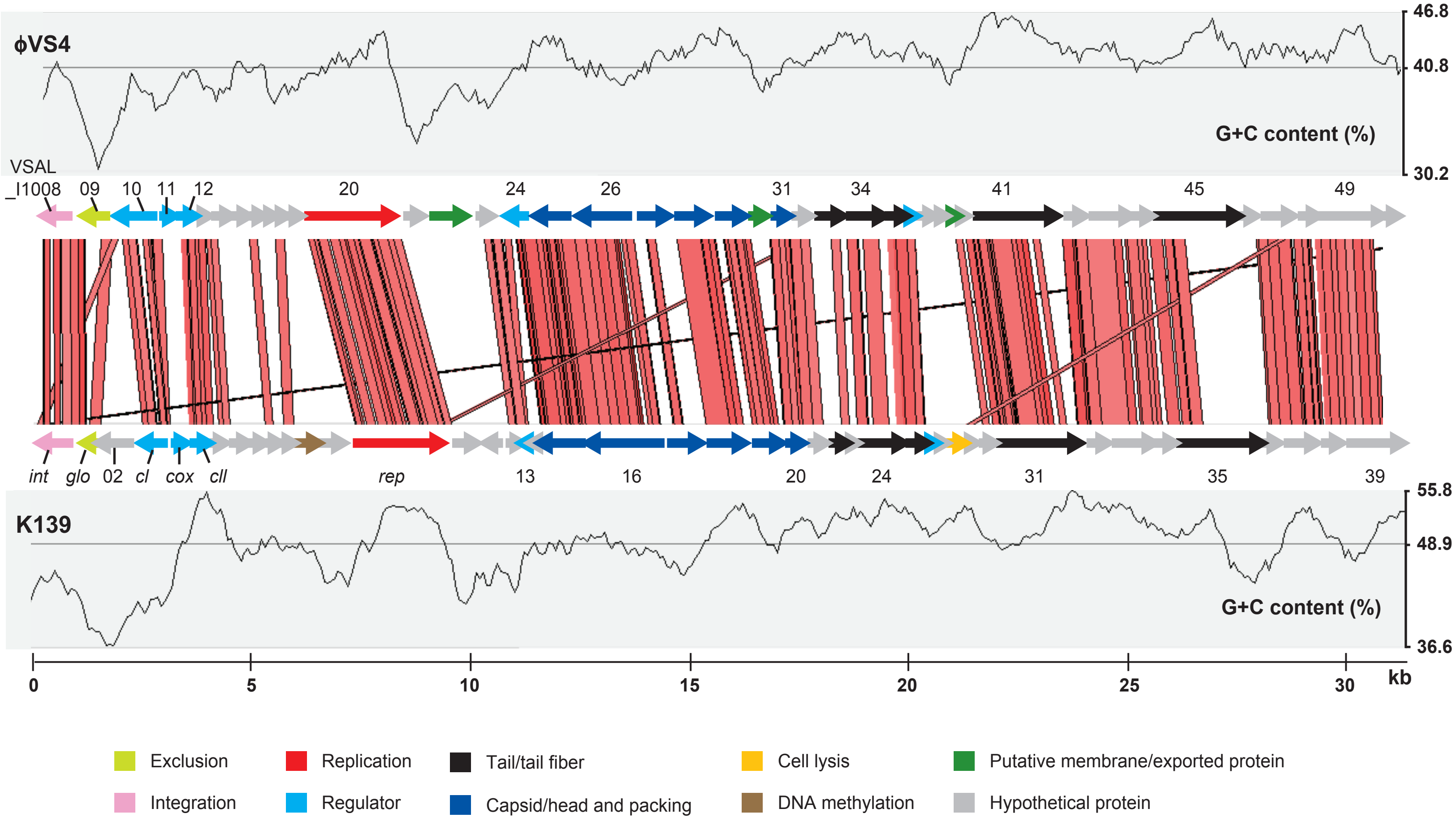

Supplement: Additional file 5 — Genomic organisation of the A. salmonicida inserted phage ϕ VS4 and comparison with the related phage K139. The red lines between the phages indicate regions with amino acid similarity. CDSs are represented as blocked arrows showing the direction of transcription, with colour codes according to their functional annotation. The length of the arrows approximately reflects the sizes of the CDSs. The G+C content and G+C average was analysed using Artemis with a window size of 500 nt. [file 1471-2164-9-616-S5.pdf]
