# Supplementary material for: The genome sequence of the fish pathogen Aliivibrio salmonicida strain LFI1238 shows extensive evidence of gene decay
Source: BMC Genomics. 2008 Dec 19;9:616. doi: 10.1186/1471-2164-9-616 (PMC2627896; doi:10.1186/1471-2164-9-616)
Supplement: Additional file 6 — CDSs harboured on putative genomic islands and phages in A. salmonicida. The data in this table is additional to the data in Table 2, and provides a complete overview of all CDSs harboured on the putative genomic islands and phages listed in Table 2. [file 1471-2164-9-616-S6.pdf]

Additional file 6. CDSs harboured on putatative genomic islands and phages in *A. salmonicida*.

CDS	Size	Predicted product	Similar to	Acc. No.	Identity (%)	E-value
**Chr I**						
**ϕVS1**						
VSAL_I0388	93	hypothetical protein, putative phage gene	*None*			
VSAL_I0389	279	hypothetical protein, putative phage gene	*Vibrio parahaemolyticus*	Q8L0W3	64	2.60E-25
VSAL_I0390	130	hypothetical protein, putative phage gene	*None*			
VSAL_I0391	341	hypothetical protein, ADP-ribose binding protein	*Marinobacter sp.*	A3JFC2	36	4.30E-36
VSAL_I0392	71	hypothetical protein, putative phage gene	*None*			
VSAL_I0393	140	transposase	*Hahella chejuensis*	Q2SAQ2	84	2.70E-45
VSAL_I0394	188	transposase	*Hahella chejuensis*	Q2SMC4	47	1.10E-34
VSAL_I0395*	117	hypothetical protein, putative phage gene	*None*			
VSAL_I0396	358	hypothetical protein, putative phage gene	*Escherichia coli*	A1Z2R1	36	1.40E-38
VSAL_I0397	353	shufflon-specific DNA recombinase	*Escherichia coli*	P16470	36	1.00E-14
VSAL_I0398	356	shufflon-specific DNA recombinase	*Escherichia coli*	P10487	30	1.30E-11
**ϕVS2**						
VSAL_I0764*	495	transposase	*Shewanella sp.*	A1RLT8	57	2.10E-119
VSAL_I0765	115	transposase	*Pseudoalteromonas haloplanktis*	Q3IIG1	58	1.40E-31
VSAL_I0766	104	transposase	*Pseudoalteromonas haloplanktis*	Q3IIG2	30	4.10E-06
VSAL_I0767*	48	hypothetical protein, putative phage gene	*Bacteriophage Mu*	P79677	49	0.0037
VSAL_I0768	176	hypothetical protein, putative phage gene	*Aliivibrio fischeri*	Q5E392	52	1.60E-35
VSAL_I0769	157	hypothetical protein, putative phage gene	*Aliivibrio fischeri*	Q5E391	58	3.00E-39
VSAL_I0770	152	hypothetical protein, putative phage gene	*Aliivibrio fischeri*	Q5E390	63	1.30E-35
VSAL_I0771	344	phage major capsid protein	*Bacteriophage PK*	Q94MC7	39	7.90E-38
VSAL_I0772	290	probable capsid scaffolding protein	*Bacteriophage HP1*	P51719	36	1.00E-15
VSAL_I0773	598	putative bacteriophage terminase	*Pseudomonas phage phi CTX*	Q9ZXM5	39	7.20E-74
VSAL_I0774*	316	putative portal vertex protein	*Bacteriophage P2*	P25480	41	6.20E-09
VSAL_I0775	337	putative exported protein, putative phage gene	*None*			
VSAL_I0776	153	hypothetical protein, putative phage gene	*Aliivibrio fischeri*	Q5E1J4	61	5.70E-32
VSAL_I0777	251	N-acetylmuramoyl-L-alanine amidase	*Yersinia pestis bv. Antiqua*	Q1C6W7	65	4.10E-68
VSAL_I0778	120	hypothetical protein, putative phage gene	*None*			
VSAL_I0779	56	putative membrane protein, putative phage gene	*None*			
VSAL_I0780	132	putative membrane protein, putative phage gene	*Bacteriophage T5*	Q6QGP7	38	8.20E-05
VSAL_I0781	104	transposase	*Pseudoalteromonas haloplanktis*	Q3IIG2	30	4.10E-06
VSAL_I0782	115	transposase	*Pseudoalteromonas haloplanktis*	Q3IIG1	58	1.40E-31
VSAL_I0783	495	transposase	*Shewanella sp.*	A1RLT8	57	1.10E-119

**ϕVS3**						
VSAL_I0966	453	putative phage recombinase/integrase	*Bacillus subtilis*	P39776	16	3.00E-04
VSAL_I0967	173	hypothetical protein, putative phage gene	*Vibrio parahaemolyticus*	A6B1Z2	58	1.20E-32
VSAL_I0968	807	putative site-specific recombinase	*Vibrio alginolyticus*	Q1V881	49	2.10E-77
VSAL_I0968	779	putative exported protein, putative phage gene	*Vibrio alginolyticus*	Q1V882	44	6.00E-134
**ϕVS4**						
VSAL_I1008	347	phage integrase	*Bacteriophage 186*	P06723	47	2.60E-53
VSAL_I1009	262	probable exclusion protein	*Vibrio phage K139*	Q37932	29	1.70E-06
VSAL_I1010	318	probable phage regulatory protein CI	*Vibrio phage K139*	Q9XJF9	47	4.10E-21
VSAL_I1011	66	probable phage regulatory protein	*Haemophilus phage HP2*	Q775G1	32	0.16
VSAL_I1012	180	phage regulatory protein CII	*Vibrio phage K139*	Q9XJF7	67	3.00E-44
VSAL_I1013	107	hypothetical protein, putative phage gene	*Vibrio phage K139*	Q8W766	38	6.00E-08
VSAL_I1014	160	hypothetical protein, putative phage gene	*Vibrio phage K139*	Q8W765	29	1.00E-04
VSAL_I1015	133	hypothetical protein, putative phage gene	*Vibrio phage K139*	Q8W764	36	5.4
VSAL_I1016	42	hypothetical protein, putative phage gene	*None*			
VSAL_I1017	90	hypothetical protein, putative phage gene	*Bacteriophage phi1026b*	Q6JIJ0	30	0.22
VSAL_I1018	76	hypothetical protein, putative phage gene	*Vibrio phage K139*	Q8W762	33	1.90E-07
VSAL_I1019	119	hypothetical protein, putative phage gene	*None*			
VSAL_I1020	837	phage replication protein	*Bacteriophage 186*	P41064	42	1.00E-58
VSAL_I1021	384	hypothetical protein, putative phage gene	*None*			
VSAL_I1022	304	membrane protein, putative phage gene	*Aliivibrio fischeri*	Q5E372	83	4.60E-91
VSAL_I1023	84	hypothetical protein, putative phage gene	*Vibrio phage K139*	Q8W756	50	9.40E-09
VSAL_I1024	154	phage zinc-binding transcriptional activator	*Vibrio phage K139*	Q8W755	56	5.20E-14
VSAL_I1025	344	phage portal protein	*Vibrio phage K139*	Q8W753	66	1.20E-85
VSAL_I1026	590	terminase, ATPase subunit	*Vibrio phage K139*	Q8W752	67	3.00E-143
VSAL_I1027	291	phage capsid scaffolding protein	*Vibrio phage K139*	Q8W751	49	4.00E-41
VSAL_I1028	336	major capsid protein	*Vibrio phage K139*	Q8W750	48	2.00E-52
VSAL_I1029	238	phage terminase, endonuclease subunit	*Vibrio phage K139*	Q8W749	51	2.70E-49
VSAL_I1030	113	membrane protein, putative phage gene	*None*			
VSAL_I1031	154	probable head completion/stabilization protein	*Vibrio phage K139*	Q8W748	51	2.50E-26
VSAL_I1032	174	hypothetical protein, putative phage gene	*Vibrio phage K139*	Q8W747	34	1.20E-15
VSAL_I1033*	236	putative tail completion protein	*Pseudomonas syringae pv. phaseolicola Prophage pspph06*	Q48C42	43	7.80E-11
VSAL_I1034	370	putative tail sheath protein	*Haemophilus phage HP2*	Q94MY9	51	9.30E-68
VSAL_I1035	151	probable tail tube protein	*Vibrio phage K139*	Q8W743	56	8.20E-27
VSAL_I1036	71	probable rRNA transcription initiatior protein, putative phage gene		Q8W742	51	5.90E-09
VSAL_I1037	140	hypothetical protein, putative phage gene	*Photobacterium profundum*	Q6LLE3	61	2.10E-19

VSAL_I1038	81	hypothetical protein, putative phage gene	*Vibrio harveyi*	A7N394	66	3.80E-19
VSAL_I1039	79	probable exported protein, putative phage gene	*Vibrio harveyi*	A7N395	66	1.70E-16
VSAL_I1040	87	hypothetical protein, putative phage gene	*Haemophilus phage HP2*	Q775G2	33	4.00E-05
VSAL_I1041	704	probable tail length determinator	*Vibrio phage K139*	Q8W737	56	3.20E-101
VSAL_I1042	108	hypothetical protein, putative phage gene	*Vibrio phage K139*	Q8W736	54	5.30E-13
VSAL_I1043	394	hypothetical protein, putative phage gene	*Vibrio phage K139*	Q8W735	51	1.00E-75
VSAL_I1044	196	hypothetical protein, putative phage gene	*Vibrio phage K139*	Q8W734	50	1.30E-35
VSAL_I1045	690	tail fiber protein	*Bacteriophage HP1*	P51735	28	2.20E-35
VSAL_I1046	147	hypothetical protein, putative phage gene	*Aliivibrio fischeri*	Q5E382	81	3.50E-42
VSAL_I1047	297	hypothetical protein, putative phage gene	*Vibrio phage K139*	Q8W731	42	1.00E-41
VSAL_I1048	182	hypothetical protein, putative phage gene	*Yersinia pseudotuberculosis*	Q666T6	39	1.40E-14
VSAL_I1049	541	hypothetical protein, putative phage gene	*Vibrio phage K139*	Q8W729	47	2.60E-82
VSAL_I1050	65	hypothetical protein, putative phage gene	*Aliivibrio fischeri*	Q5E377	86	1.00E-17
VSAL_I1051	293	transposase	*Shewanella denitrificans*	Q12IQ1		2.30E-75
**ϕVS5**						
VSAL_I1921	321	putative membrane protein, putative phage gene	*None*			
VSAL_I1922	741	putative exported protein, putative phage gene	*None*			
VSAL_I1923*	296	putative outer membrane protein OmpA, putative phage gene	*Vibrio alginolyticus*	Q1VCV6	32	4.20E-16
VSAL_I1924	206	putative exported protein, putative phage gene	*Enterobacteria phage phiEco32*	B0FIT1	40	9.00E-10
VSAL_I1925	208	immunoglobulin-binding regulator	*Escherichia coli*	Q8KU23	35	4.10E-17
VSAL_I1926*	380	immunoglobulin-binding regulator	*Escherichia coli*	Q8KU24	43	7.90E-44
VSAL_I1927	192	hypothetical protein, putative phage gene	*Aliivibrio fischeri*	Q5E397	43	9.70E-22
VSAL_I1928	104	transposase	*Pseudoalteromonas haloplanktis*	Q3IIG2	30	4.10E-06
VSAL_I1929	115	transposase	*Pseudoalteromonas haloplanktis*	Q3IIG1	58	1.40E-31
VSAL_I1930	495	transposase	*Shewanella sp.*	A1RLT8	57	1.10E-119
**ϕVS6**						
VSAL_I2480	413	integrase, phage family	*Vibrio cholerae*	Q57497	71	1.90E-117
VSAL_I2481	249	hypothetical protein, putative phage gene	*None*			
VSAL_I2482	976	putative ABC transporter, ATP-binding domain	*None*			
VSAL_I2483*	649	putative type I restriction enzyme R protein	*Escherichia coli*	P10486	35	5.50E-43
VSAL_I2484	104	transposase	*Pseudoalteromonas haloplanktis*	Q3IIG2	30	4.10E-06
VSAL_I2485	115	transposase	*Pseudoalteromonas haloplanktis*	Q3IIG1	58	1.40E-31
VSAL_I2486	495	transposase	*Shewanella sp.*	A1RLT8	57	1.10E-119
VSAL_I2487*	495	transposase	*Shewanella sp.*	A1RLT8	56	3.00E-117
VSAL_I2488	115	transposase	*Pseudoalteromonas haloplanktis*	Q3IIG1	58	1.40E-31
VSAL_I2489	104	transposase	*Pseudoalteromonas haloplanktis*	Q3IIG2	30	4.10E-06
**GI-VS1**						

VSAL_I0019	397	transposase	*Pasteurella piscicida*	A0PB90	52	9.70E-85
VSAL_I0020	84	hypothetical protein	*Aliivibrio fischeri*	Q5DY95	87	2.10E-30
VSAL_I0021	319	antirestriction protein	*Escherichia coli*	Q6XGE4	50	2.10E-49
VSAL_I0022	397	transposase	*Pasteurella piscicida*	A0PB90	52	9.70E-85
VSAL_I0023	281	metallo-beta-lactamase superfamily protein	*Shewanella pealeana*	A8H4L9	83	1.50E-102
VSAL_I0024	183	adenylate cyclase	*Aeromonas hydrophila*	O69199	64	2.40E-41
VSAL_I0025	121	putative exported protein	*Vibrio parahaemolyticus*	Q87PY1	55	6.40E-23
VSAL_I0026	134	hypothetical protein	*Vibrio alginolyticus*	Q1VCC3	67	6.80E-38
VSAL_I0027	259	hypothetical protein	*Escherichia coli*	Q0E860	24	2.00E-09
VSAL_I0028	317	hypothetical protein	*Photobacterium profundum*	Q6LWB9	94	5.00E-126
VSAL_I0029	320	transposase	*Photorhabdus luminescens*	Q8GDK9	58	3.00E-76
VSAL_I0030	96	hypothetical protein	*None*			
VSAL_I0031	739	putative cell wall degradation enzyme	*Aliivibrio fischeri*	A9HZV5	39	2.50E-63
VSAL_I0032	129	putative exported protein	*None*			
VSAL_I0033	119	hypothetical protein	*None*			
VSAL_I0034	216	hypothetical protein	*None*			
VSAL_I0035	397	transposase	*Pasteurella piscicida*	A0PB90	52	9.70E-85
**GI-VS2**						
VSAL_I0056	397	transposase	*Pasteurella piscicida*	A0PB90	52	9.70E-85
VSAL_I0057	175	putative exported protein	*None*			
VSAL_I0058	265	putative chromosome partitioning protein ParA	*Bacillus subtilis*	P37522	32	1.60E-13
VSAL_I0059	324	putative chromosome partitioning protein ParB	*Vibrio anguillarum*	Q83XH6	37	4.80E-31
VSAL_I0060	134	hypothetical protein	*Vibrio anguillarum*	Q6W4R4	49	1.20E-24
VSAL_I0061	123	hypothetical protein	*None*			
VSAL_I0062	197	hypothetical protein	*Photobacterium profundum*	Q6LWA8	37	2.00E-20
VSAL_I0063*	496	hypothetical protein	*Photobacterium profundum*	Q6LUB5	78	4.60E-147
VSAL_I0064*	76	hypothetical protein	*None*			
VSAL_I0065	118	transposase	*Vibrionales bacterium SWAT-3*	A5KV91	78	3.00E-43
VSAL_I0066	98	transposase	*Vibrionales bacterium SWAT-3*	A5KV90	42	0.00063
VSAL_I0067	409	putative hemolysin/hemagglutinin-like protein	*Erwinia chrysanthemi*	P94772	29	3.90E-08
VSAL_I0068	495	transposase	*Shewanella sp.*	A1RLT8	56	3.00E-117
VSAL_I0069	115	transposase	*Pseudoalteromonas haloplanktis*	Q3IIG1	58	1.40E-31
VSAL_I0070	104	transposase	*Pseudoalteromonas haloplanktis*	Q3IIG2	30	4.10E-06
**GI-VS3**						
VSAL_I0129	104	transposase	*Pseudoalteromonas haloplanktis*	Q3IIG2	30	4.10E-06
VSAL_I0130*	115	transposase	*Pseudoalteromonas haloplanktis*	Q3IIG1	65	1.10E-17
VSAL_I0131	397	transposase	*Pasteurella piscicida*	A0PB90	52	9.70E-85

VSAL_I0132	196	putative lipoprotein	*Vibrio sp.*	A3XXI3	67	5.50E-47
VSAL_I0133	483	hypothetical protein	*Vibrio parahaemolyticus*	Q87GL3	68	5.90E-130
VSAL_I0134	515	L-2,4-diaminobutyrate decarboxylase	*Rhizobium meliloti*	Q9Z3R1	36	3.00E-36
VSAL_I0135	442	siderophore biosynthetis protein IucD	*Escherichia coli*	Q47318	26	6.20E-41
VSAL_I0136	818	siderophore biosynthetis protein IucC	*Escherichia coli*	P11295	33	1.00E-45
VSAL_I0137	721	tonB-dependent iron-siderophore receptor FhuE	*Escherichia coli*	P16869	35	6.90E-71
VSAL_I0138	316	iron-siderophore ABC transporter FepB	*Escherichia coli*	P0AEL6	22	4.80E-05
VSAL_I0139	366	iron-siderophore ABC transporter FepD	*Escherichia coli*	P23876	40	1.70E-34
VSAL_I0140	341	iron-siderophore ABC transporter FecD	*Escherichia coli*	P15029	36	9.80E-24
VSAL_I0141	268	iron-siderophore ABC transporter FepC	*Escherichia coli*	P23878	44	2.30E-33
VSAL_I0142	104	transposase	*Pseudoalteromonas haloplanktis*	Q3IIG2	30	4.10E-06
VSAL_I0143	115	transposase	*Pseudoalteromonas haloplanktis*	Q3IIG1	58	1.40E-31
VSAL_I0144	495	transposase	*Shewanella sp.*	A1RLT8	57	1.10E-119
**GI-VS4**						
VSAL_I0872	58	hypothetical protein	*None*			
VSAL_I0873	243	hypothetical protein	*Moritella sp.*	A6FBR2	70	1.50E-77
VSAL_I0874	556	hypothetical protein	*Moritella sp.*	A6FBR3	75	1.20E-192
VSAL_I0875	524	glycine betaine transporter OpuD	*Bacillus subtilis*	P54417	33	3.40E-46
VSAL_I0876	329	membrane dipeptidase	*Vibrio sp.*	A7K3B0	92	3.70E-134
VSAL_I0877	487	aldehyde dehydrogenase	*Bacillus stearothermophilus*	P42329	38	2.00E-63
VSAL_I0878	127	endoribonuclease L-PSP	*Vibrio alginolyticus*	Q1V948	87	6.90E-45
VSAL_I0879	292	transcriptional regulator, LysR-family	*Vibrionales bacterium SWAT-3*	A5KWH7	87	1.50E-105
VSAL_I0880	72	hypothetical protein	*None*			
VSAL_I0881	258	putative exported protein	*Vibrio splendidus*	A3UNN5	59	1.10E-61
VSAL_I0882	251	putative exported protein	*Vibrio splendidus*	A3UNN4	47	2.80E-49
VSAL_I0883	863	putative exported protein	*Vibrio splendidus*	A3UNN3	55	2.90E-205
VSAL_I0884	137	putative exported protein	*Vibrio splendidus*	A3UNZ4	46	6.40E-19
**GI-VS5**						
VSAL_I1105	104	transposase	*Pseudoalteromonas haloplanktis*	Q3IIG2	30	4.10E-06
VSAL_I1106*	115	transposase	*Pseudoalteromonas haloplanktis*	Q3IIG1	58	1.40E-31
VSAL_I1107	495	transposase	*Shewanella sp.*	A1RLT8	57	1.10E-119
VSAL_I1108	880	chitodextrinase	*Vibrio furnissii*	P96156	77	0
VSAL_I1109	166	hypothetical protein	*Vibrio splendidus*	A3UR09	53	3.80E-28
VSAL_I1110	249	putative exported protein	*Aliivibrio fischeri*	Q5E663	78	4.60E-84
VSAL_I1111*	112	hypothetical protein	*Aliivibrio fischeri*	Q5E662	60	2.10E-20
VSAL_I1112	217	serine/threonine phosphoprotein phosphatase VasL	*Pseudomonas aeruginosa*	Q9I355	36	9.70E-18
VSAL_I1113*	1172	putative type VI secretion protein VasK	*Pseudomonas aeruginosa*	Q9I356	36	7.20E-150

VSAL_I1114	275	putative membrane protein VasF	*Pseudomonas aeruginosa*	Q9I357	50	1.30E-50
VSAL_I1115	440	putative type VI secretion protein VasE	*Pseudomonas aeruginosa*	Q9I358	47	1.10E-82
VSAL_I1116	162	putative type VI secretion protein VasD	*Pseudomonas aeruginosa*	Q9I359	40	3.00E-14
VSAL_I1117	453	putative type VI secretion protein VasC	*Pseudomonas aeruginosa*	Q9I360	32	1.20E-07
VSAL_I1118*	507	transcriptional regulatory protein VasH	*Pseudomonas aeruginosa*	Q9I362	40	3.00E-44
VSAL_I1119	872	chaperone ClpB VasG	*Y. enterocolitica*	Q9F746	42	9.00E-111
VSAL_I1120	309	putative type VI secretion protein VasB	*Pseudomonas aeruginosa*	Q9I364	48	2.80E-56
VSAL_I1121	583	putative type VI secretion protein VasA	*Vibrio cholerae*	Q9KN55	36	3.30E-74
VSAL_I1122	141	putative type VI secretion protein VasS	*Pseudomonas aeruginosa*	Q9I366	33	0.0001
VSAL_I1123	491	putative type VI secretion protein VasR	*Pseudomonas aeruginosa*	Q9I367	73	5.40E-150
VSAL_I1124	164	putative type VI secretion protein VasQ	*Pseudomonas aeruginosa*	Q9I368	61	2.90E-31
VSAL_I1125	513	putative type VI secretion protein VasJ	*Pseudomonas aeruginosa*	Q9I369	28	4.80E-40
**GI-VS6**						
VSAL_I1166	397	transposase	*Pasteurella piscicida*	A0PB90	52	9.70E-85
VSAL_I1167	186	putative type VI secretion protein VasU-1	*Vibrio splendidus*	A3USE8	64	7.80E-49
VSAL_I1168	98	putative type VI secretion protein VasV-1	*Vibrio splendidus*	A3USE9	89	6.80E-36
VSAL_I1169	461	putative type VI secretion protein VasJ-1	*Rhizobium leguminosarum*	Q93ED3	24	0.0033
VSAL_I1170	130	secreted hemolysin-coregulated protein Hcp-3	*Pseudomonas aeruginosa*	Q9I747	24	0.52
VSAL_I1171	169	putative type VI secretion protein VasQ-1	*Rhizobium leguminosarum*	Q93ED2	48	5.20E-21
VSAL_I1172	493	putative type VI secretion protein VasRA-1	*Rhizobium leguminosarum*	Q93ED1	51	1.20E-94
VSAL_I1173	453	putative type VI secretion protein VasRB-1	*Rhizobium leguminosarum*	Q93ED1	28	1.30E-19
VSAL_I1174	140	putative type VI secretion protein VasS-1	*None*			
VSAL_I1175	593	putative type VI secretion protein VasA-1	*Rhizobium leguminosarum*	Q93EC7	28	1.00E-44
VSAL_I1176	327	putative type VI secretion protein VasB-1	*Pseudomonas aeruginosa*	Q9I743	20	0.0056
VSAL_I1177	872	chaperone ClpB-1 VasG-1	*Yersinia enterocolitica*	Q9F746	42	4.70E-112
VSAL_I1178	52	putative type VI secretion protein VasV-1	*None*			
VSAL_I1179	314	putative type VI secretion protein VasC-1	*Pseudomonas aeruginosa*	Q9I751	30	4.9
VSAL_I1180	154	putative type VI secretion protein VasD-1	*Pseudomonas aeruginosa*	Q9I752	34	2.80E-07
VSAL_I1181	438	putative type VI secretion protein VasE-1	*Rhizobium leguminosarum*	Q93EC4	29	7.30E-22
VSAL_I1182	374	putative type VI secretion protein VasF-1	*Pseudomonas aeruginosa*	Q9I357	27	8.20E-12
VSAL_I1183*	1144	putative type VI secretion protein VasK-1	*Pseudomonas aeruginosa*	Q9I356	26	1.10E-56
VSAL_I1184	432	transposase	*Enterobacter agglomerans*	P71168	57	4.10E-104
VSAL_I1185	618	putative type VI secretion protein VasX-1	*Vibrio splendidus*	A3USC8	45	1.20E-29
**GI-VS7**						
VSAL_I1463	188	transposase	*Hahella chejuensis*	Q2SMC4	47	1.10E-34
VSAL_I1464	140	transposase	*Hahella chejuensis*	Q2SAQ2	84	2.70E-45
VSAL_I1465	71	hypothetical protein	*None*			

VSAL_I1466	318	transposase	*Pseudomonas putida*	Q0QXF0	39	2.10E-40
VSAL_I1467	140	hypothetical protein	*None*			
VSAL_I1468*	69	hypothetical protein	*Vibrio cholerae*	Q9KMD8	60	1.30E-10
VSAL_I1469	94	putative plasmid stabilisation system protein	*Aliivibrio fischeri*	A9I6D7	100	3.70E-35
VSAL_I1470	82	conserved hypothetical protein	*Aliivibrio fischeri*	A9I6D9	100	4.40E-31
VSAL_I1471	262	putative antibiotic resistance protein	*Rhizobium leguminosarum bv. trifolii*	P42729	39	1.90E-12
VSAL_I1472	150	NUDIX hydrolase	*Aliivibrio fischeri*	Q5E549	90	2.10E-60
VSAL_I1473	166	putative acetyltransferase	*Pseudomonas fluorescens*	Q3KAA5	50	1.50E-25
VSAL_I1474*	50	hypothetical protein	*Vibrio cholerae*	Q9KRR2	70	3.50E-13
VSAL_I1475	212	outer membrane protein	*None*			
VSAL_I1476	143	membrane protein	*None*			
VSAL_I1477	140	transposase	*Hahella chejuensis*	Q2SAQ2	84	2.70E-45
VSAL_I1478	188	transposase	*Hahella chejuensis*	Q2SMC4	47	1.10E-34
VSAL_I1479*	323	putative acyltransferase	*Bacillus sp.*	Q2B5J8	36	2.00E-28
VSAL_I1480*	81	putative membrane protein	*Pseudoalteromonas atlantica*	Q3CS45	44	8.30E-08
VSAL_I1481	135	putative lipoprotein	*Vibrio vulnificus*	Q7MD73	46	3.40E-17
VSAL_I1482	250	glucose 1-dehydrogenase	*Bacillus subtilis*	P12310	39	5.60E-25
VSAL_I1483	64	hypothetical protein	*Marinomonas sp.*	A3YG41	52	1.90E-11
VSAL_I1484	238	short chain dehydrogenase	*Chlamydia trachomatis*	P38004	34	1.40E-15
VSAL_I1485	232	conserved hypothetical protein	*Colwellia psychrerythraea*	Q47UH8	71	5.50E-75
VSAL_I1486	176	cold-shock protein	*Desulfovibrio vulgaris*	Q72AY2	38	1.10E-21
VSAL_I1487	117	conserved hypothetical protein	*Photobacterium profundum*	Q1ZAJ5	92	1.80E-49
VSAL_I1488	473	putative exported protein	*Vibrio harveyi*	A6AP36	43	4.80E-70
VSAL_I1489	130	hypothetical protein	*Aliivibrio fischeri*	A9ICX9	92	7.10E-45
VSAL_I1490	71	hypothetical protein	*None*			
VSAL_I1491	140	transposase	*Hahella chejuensis*	Q2SAQ2	84	2.70E-45
VSAL_I1492	188	transposase	*Hahella chejuensis*	Q2SMC4	47	1.10E-34
**GI-VS8**						
VSAL_I1620	140	transposase	*Hahella chejuensis*	Q2SAQ2	84	2.70E-45
VSAL_I1621	188	transposase	*Hahella chejuensis*	Q2SMC4	47	1.10E-34
VSAL_I1622	136	putative cell wall lytic enzyme	*Aliivibrio fischeri*	A9I2S4	60	2.00E-23
VSAL_I1623	247	putative sucrose-6F-phosphate phosphohydrolase	*Vibrio sp.*	A3Y1Q8	62	7.70E-65
VSAL_I1624	216	enhancing lycopene biosynthesis protein 2	*Escherichia coli*	P0ABU5	51	1.50E-37
VSAL_I1625	134	hypothetical protein	*Vibrio alginolyticus*	Q1VDS1	63	1.70E-34
VSAL_I1626	242	MltA-interacting protein MipA	*Vibrio splendidus*	A3V023	55	2.70E-54
VSAL_I1627	138	membrane protein	*Vibrio harveyi*	A6ALV0	60	5.90E-31
VSAL_I1628	320	putative radical SAM superfamily protein	*Nitrosococcus oceani*	Q3JDI7	63	1.60E-86
VSAL_I1629*	227	glycosyl transferase, family 2	*Nitrosococcus oceani*	Q3JDI9	54	2.70E-43

VSAL_I1630	204	hypothetical protein	*Nitrosococcus oceani*	Q3JDJ0	47	1.10E-40
VSAL_I1631*	436	sodium/solute symporter	*Hahella chejuensis*	Q2S7Q2	52	5.90E-65
VSAL_I1632	140	transposase	*Hahella chejuensis*	Q2SAQ2	84	2.70E-45
VSAL_I1633	188	transposase	*Hahella chejuensis*	Q2SMC4	47	1.10E-34
**GI-VS9**						
VSAL_I1787	199	GTP cyclohydrolase-2	*Photobacterium phosphoreum*	P51618	67	6.60E-48
VSAL_I1788	381	alcohol dehydrogenase 2	*Zymomonas mobilis*	P06758	62	1.70E-86
VSAL_I1789	238	conserved hypothetical protein	*Vibrio sp.*	A3XVM5	70	2.20E-73
VSAL_I1790	1072	restriction enzyme	*Pasteurella haemolytica*	P95512	28	2.80E-27
VSAL_I1791*	426	type I restriction-modification system specificity subunit	*Shewanella sp.*	Q36AA1	36	1.00E-35
VSAL_I1792	587	N-6 adenine-specific DNA methylase	*Salmonella enterica*	P72418	33	2.40E-42
VSAL_I1793	354	putative exported protein	*Aliivibrio fischeri*	A9ICK9	69	4.10E-101
VSAL_I1794*	44	sodium driven multidrug efflux pump	*Aliivibrio fischeri*	Q5E4U9	88	1.50E-12
**GI-VS10**						
VSAL_I3009	370	transposase	*Shewanella denitrificans*	Q12RN8	73	3.60E-115
VSAL_I3010	207	putative capsular polysaccharide biosynthesis protein NeuD	*Escherichia coli*	Q46674	68	5.20E-49
VSAL_I3011	346	putative sialic acid synthase NeuB	*Escherichia coli*	Q46675	69	6.70E-84
VSAL_I3012	417	N-acylneuraminate cytidylyltransferase NeuA	*Escherichia coli*	P13266	52	2.30E-77
VSAL_I3013	388	polysialic acid biosynthesis protein NeuC	*Escherichia coli*	Q47400	59	2.90E-84
VSAL_I3014	422	putative exopolysaccharide biosynthesis protein	*Aeromonas punctata*	Q7BR79	56	3.90E-79
VSAL_I3015	298	hypothetical protein	*Rhodopirellula baltica*	Q7ULW0	35	2.00E-18
VSAL_I3016	321	lipooligosaccharide sialyltransferase	*Escherichia coli*	Q93NP9	29	5.90E-14
VSAL_I3017	374	transposase	*Vibrio cholerae*	O51837	38	2.00E-48
VSAL_I3018	371	dTDP-glucose 4,6-dehydratase RmlB	*Vibrio cholerae*	Q842N5	85	7.10E-124
VSAL_I3019	295	glucose-1-phosphate thymidyl transferase RmlA	*Vibrio cholerae*	Q842R4	83	1.10E-93
VSAL_I3020*	293	dTDP-6-deoxy-l-mannose-dehydrogenase RmlD	*Vibrio cholerae*	Q842P5	68	2.00E-72
VSAL_I3021	184	dTDP-6-deoxy-D-xylo-4-hexulose-3,5-epimerase RmlC	*Vibrio cholerae*	Q842R2	84	7.60E-58
VSAL_I3024	292	putative glycosyl transferase	*Escherichia coli*	Q9X4C3	33	1.50E-10
VSAL_I3025	427	membrane protein	*None*			
VSAL_I3026	280	putative glycosyl transferase WbfP	*Shigella boydii*	Q6QQW8	30	2.90E-07
VSAL_I3027	267	putative glycosyl transferase WbfO	*Vibrio cholerae*	P74947	31	1.50E-07
VSAL_I3028	190	putative lipopolysaccharide biosynthesis O-acetyl transferase	*Escherichia coli*	P37750	35	2.00E-08
VSAL_I3029	378	putative NAD dependent epimerase/dehydratase WbfW	*Vibrio cholerae*	Q56626	26	9.00E-22
VSAL_I3030	333	putative lipopolysaccharide biosynthesis protein	*Aeromonas hydrophila*		29	1.80E-14
VSAL_I3031	320	O-antigen biosynthetic gene WbfT	*Vibrio cholerae*	Q56623	41	3.70E-33
VSAL_I3032	182	capsular polysaccharide synthesis enzyme WbfU	*Vibrio cholerae*	Q56624	88	1.90E-56
VSAL_I3033	644	polysaccharide biosynthesis protein WbfY	*Vibrio cholerae*	Q56627	71	6.80E-152

VSAL_I3032	182	galactosyl transferase, capsular polysaccharide synthesis enzyme	*Vibrio cholerae*	Q56624	88	1.90E-56
VSAL_I3033	644	polysaccharide biosynthesis protein, putative epimerase/dehydratase	*Vibrio cholerae*	Q56627	71	6.80E-152
VSAL_I3034*	294	transposase	*Shewanella putrefaciens*	A4Y9G9	67	1.90E-40
VSAL_I3035	397	transposase	*Pseudomonas syringae*	P24607	46	1.20E-78
VSAL_I3036*	306	methyl-accepting chemotaxis protein	*Shewanella oneidensis*	Q8EGZ8	37	2.70E-24
VSAL_I3037	340	transposase	*Photobacterium profundum*	Q6LH76	62	6.90E-79
VSAL_I3038	180	putative outer membrane protein	*Vibrio vulnificus*	Q7MQ48	32	1.50E-06
VSAL_I3039	397	transposase	*Pseudomonas syringae*	P24607	46	1.20E-78
VSAL_I3040	105	putative membrane protein				
VSAL_I3041	231	putative glycosyltransferase	*Edwardsiella ictaluri*	Q937Y0	38	1.80E-11
VSAL_I3042*	378	transposase	*Escherichia coli*	P76119	40	1.10E-58

Chr II

ϕVSA1

VSAL_II0534	327	site-specific recombinase IntIA	*Aliivibrio fischeri*	Q9ALI5	80	3.90E-99
VSAL_II0535*	380	transposase	*Alkaliphilus metalliredigens*	A6TTH3	36	2.20E-55
VSAL_II0536	251	membrane protein, putative phage gene	*Aliivibrio fischeri*	Q84B85	99	4.50E-83
VSAL_II0537	237	hypothetical protein, putative phage gene	*Streptococcus suis*	Q5H807	29	2.60E-06
VSAL_II0538	151	putative acetyltransferase, putative phage gene	*Vibrio cholerae*	Q9KMF8	53	2.80E-32
VSAL_II0539	131	hypothetical protein, putative phage gene	*Vibrio marinus*	Q9RA16	91	3.90E-29
VSAL_II0540	176	hypothetical protein, putative phage gene	*Vibrio marinus*	Q9RA15	96	3.40E-54
VSAL_II0541	321	hypothetical protein, putative phage gene	*None*			
VSAL_II0542	84	hypothetical protein, putative phage gene	*Vibrio sp.*	Q2F9T4	77	5.80E-26
VSAL_II0543	104	putative plasmid stabilisation system protein	*Vibrio vulnificus*	Q8CMA1	64	6.00E-23
VSAL_II0544	116	hypothetical protein, putative phage gene	*Vibrionales bacterium SWAT-3*	A5L7X1	78	4.30E-39
VSAL_II0545	156	hypothetical protein, putative phage gene	*Aliivibrio fischeri*	A9I6Q3	99	2.20E-57
VSAL_II0546	200	putative Fic-related phage protein	*Escherichia coli*	P20605	44	2.50E-30
VSAL_II0547	89	putative plasmid stabilisation system protein	*Vibrio cholerae*	A3GKT3	88	1.50E-32
VSAL_II0548	92	putative RelB antitoxin	*Vibrio cholerae*	Q9KML3	78	4.30E-27
VSAL_II0549	530	hypothetical protein, putative phage gene	*Shewanella baltica*	A5NCW0	53	1.40E-117
VSAL_II0550	123	hypothetical protein, putative phage gene	*None*			
VSAL_II0551	64	hypothetical protein, putative phage gene	*None*			
VSAL_II0552	57	hypothetical protein, putative phage gene	*None*			
VSAL_II0553	294	hypothetical protein, putative phage gene	*Burkholderia pseudomallei*	A8KFS6	55	1.60E-65
VSAL_II0554	83	antitoxin	*Escherichia coli*	P69346	45	1.20E-08
VSAL_II0555	86	toxin	*Escherichia coli*	P69348	65	7.10E-21
VSAL_II0556	60	hypothetical protein, putative phage gene	*None*			

VSAL_II0557	380	transposase	*Alkaliphilus metalliredigens*	A6TTH3	36	2.20E-55
VSAL_II0548	85	hypothetical protein, putative phage gene	*Vibrio cholerae*	A2PEQ3	87	8.10E-33
VSAL_II0559	174	hypothetical protein, putative phage gene	*Vibrio cholerae*	A5EZL3	66	2.30E-45
VSAL_II0560	134	hypothetical protein, putative phage gene	*Aliivibrio fischeri*	A9IET2	63	1.50E-38
VSAL_II0561	103	transposase	*Vibrio cholerae*	Q9K344	76	1.10E-23
VSAL_II0562	278	transposase	*Vibrio cholerae*	Q9K3D5	64	3.40E-81
VSAL_II0563*	78	transposase	*Geobacillus kaustophilus*	Q5L3N6	55	9.10E-10
VSAL_II0564	82	hypothetical protein, putative phage gene	*Aliivibrio fischeri*	Q5DZV7	89	6.00E-33
VSAL_II0565	442	putative ATP-dependent RNA helicase RhlE	*Escherichia coli*	P25888	48	1.60E-63
VSAL_II0566	175	hypothetical protein	*None*			
**ϕVSA2**						
VSAL_II0629*	36	hypothetical protein, putative phage gene	*None*			
VSAL_II0630	58	phage replication repressor RstR	*Vibrio cholerae*	Q9RQF7	78	2.30E-14
VSAL_II0631	415	phage replication and integration protein	*Vibrio parahaemolyticus*	Q9KGQ3	54	6.90E-74
VSAL_II0632*	78	hypothetical protein, putative phage integrase	*Bacteriophage VfO4K68*	Q9MBX6	52	0.00043
VSAL_II0633	58	phage replication repressor RstR	*Vibrio cholerae*	Q9RQF7	78	2.30E-14
VSAL_II0634	415	phage replication protein	*Vibrio parahaemolyticus*	Q9KGQ3	53	6.40E-74
VSAL_II0635*	87	hypothetical protein, putative phage integrase	*Vibrio phage Vf12*	O88148	58	3.70E-17
VSAL_II0636	103	putative translation initiation factor SUI1	*Aliivibrio fischeri*	Q5E003	88	6.30E-38
VSAL_II0637	116	hypothetical protein, putative phage gene	*Aliivibrio fischeri*	A9I735	77	8.70E-38
VSAL_II0638	305	putative phage recombinase	*Selenomonas ruminantium*	Q845Z5	29	1.10E-08
**ϕVSA3**						
VSAL_II0739	158	putative phage replication protein	*Bacteriophage P2*	Q06419	33	1.70E-05
VSAL_II0740	95	hypothetical protein, putative phage gene	*None*			
VSAL_II0741	77	putative phage zinc-binding transcriptional activator	*Bacteriophage L-413C*	Q858U4	41	6.80E-06
VSAL_II0742	221	putative HNH endonuclease	*Vibrio parahaemolyticus*	A6AYA5	50	7.90E-49
VSAL_II0743	414	hypothetical protein, putative phage gene	*None*			
VSAL_II0744	90	putative phage zinc-binding transcriptional activator	*Pseudomonas phage PsP3*	Q37973	35	0.27
VSAL_II0745	338	major capsid protein	*Bacteriophage HP1*	P51720	28	2.00E-20
**GI-VSA1**						
VSAL_II0117	397	transposase	*Pseudomonas syringae*	P24607	46	1.20E-78
VSAL_II0118	214	membrane protein	*Pasteurella piscicida*	Q6ZXD3	54	1.70E-17
VSAL_II0119	174	putative exported protein	*Vibrionales bacterium SWAT-3*	A5L0Q4	75	7.50E-51
VSAL_II0120	291	putative membrane protein	*Vibrio parahaemolyticus*	Q87JT9	80	3.30E-94
VSAL_II0121	105	putative exported protein	*Photobacterium profundum*	Q6LHZ8	83	2.60E-32

VSAL_II0122	187	putative membrane protein	*Vibrio harveyi*	A6AU28	48	1.50E-32
VSAL_II0123	307	ABC transporter, periplasmic solute binding protein	*Streptococcus sanguis*	P31304	27	8.10E-14
VSAL_II0124	254	ABC transporter, ATP binding protein	*Bacillus subtilis*	O34338	33	5.70E-17
VSAL_II0125	291	ABC transporter, membrane component	*Photobacterium sp.*	Q2BZL1	91	3.00E-93
VSAL_II0126	294	membrane protein	*Vibrio angustum*	Q1ZVE4	60	1.30E-70
VSAL_II0127	273	hypothetical protein, putative phage gene	*Streptococcus pyogenes*	Q8K7P3	32	3.90E-05
VSAL_II0128	108	hypothetical protein, putative phage gene	*None*			
**GI-VSA2**						
VSAL_II0202	397	transposase	*Pseudomonas syringae*	P24607	46	1.20E-78
VSAL_II0203	65	hypothetical protein	*None*			
VSAL_II0204	85	hypothetical protein	*None*			
VSAL_II0205	200	hypothetical protein	*None*			
VSAL_II0206	186	putative O-acetyltransferase	*Vibrio cholerae*	Q06962	43	2.00E-24
VSAL_II0207*	235	hypothetical protein	*None*			
VSAL_II0208	397	transposase	*Pseudomonas syringae*	P24607	46	1.20E-78
**GI-VSA3**						
VSAL_II0257*	491	transposase	*Shewanella sp.*	A1RLT8	57	2.10E-118
VSAL_II0258	115	transposase	*Pseudoalteromonas haloplanktis*	Q3IIG1	58	1.40E-31
VSAL_II0259	104	transposase	*Pseudoalteromonas haloplanktis*	Q3IIG2	30	4.10E-06
VSAL_II0260	293	transposase	*Shewanella denitrificans*	Q12IQ1	60	1.10E-75
VSAL_II0261	142	hypothetical protein	*None*			
VSAL_II0262	451	membrane protein	*Methylibium petroleiphilum*	A2SMZ1	32	2.50E-32
VSAL_II0263	278	putative short chain dehydrogenase	*Leptospira interrogans*	Q8EXT7	37	1.60E-30
VSAL_II0264	166	hypothetical protein	*None*			
VSAL_II0265	221	hypothetical protein	*None*			
VSAL_II0266	329	hypothetical protein	*None*			
VSAL_II0267	335	HTH-type transcriptional regulator, AraC family	*Mycobacterium tuberculosis*	Q06861	24	6.00E-17
VSAL_II0268	301	putative exported protein	*Aliivibrio fischeri*	Q5DZ88	74	1.50E-99
VSAL_II0269	1032	putative membrane protein	*Aliivibrio fischeri*	Q5DZ89	72	3.00E-187
VSAL_II0270	491	transposase	*Shewanella sp.*	A1RLT8	57	2.10E-118
VSAL_II0271	115	transposase	*Pseudoalteromonas haloplanktis*	Q3IIG1	58	1.40E-31
VSAL_II0272*	104	transposase	*Pseudoalteromonas haloplanktis*	Q3IIG2	30	4.10E-06
**GI-VSA4**						
VSAL_II0321	620	putative glycosyl transferase	*Escherichia coli*	P37653	27	1.90E-14

VSAL_II0322	439	putative membrane protein	*Photobacterium profundum*	Q1Z567	78	6.40E-150
VSAL_II0323	467	putative lipoprotein	*Photobacterium profundum*	Q1Z566	86	2.70E-184
VSAL_II0324	466	putative lipoprotein	*Photobacterium profundum*	Q1Z565	78	3.90E-164
VSAL_II0325	323	putative exported protein	*Photobacterium profundum*	Q1Z564	37	4.60E-38
VSAL_II0326	146	hypothetical protein	*Photobacterium profundum*	Q1Z571	50	2.70E-26
VSAL_II0327	381	putative nucleotidyl transferase	*Photobacterium profundum*	Q1Z572	70	2.70E-108
VSAL_II0328	97	putative anti-sigma F factor antagonist	*Photobacterium profundum*	Q1Z573	63	5.80E-22
VSAL_II0329	415	putative response regulator	*Photobacterium profundum*	Q1Z574	64	3.50E-107
VSAL_II0330	422	hypothetical protein	*Photobacterium profundum*	Q1Z563	66	7.40E-115
VSAL_II0331	335	putative exported protein	*Photobacterium profundum*	Q1Z575	64	7.60E-97
VSAL_II0332*	181	putative hemolysin-type calcium-binding protein	*Pasteurella haemolytica*	P55123	36	5.50E-05
VSAL_II0333	104	transposase	*Pseudoalteromonas haloplanktis*	Q3IIG2	30	4.10E-06
VSAL_II0334	115	transposase	*Pseudoalteromonas haloplanktis*	Q3IIG1	58	1.40E-31
VSAL_II0335	491	transposase	*Shewanella sp.*	A1RLT8	57	2.10E-118
**GI-VSA5**						
VSAL_II0362	296	hypothetical protein	*Aliivibrio fischeri*	A9I1Z7	61	3.00E-68
VSAL_II0363	382	putative response regulator	*Aliivibrio fischeri*	A9I1Z4	38	1.20E-45
VSAL_II0364	75	hypothetical protein	*None*			
VSAL_II0365	35	hypothetical protein	*None*			
VSAL_II0366	69	fimbrial protein, Flp/Fap pilin component Flp1	*Actinobacillus actinomycetemcomitans*	O66149	32	4.00E-03
VSAL_II0367	144	type IV leader peptidase TadV	*Actinobacillus actinomycetemcomitans*	Q7X0M3	33	5.70E-07
VSAL_II0368	260	putative Flp pilus assembly protein CpaB	*Aliivibrio fischeri*	Q5E107	60	1.50E-49
VSAL_II0369	445	type II/III secretion system protein RcpA	*Actinobacillus actinomycetemcomitans*	Q7X0M1	36	7.20E-27
VSAL_II0370	169	putative lipoprotein	*Aliivibrio fischeri*	Q5E105	56	1.70E-36
VSAL_II0371	405	type II secretion system protein TadZ	*Actinobacillus actinomycetemcomitans*	Q8GCZ9	25	2.00E-03
VSAL_II0372	428	type II secretion system protein TadA	*Actinobacillus actinomycetemcomitans*	Q9XC06	53	1.30E-77
VSAL_II0373	303	type II secretion system protein TadB	*Actinobacillus actinomycetemcomitans*	Q9XC05	37	5.30E-28
VSAL_II0374	292	type II secretion system protein TadC	*Actinobacillus actinomycetemcomitans*	Q9S4A8	33	7.00E-23
VSAL_II0375	247	putative secretion system protein TadD	*Actinobacillus actinomycetemcomitans*	Q9S4A7	30	7.10E-13
VSAL_II0376	162	membrane associated secretion system protein TadE	*Actinobacillus actinomycetemcomitans*	Q7X0L4	24	7.50E-01
VSAL_II0377	179	membrane associated secretion system protein TadF	*Actinobacillus actinomycetemcomitans*	Q9S4A5	31	7.20E-08
VSAL_II0378	422	membrane associated secretion system protein TadG	*Actinobacillus actinomycetemcomitans*	Q9S4A4	21	4.60E-04
VSAL_II0379*	212	outer membrane protein, OmpA family	*Aliivibrio fischeri*	Q5E7H8	36	1.10E-22
VSAL_II0380	363	membrane protein	*Aliivibrio fischeri*	A8T8Z7	47	1.00E-58
**GI-VSA6**						
VSAL_II0986	725	hypothetical protein	*None*			

VSAL_II0986	506	hypothetical protein	*None*			
VSAL_II0986	225	hypothetical protein	*None*			
VSAL_II0986*	314	putative exported protein	*Vibrio angustum*	Q1ZM92	39	1.40E-28
VSAL_II0986	397	transposase	*Pseudomonas syringae*	P24607	46	1.20E-78

*Indicate pseudogenes and gene remnants
