# Supplementary material for: The genome sequence of the fish pathogen Aliivibrio salmonicida strain LFI1238 shows extensive evidence of gene decay
Source: BMC Genomics. 2008 Dec 19;9:616. doi: 10.1186/1471-2164-9-616 (PMC2627896; doi:10.1186/1471-2164-9-616)

Additional file 6. A: The two duplicated region in *A. salmonicida*; **B:** Agarose gel electrophoresis of PCR products confirming the presence of the duplication in all tested *A. salmonicida* isolates.

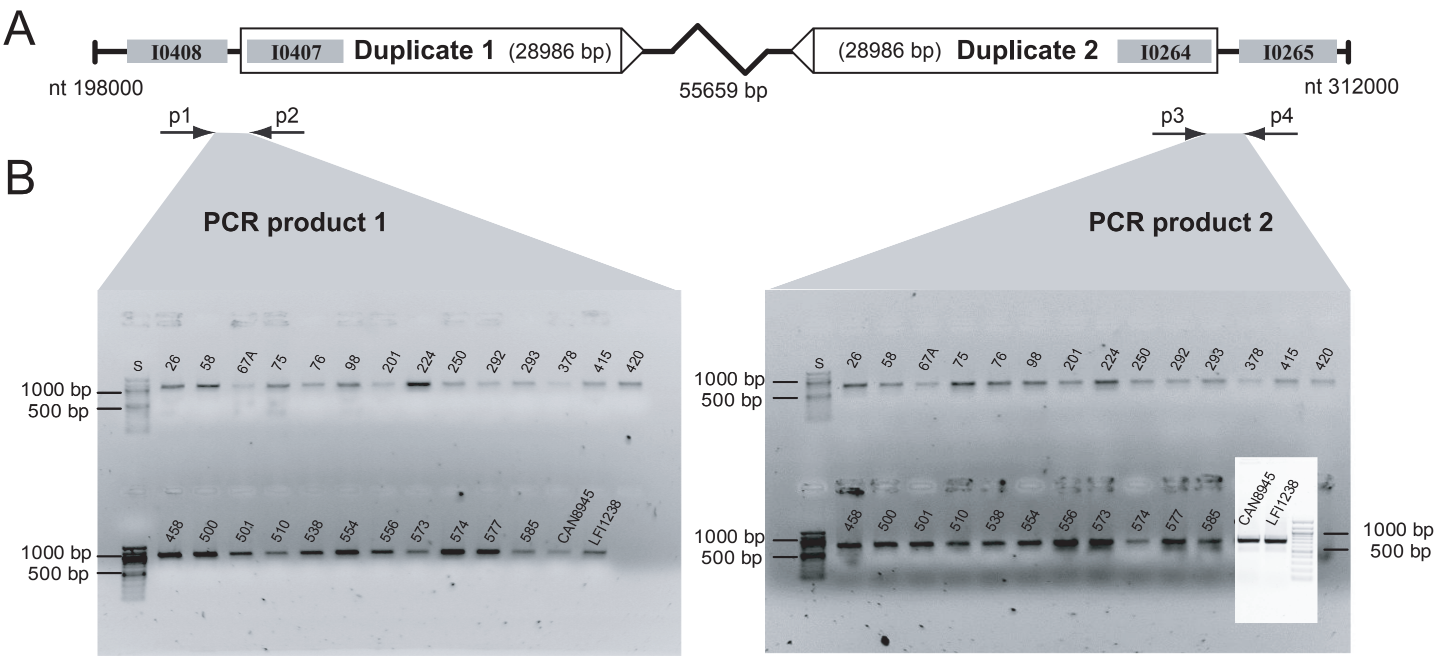

Supplement: Additional file 7 — A: The two duplicated regions in A. salmonicida LFI1238; B: Agarose gel electrophoresis of PCR products confirming the presence of the duplication in all tested A. salmonicida isolates. The first gene of each duplicate and the flanking genes are indicated as grey boxes (not drawn to scale). B) Agarose gel electrophoresis of duplication products VSAL_I0408-I0407 (PCR product 1) and VSAL_I0264-I0265 (PCR product 2), and the corresponding regions of different A. salmonicida isolates derived by PCR from genomic DNA. Primers are designed from LFI1238 and are indicated by arrows (p1 5'-CGACATGATCGTGTTTTGCT-3', p2 and p3 5'-GGAAAATAGCATCAATTGTA-3', p4 5'-CCATTGTAGAGGTGAGTTTA-3'). The A. salmonicida isolates were obtained from various outbreaks of cold water vibriosis from salmon and cod. The isolate numbers are indicated above each lane. S, 100 bp DNA ladder. [file 1471-2164-9-616-S7.pdf]
