# Supplementary material for: The genome sequence of the fish pathogen Aliivibrio salmonicida strain LFI1238 shows extensive evidence of gene decay
Source: BMC Genomics. 2008 Dec 19;9:616. doi: 10.1186/1471-2164-9-616 (PMC2627896; doi:10.1186/1471-2164-9-616)
Supplement: Additional file 8 — Functional and putative non-functional A. salmonicida genes involved in key steps of the chitinolytic cascade, together with orthologues from other sequenced Vibrionaceae. The data provided in this table lists all identified A. salmonicida CDSs that are known to be involved in the chitinolytic cascade in other Vibrionaceae. Disrupted CDSs and pseudogenes are labelled in the table. [file 1471-2164-9-616-S8.pdf]

Additional file 8. Functional and putative non-functional *A. salmonicida* genes involved in key steps of the chitinolytic cascade, together with orthologues from other sequenced *Vibrionaceae*.

A. salmonicida **LFI1238**	**Product**	***V. cholerae*** **N16961**	***V. parahaemolyticus*** **RIMD 2210633**	***V. vulnificus*** **YJ016**	***V. vulnificus*** **CMCP6**	***V. harveyi*** **ATCC BAA-1116**	***V. cholerae*** **O395**	***A fischeri*** **ES114**
**Sensing/attachment**								
VSAL_I2601[#]	Methyl-accepting chemotaxis protein[1)]	VC0449	VP2629	VV2887	VV1_1511	VIBHAR_07022	VC0395_A2871	VF2161
VSAL_II0134	Chitin binding protein[1)]	VCA0811*	VPA1598	VVA0551	VV2_0044	VIBHAR_04739	VC0395_0423	VFA0143
**Extracellular degradation**								
VSAL_I0757	Endochitinase[2)]	VC1952*	VP2338	VV2578	VV1_1833	VIBHAR_03258<br>VIBHAR_00548	VC0395_A1540	VF0655
VSAL_I0902/I0763[#]	Chitinase A[3)]					VIBHAR_05899		
VSAL_I1942[#]	Chitinase A[2)]	VCA0027*	VPA0055	VVA0044	VV2_1217	VIBHAR_06955	VC0395_0106	VF1598
VSAL_I1414[#]	Putative chitinase [3)]	VC0769*	VP0619	VV0777	VV1_0417	VIBHAR_01081	VC0395_A0298	VF1390
VSAL_II0217	Chitinase B[2)]	VCA0140*	VPA0092	VVA0086	VV2_1258	VIBHAR_02190	VC0395_1137	VFA0013
**Outer membrane transport**								
VSAL_I2352[#]	Chitoporin[4)]	VC0972*	VP0760	VV0946	VV1_0238	VIBHAR_01269	VC0395_A0493	VF1889
**Periplasmic degradation**								
VSAL_I1108[#]	Chitodextrinase[5)]	VCA0700*	VPA0832	VVA0720	VV2_0213	VIBHAR_05945	VC0395_0638	VF0986
VSAL_I2583	Beta-N-acetylglucosaminidase[6)]	VC0613*	VP2486	VV2741	VV1_1666	VIBHAR_03430	VC0395_A0142	VF2146
VSAL_II0050	N,N'-diacetylchitobiase[7)]	VC2217*	VP0755	VV0942	VV1_0241	VIBHAR_01265	VC0395_A1809	VFA1010
VSAL_II0575	Beta-N-acetylhexosaminidase[6)]	VC0692	VP0545	VV0700	VV1_0496	VIBHAR_00991	VC0395_A0223	VFA0493
**Inner membrane transport**								
VSAL_I0703	PTS system, cellobiose-specific IIC component[1)]	VC1282	VP2636	VV2900	VV1_1483	VIBHAR_03624	VC0395_A0901	VF0603
VSAL_I0704	PTS system, cellobiose-specific IIB component[1)]	VC1281	VP2637	VV2901	VV1_1482	VIBHAR_03625	VC0395_A0900	VF0604
VSAL_I0705	PTS system, 6-phospho-beta-glucosidase[1)]	VC1284	VP2634	VV2898	VV1_1485	VIBHAR_03622	VC0395_A0903	VF0605
VSAL_I0706	PTS system, putative glucokinase							VF0606
VSAL_I0707	PTS system, cellobiose-specific IIA component[1)]	VC1283	VP2635	VV2899	VV1_1484	VIBHAR_03623	VC0395_A0902	VF0607
VSAL_I0708	PTS system, transcriptional regulator[1)]	VC1286	VP2632	VV2896	VV1_1487	VIBHAR_03620	VC0395_A0905	VF0608
VSAL_I0831	PTS system, N-acetylglucosamine and glucose permease[6)]	VC0995	VP0831	VV1012	VV1_0179	VIBHAR_01336	VC0395_A0516	VF0808
VSAL_I2577	ABC-type [(GlcNAc)$_2$] transporter, permease protein[6)]	VC0619*	VP2480	VV2735	VV1_1672	VIBHAR_03424	VC0395_A0148	VF2140
VSAL_I2578	ABC-type [(GlcNAc)$_2$] transporter, permease protein[6)]	VC0618*	VP2481	VV2736	VV1_1671	VIBHAR_03425	VC0395_A0147	VF2141
VSAL_I2579	ABC-type [(GlcNAc)$_2$] transporter, ATP-binding protein[6)]	VC0617*	VP2482	VV2737	VV1_1670	VIBHAR_03426	VC0395_A0146	VF2142
VSAL_I2580	ABC-type [(GlcNAc)$_2$] transporter, ATP-binding protein[6)]	VC0616*	VP2483	VV2738	VV1_1669	VIBHAR_03427	VC0395_A0145	VF2143
**Intracellular degradation**								
VSAL_I0830	N-acetylglucosamine-6-phosphate deacetylase[8)]	VC0994	VP0829	VV1011	VV1_0180	VIBHAR_01335	VC0395_A0515	VF0807

VSAL_I2581	Chitobiase (exoglucosidase)[6]	VC0615*	VP2484	VV2739	VV1_1668	VIBHAR_03428	VC0395_A0144	VF2144
VSAL_I2582	Glucosamine kinase[6]	VC0614*	VP2485	VV2740	VV1_1667	VIBHAR_03429	VC0395_A0143	VF2145
VSAL_I2584	Chitobiose phosphorylase[6]	VC0612*	VP2487	VV2742	VV1_1665	VIBHAR_03431	VC0395_A0141	VF2147
VSAL_I2585	Phosphoglucomutase[6]	VC0611*	VP2488	VV2743	VV1_1664	VIBHAR_03432	VC0395_A0140	VF2148
VSAL_I2812	Glucosamine-6-phosphate deaminase[9]	VCA1025	VPA0038	VVA0028	VV2_1200	VIBHAR_07002	VC0395_0216	VF2357
**Regulation**								
VSAL_I2575	Chitin degradation sensor protein[6]	VC0622	VP2478	VV2733	VV1_1674	VIBHAR_03422	VC0395_A0150	VF2138
VSAL_I2576[#]	[(GlcNAc)n] binding protein[6]	VC0620*	VP2479	VV2734	VV1_1673	VIBHAR_03423	VC0395_A0149	VF2139

[#]Degenerated A. salmonicida genes; [*]ChiS regulated in V. cholerae; [1)]Meibom et al. 2003, [2)]Orikoshi et al. 2005; [3)]Svitvil and Kirchman 1998; [4)]Keyhani et al. 2000;
[5)]Keyhani and Roseman 1996b; [6)]Li and Roseman 2004; [7)]Soto-Gil and Zyskind 1989; [8)]Yamano et al. 1997 and [9)]Rogers et al. 1988
