# Supplementary material for: The genome sequence of the fish pathogen Aliivibrio salmonicida strain LFI1238 shows extensive evidence of gene decay
Source: BMC Genomics. 2008 Dec 19;9:616. doi: 10.1186/1471-2164-9-616 (PMC2627896; doi:10.1186/1471-2164-9-616)
Supplement: Additional file 9 — Growth of different Vibrionaceae isolates on α-chitin and GlcNAc. The data provided shows that the A. salmonicida isolates investigated in this study are not able to utilise α-chitin or GlcNAc. [file 1471-2164-9-616-S9.pdf]

Additional file 9. Growth of different *Vibrionaceae* isolates on α-chitin and GlcNAc

**Isolate**	**α-chitin**	**GlcNAc**
A. salmonicida		
LFI1238	-	-
88	-	-
378	-	-
224	-	-
415	-	-
67A	-	-
A. wodanis		
ATCC BAA-104	-	(+)
04/17347	-	-
06/139A	+	+
02/569	-	+
V. splendidus		
ATCC 33125	+	+
03/122	+	+
02/066	(+)	-
00/860	+	+

+, growth; (+), weak growth; -, no growth
A. salmonicida and *A. wodanis* grown at 12°C,
V. splendidus grown at room temperature.
