# Supplementary material for: The genome sequence of the fish pathogen Aliivibrio salmonicida strain LFI1238 shows extensive evidence of gene decay
Source: BMC Genomics. 2008 Dec 19;9:616. doi: 10.1186/1471-2164-9-616 (PMC2627896; doi:10.1186/1471-2164-9-616)
Supplement: Additional file 10 — Predicted CDSs of A. salmonicida with potential roles in pathogenicity. The data in this table gives a comprehensive list of all CDSs with possible functions that may be associated with mediating cold-water vibriosis in fish. [file 1471-2164-9-616-S10.pdf]

Additional file 10. Predicted coding sequences of *A. salmonicida* with potential roles in pathogenicity.

Class	Putative function	1)	2)Gene ID	Size	Predicted product	3)Similar to	Acc. No.	Identity (%)	E-value	PubMed id
**Adherence/motility**										
	**Polar flagella**									
			VSAL_I0936	254	Sodium-driven polar flagellar protein (*motA*)	*V. parahaemolyticus*	Q9Z6H0	79	7.40E-67	10850984
			VSAL_I0937	309	Sodium-driven polar flagellar protein (*motB*)	*V. parahaemolyticus*	Q9Z6G9	75	2.30E-86	10850984
			VSAL_I1863	292	Sodium-driven polar flagellar protein (*motY*)	*V. parahaemolyticus*	P46233	66	1.50E-75	10850984
			VSAL_I2282	153	Putative membrane protein	*V. parahaemolyticus*	Q87MK7	49	8.20E-19	No
			VSAL_I2283	164	Chemotaxis protein (*cheW*)	*V. parahaemolyticus*	Q9LB08	88	5.60E-49	10850984
			VSAL_I2284	363	CheW-like protein	*V. parahaemolyticus*	Q87MK6	50	1.40E-56	No
			VSAL_I2285	255	Putative chromosome segregation protein	*V. parahaemolyticus*	Q9LB10	75	6.60E-68	No
			VSAL_I2286	380	Chemotaxis response regulator (*cheB*)	*V. parahaemolyticus*	Q87MK5	78	4.30E-61	10850984
			VSAL_I2287	723	Chemotaxis protein (*cheA*)	*V. parahaemolyticus*	Q9LB12	72	1.20E-140	10850984
			VSAL_I2288	239	Chemotaxis protein (*cheZ*)	*V. parahaemolyticus*	Q9LB13	67	5.80E-51	10850984
			VSAL_I2289	130	Chemotaxis protein (*cheY*)	*V. parahaemolyticus*	Q9LB14	95	5.10E-40	10850984
			VSAL_I2290	243	RNA polymerase sigma factor (*fliA*)	*V. parahaemolyticus*	Q9LB15	72	7.50E-62	10850984
			VSAL_I2291	296	Flagellar biosynthesis protein (*flhG*)	*V. parahaemolyticus*	Q9LB16	78	2.30E-78	10850984
			VSAL_I2292	484	Flagellar biosynthesis protein (*flhF*)	*V. parahaemolyticus*	Q9LB17	64	1.60E-86	10850984
			VSAL_I2293	697	Polar flagellar assembly protein (*flhA*)	*V. parahaemolyticus*	Q9Z6F4	83	5.90E-206	10850984
			VSAL_I2295	376	Polar flagellar assembly protein (*flhB*)	*V. parahaemolyticus*	Q9Z6F5	75	1.80E-103	10850984
			VSAL_I2296	260	Polar flagellar assembly protein (*fliR*)	*V. parahaemolyticus*	Q9Z6F6	80	1.30E-67	10850984
			VSAL_I2297	89	Polar flagellar assembly protein (*fliQ*)	*V. parahaemolyticus*	Q9Z6F7	85	1.70E-25	10850984
			VSAL_I2298	289	Polar flagellar assembly protein (*fliP*)	*V. parahaemolyticus*	Q9Z6F8	73	3.60E-68	10850984
			VSAL_I2299	129	Polar flagellar assembly protein (*fliO*)	*V. parahaemolyticus*	Q9Z6F9	58	6.50E-20	10850984
			VSAL_I2300	127	Polar flagellar switch protein (*fliN*)	*V. parahaemolyticus*	Q9Z6G0	78	1.30E-30	10850984
			VSAL_I2301	343	Polar flagellar switch protein (*fliM*)	*V. parahaemolyticus*	Q9Z6G1	85	9.10E-108	10850984
			VSAL_I2302	161	Polar flagellar protein (*fliL*)	*V. parahaemolyticus*	Q9Z6G2	56	1.70E-30	10850984
			VSAL_I2303	407	Polar flagellar hook-length control protein (*fliK*)	*V. parahaemolyticus*	Q9Z6G3	51	2.70E-22	10850984
			VSAL_I2304	145	Polar flagellar assembly protein (*fliJ*)	*V. parahaemolyticus*	Q9Z6G4	65	4.20E-34	10850984
			VSAL_I2305	439	Polar flagellum-specific ATP synthase (*fliI*)	*V. parahaemolyticus*	Q9Z6G5	86	2.60E-133	10850984
			VSAL_I2306	265	Polar flagellar assembly protein (*fliH*)	*V. parahaemolyticus*	Q9Z6G6	52	2.50E-44	10850984
			VSAL_I2307	350	Polar flagellar motor switch protein (*fliG*)	*V. parahaemolyticus*	Q9Z6G7	82	6.20E-98	10850984
			VSAL_I2308*	577	Flagellar M-ring protein (*fliF*)	*V. parahaemolyticus*	Q9Z6G8	70	3.10E-156	10850984
			VSAL_I2309	103	Flagellar hook-basal body complex protein (*fliE*)	*V. parahaemolyticus*	Q9LB18	71	1.80E-24	10850984
			VSAL_I2310	478	Sigma-54 dependent response regulator (*flaM*)	*V. parahaemolyticus*	Q9LB19	80	1.00E-112	10850984
			VSAL_I2311	347	Histidine kinase (*flaL*)	*V. parahaemolyticus*	Q56709	78	2.50E-96	10850984
			VSAL_I2312	493	Polar flagellar protein (*flaK*)	*V. parahaemolyticus*	Q56708	68	6.00E-128	10850984
			VSAL_I2313	138	Polar flagellar protein (*flaJ*)	*V. parahaemolyticus*	Q56707	84	1.50E-40	10850984
			VSAL_I2314	96	Polar flagellar protein (*flaI*)	*V. parahaemolyticus*	Q56706	27	9.40E-05	10850984
			VSAL_I2315	646	Polar flagellar hook-associated protein 2 (*fliDP/flaH*)	*V. parahaemolyticus*	Q56705	45	3.40E-46	10850984
			VSAL_I2316*	138	Polar flagellar protein (*flaG*)	*V. parahaemolyticus*	Q56704	38	1.00E-08	10850984

	VSAL_I2317	378	Flagellin subunit E (*flaE*)	*A. fischeri*	Q5E3N9	93	3.40E-111	15205434
	VSAL_I2318	375	Flagellin subunit D (*flaD*)	*A. fischeri*	Q6R4P9	93	8.10E-116	15205434
	VSAL_I2319	402	Flagellin subunit C (*flaC*)	*A. fischeri*	Q6R4Q0	94	1.00E-124	15205434
	VSAL_I2325	377	Flagellin subunit C (*flaB*)	*V. parahaemolyticus*	O87081	68	2.80E-89	10850984
	VSAL_I2326	125	Putative exported protein	None				
	VSAL_I2327	379	Flagellin subunit C (*flaA*)	*V. parahaemolyticus*	Q56712	70	4.30E-87	10850984
	VSAL_I2328	397	Flagellar hook-associated protein type 3 (*flgL*)	*V. parahaemolyticus*	Q56711	61	2.50E-87	10850984
	VSAL_I2329	626	Flagellar hook-associated protein type 3 (*flgM*)	*V. parahaemolyticus*	Q87RK5	60	4.80E-102	10850984
	VSAL_I2330	332	Peptidoglycan hydrolase (*flgJ*)	*V. parahaemolyticus*	Q9X9J3	54	5.60E-38	10850984
	VSAL_I2331	373	Flagellar P-ring protein 2 precursor (*flgI*)	*V. parahaemolyticus*	Q9X9J4	84	2.20E-105	10850984
	VSAL_I2332	261	Flagellar L-ring protein 1 precursor (*flgH*)	*V. parahaemolyticus*	Q9X9J5	72	6.00E-73	10850984
	VSAL_I2333	262	Flagellar basal-body rod protein (*flgG*)	*V. parahaemolyticus*	Q9X9J6	85	4.90E-83	10850984
	VSAL_I2334	241	Flagellar basal-body rod protein (*flgF*)	*V. parahaemolyticus*	Q9X9J7	78	1.10E-67	10850984
	VSAL_I2335	432	Flagellar hook protein (*flgE*)	*V. parahaemolyticus*	Q9X9J8	67	6.30E-115	10850984
	VSAL_I2336	236	Flagellar basal-body rod protein (*flgD*)	*V. parahaemolyticus*	Q9X9J9	77	1.20E-65	10850984
	VSAL_I2337	138	Flagellar basal-body rod protein (*flgC*)	*V. parahaemolyticus*	Q9X9K0	84	2.80E-40	10850984
	VSAL_I2338	131	Flagellar basal-body rod protein (*flgB*)	*V. parahaemolyticus*	Q9X9K1	79	4.10E-39	10850984
	VSAL_I2339	275	Chemotaxis protein (*cheR*)	*V. parahaemolyticus*	Q9X9K2	84	8.60E-91	10850984
	VSAL_I2340	306	Chemotaxis protein (*cheV*)	*V. parahaemolyticus*	Q9X9K3	81	2.50E-79	10850984
	VSAL_I2341	248	Polar flagellar basal-body P-ring formation protein (*flgA*)	*V. parahaemolyticus*	Q9X9K4	43	5.40E-40	10850984
	VSAL_I2342	103	Regulator of flagellin synthesis (*flgM*)	*V. parahaemolyticus*	Q9X9K5	51	9.40E-15	10850984
	VSAL_I2343	142	Polar flagellar (*flgN*)	*V. parahaemolyticus*	Q87RK8	53	8.40E-22	10850984
	VSAL_I2517	385	Flagellin subunit D (*flaF*)	*A. fischeri*	Q5E322	91	2.30E-113	No
	VSAL_I2771	210	Sodium-driven polar flagellar protein (*motX*)	*V. parahaemolyticus*	P40608	75	1.40E-60	10850984
	VSAL_I2897	136	Putative flagellar basal body-associated protein (*fliL*)	*A. fischeri*	Q5E205	86	8.60E-48	No
	**VSAL_II0231**	313	Chemotaxis protein (*cheV*)	*A. hydrophila*	Q8GLQ0	52	4.20E-51	12732473
**Chemotaxis**								
	VSAL_I0799	624	Methyl-accepting chemotaxis protein	*A. fischeri*	Q5E6S4	92	1.80E-196	No
	VSAL_I0920	662	Nitrate and nitrite sensing methyl-accepting chemotaxis protein	*P. aeruginosa*	Q9HW91	25	1.10E-25	No
	VSAL_I1294	663	Methyl-accepting chemotaxis protein	*A. fischeri*	Q5E5R8	86	4.10E-186	No
	VSAL_I1636	547	Methyl-accepting chemotaxis protein	*V. cholerae*	P29486	34	1.10E-28	8005659
	VSAL_I1951	720	Methyl-accepting chemotaxis protein	*P. profundum*	Q1Z156	48	4.00E-116	No
	VSAL_I2075	362	Methyl-accepting chemotaxis protein	*A. fischeri*	Q5E6H9	85	6.10E-102	No
	VSAL_I2130	546	Methyl-accepting chemotaxis protein	*P. aeruginosa*	O32443	39	1.00E-36	9353923
	VSAL_I2193	697	Methyl-accepting chemotaxis protein	*A. fischeri*	Q5E3W2	82	1.50E-199	No
	VSAL_I2678	533	Methyl-accepting chemotaxis protein	*A. fischeri*	Q5E2L5	83	2.80E-144	No
	**VSAL_II0170**	543	Methyl-accepting chemotaxis protein	*V. cholerae*	P29486	37	1.30E-31	8005659
	**VSAL_II0498**	302	Chemotaxis protein, CheW-like	*A. fischeri*	Q5DZM8	89	4.90E-89	No
	**VSAL_II0675**	542	Methyl-accepting chemotaxis protein	*A. fischeri*	Q5E095	81	1.20E-144	No
	**VSAL_II0712**	546	Methyl-accepting chemotaxis protein	*A. fischeri*	Q5E0C8	77	5.50E-130	No
	**VSAL_II0756**	558	Methyl-accepting chemotaxis protein	*A. fischeri*	Q5E0Q1	89	6.20E-165	No
	**VSAL_II0771**	661	Methyl-accepting chemotaxis protein	*A. fischeri*	Q5E0S6	78	4.40E-163	No
	**VSAL_II1022**	628	Methyl-accepting chemotaxis protein	*V. vulnificus*	Q7MDS3	77	1.00E-163	No

**MSHA Type IV pilus**								
	VSAL_I0466	487	Type IV pilus, mannose-sensitive hemagglutinin D (*mshI*)	*V. cholerae*	Q56670	39	6.50E-52	9973335
	VSAL_I0467	212	Type IV pilus, mannose-sensitive hemagglutinin D (*mshJ*)	*V. cholerae*	Q56671	43	4.70E-30	9973335
	VSAL_I0468	113	Type IV pilus, mannose-sensitive hemagglutinin D (*mshK*)	*V. cholerae*	Q56672	39	2.20E-07	9973335
	VSAL_I0469	546	Type IV pilus, mannose-sensitive hemagglutinin D (*mshD*)	*V. cholerae*	Q56673	66	7.10E-37	9973335
	VSAL_I0470	282	Type IV pilus, mannose-sensitive hemagglutinin D (*mshM*)	*V. cholerae*	Q56674	59	2.30E-57	9973335
	VSAL_I0471	357	Type IV pilus, mannose-sensitive hemagglutinin D (*mshN*)	*V. cholerae*	Q56675	36	4.20E-27	9973335
	VSAL_I0472*	576	Type IV pilus, mannose-sensitive hemagglutinin E (*mshE*)	*V. cholerae*	Q56676	77	4.10E-142	9973335
	VSAL_I0473	408	Type IV pilus, mannose-sensitive hemagglutinin (*mshG*)	*V. cholerae*	Q56612	68	5.40E-94	9973335
	VSAL_I0474	150	Type IV pilus, mannose-sensitive hemagglutinin (*mshF*)	*V. cholerae*	Q56650	29	4.50E-08	9973335
	VSAL_I0475	189	Type IV pilus, mannose-sensitive hemagglutinin (*mshB*)	*V. cholerae*	Q60073	55	1.20E-30	9973335
	VSAL_I0476	157	Type IV pilus, mannose-sensitive hemagglutinin (*mshA*)	*V. cholerae*	Q6BEA1	56	1.70E-26	9973335
	VSAL_I0477	156	Type IV pilus, mannose-sensitive hemagglutinin (*mshA*)	*V. cholerae*	Q6BEA1	60	9.50E-25	9973335
	VSAL_I0478	204	Type IV pilus, mannose-sensitive hemagglutinin (*mshC*)	*V. cholerae*	Q56651	35	1.20E-03	9973335
	VSAL_I0479	197	Type IV pilus, mannose-sensitive hemagglutinin (*mshD*)	*V. cholerae*	Q79AF3	37	9.40E-08	9973335
	VSAL_I0480	251	Exported protein, putative type IV prepilin (*mshO*)	*V. cholerae*	Q9ZA95	29	2.00E-14	9973335
	VSAL_I0481	144	Exported protein (*mshP*)	*V. cholerae*	Q9ZA94	29	1.70E-02	9973335
	VSAL_I0482*	1430	Hypothetical protein (*mshQ*)	*V. cholerae*	Q9ZA93	22	1.00E-07	9973335
**Type IV pilus assembly proteins**								
	VSAL_I0544	345	Type II/IV secretion system protein (*pilT*)	*P. aeruginosa*	P24559	68	1.10E-77	1676385
	VSAL_I0545	368	Type II/IV secretion system protein (*pilU*)	*P. aeruginosa*	Q51532	58	9.80E-70	7854122
	VSAL_I0671	143	Putative type IV fimbrial biogenesis protein (*pilE*)	*P. aeruginosa*	Q60148	32	4.60E-08	7854130
	VSAL_I0727	296	Putative type IV fimbrial biogenesis protein (*pilF*)	*P. aeruginosa*	Q51526	35	8.70E-17	8973346
	VSAL_I2629	138	Type IV pilus subunit (*pilA*)	*V. cholerae*	Q9X4G7	41	8.40E-10	10024587and 15731039
	VSAL_I2630	563	Type IV pilin assembly protein (*pilB*)	*V. cholerae*	Q9X4G8	68	9.00E-130	10024587and 15731039
	VSAL_I2631*	412	Type IV pilin assembly protein (*pilC*)	*V. cholerae*	Q9X4G9	66	3.00E-97	10024587and 15731039
	VSAL_I2632	295	Type IV prepilin peptidase (*pilD*)	*V. cholerae*	O68839	65	3.20E-70	10024587and 15731039
	VSAL_I2716	332	Fimbrial assembly protein (*pilM*)	*P. aeruginosa*	Q51351	22	6.60E-08	7565110
	VSAL_I2717	189	Fimbrial assembly protein (*pilN*)	*P. aeruginosa*	Q51352	27	1.00E-09	7565110
	VSAL_I2718	193	Fimbrial assembly protein (*pilO*)	*P. aeruginosa*	Q51353	34	1.80E-15	7565110
	VSAL_I2719	172	Fimbrial assembly protein (*pilP*)	*P. aeruginosa*	Q51354	27	9.40E-07	7565110
	VSAL_I2720*	573	Fimbrial assembly protein (*pilQ*)	*P. aeruginosa*	P34750	40	4.80E-36	7565110
**F-pilus assembly protein**								
	VSAL_p840_01	56	Conjugative transfer protein (*traY*)	*E. coli*	P05835	54	1.20E-03	2836369
	VSAL_p840_02	93	Conjugative transfer protein (*traA*)	*E. coli*	P14496	36	1.10E-01	6090426
	VSAL_p840_03	100	Conjugative transfer protein (*traL*)	*E. coli*	P08321	24	4.40E+00	8655498
	VSAL_p840_04	186	Conjugative transfer protein (*traE*)	*E. coli*	Q46998	30	4.60E-07	8655498
	VSAL_p840_05	251	Conjugative transfer protein (*traK*)	*E. coli*	Q47003	23	2.80E-04	8655498
pVS840	*VSAL_p840_06*	476	Conjugative transfer protein (*traB*)	*E. coli*	P41067	29	1.30E-23	8655498
	VSAL_p840_07	133	Putative conjugative transfer protein (*traV*)	*V. vulnificus*	Q8D6A0	53	9.00E-25	No
	VSAL_p840_08	843	Conjugative transfer protein (*traC*)	*E. coli*	P18004	30	3.40E-62	2265751
	VSAL_p840_09	115	Conjugative transfer protein (*trbI*)	*E. coli*	P18006	33	1.20E-05	1355084
	VSAL_p840_10	218	Conjugative transfer protein (*traW*)	*E. coli*	P18472	35	3.30E-07	1355084
	VSAL_p840_11	334	Conjugative transfer protein (*traU*)	*E. coli*	P18471	49	2.60E-77	2198250

pVS840	*VSAL_p840_12*	255	Putative conjugative transfer protein (*trbC*)	*E. coli*	P18473	23	1.10E-01	2050638
	VSAL_p840_13	572	Conjugative transfer protein (*traN*)	*E. coli*	P24082	42	3.40E-43	1593622
	VSAL_p840_14	247	Conjugative transfer protein (*traF*)	*E. coli*	P14497	30	1.40E-17	3042757
	VSAL_p840_15	255	Putative membrane protein	None				
	VSAL_p840_16	141	Conjugative transfer protein (*trbB*)	*E. coli*	P18035	37	6.20E-10	2536655
	VSAL_p840_17	461	Conjugative transfer protein (*traH*)	*E. coli*	P15069	34	7.10E-50	2656408
	VSAL_p840_18	931	Conjugative transfer protein (*traG*)	*E. coli*	P33790	26	2.80E-50	1348105
	VSAL_p840_19	165	Putative exported protein	*V. vulnificus*	Q7MBM2	27	4.10E-03	No
	VSAL_p840_20	692	Conjugative transfer protein (*traD*)	*E. coli*	P09130	36	4.80E-76	2680768
**Other**								
	VSAL_I1141	163	V10 pilin	*V. cholerae*	Q93RE5	42	1.70E-15	11497216
	VSAL_I2294	224	Putative type IV pilus assembly protein PilZ	*S. frigidimarina*	Q07YX2	32	1.90E-17	No
	VSAL_I2444	209	Accessory colonization factor precursor (*acfA*)	*V. cholerae*	Q57345	35	1.70E-20	7737517
	VSAL_p840_23	145	Transglycosylase (*pilT*)	*E. coli*	Q93D61	44	1.70E-21	12011003
<u>**LPS and cell capsule**</u>								
**Lipopolysaccharide biosynthesis**								
	VSAL_I0159	313	ADP-L-glycero-D-manno-heptose-6-epimerase (*rfaD*)	*E. coli*	P67910	74	4.50E-84	2198271
	VSAL_I0160	411	O-antigen ligase (*rfaL*)	*E. coli*	P27243	24	2.30E-02	1624462
	VSAL_I0161	356	ADP-heptose-LPS heptosyltransferase II (*rfaF*)	*E. coli*	P37692	52	3.70E-68	1624462
	VSAL_I0162	420	3-deoxy-D-manno-octulosonic-acid transferase (*kdtA*)	*E. coli*	P0AC75	41	6.50E-60	2033061
	VSAL_I0183	356	Glycosyl transferase (*waaC*)	*V. cholerae*	Q9KVC1	49	9.20E-67	15469898
	VSAL_I0184	260	Glycosyl transferase (*wavB*)	*V. cholerae*	Q9KVC2	60	3.80E-58	15469898
	VSAL_I0185	343	Glycosyl transferase	*R. meliloti*	Q9R9N0	26	1.40E-10	15469898
	VSAL_I0186	162	Phosphopantetheine adenylyltransferase (*kdtB*)	*E. coli*	P0A6I6	58	1.20E-31	2033061
	VSAL_I0238	343	Glycosyl transferase	*R. meliloti*	Q9R9N0	26	1.40E-10	15469898
	VSAL_I0239	260	Glycosyl transferase (*wavB*)	*V. cholerae*	Q9KVC2	44	1.10E-38	15469898
	VSAL_I0240	356	Glycosyl transferase (*waaC*)	*V. cholerae*	Q9KVC1	49	9.20E-67	15469898
	VSAL_I0261	420	3-deoxy-D-manno-octulosonic-acid transferase (*kdtA*)	*E. coli*	P0AC75	41	6.50E-60	2033061
	VSAL_I0262	356	ADP-heptose-LPS heptosyltransferase II (*rfaF*)	*E. coli*	P37692	52	3.70E-68	1624462
	VSAL_I0263	411	O-antigen ligase (*rfaL*)	*E. coli*	P27243	24	2.30E-02	1624462
	VSAL_I0264	313	ADP-L-glycero-D-manno-heptose-6-epimerase (*rfaD*)	*E. coli*	P67910	74	4.50E-84	2198271
	VSAL_I0502	185	3-deoxy-d-manno-octulosonate 8-phosphate phosphatase (*kdsC*)	*E. coli*	P0ABZ4	61	4.30E-38	12805358
	VSAL_I0503	324	Arabinose 5-phosphate isomerase (*kdsD*)	*E. coli*	P45395	62	2.40E-66	12805358
	VSAL_I0794	284	2-dehydro-3-deoxyphosphooctonate aldolase (*kdsA*)	*E. coli*	P0A715	79	1.00E-84	3039295
	VSAL_I0826	165	Transcriptional activator (*rfaH*)	*E. coli*	P0AFW0	50	1.20E-24	1584020
	VSAL_I0885	183	D,D-heptose 1,7-bisphosphate phosphatase (*gmhB*)	*V. cholerae*	Q9KTJ4	71	1.30E-50	12101286
	VSAL_I2175	313	Lipid A biosynthesis acyltransferase *(msbB)*	*E. coli*	P24205	38	1.60E-46	9099672
	VSAL_I2414	383	Lipid-A-disaccharide synthase (*lpxB*)	*E. coli*	P10441	58	1.10E-81	2824445
	VSAL_I2415	262	UDP-N-acetylglucosamine acyltransferase (*lpxA*)	*E. coli*	P0A722	58	3.00E-56	3277952
	VSAL_I2416	159	(3R)-hydroxymyristoyl-[acyl-carrier-protein] dehydratase (*fabZ*)	*E. coli*	P0A6Q6	67	2.30E-36	7806516
	VSAL_I2417	339	UDP-3-O-[3-hydroxymyristoyl] glucosamine N-acyltransferase (*lpxD*)	*E. coli*	P21645	62	8.30E-74	8366125
	VSAL_I2418	171	Outer membrane protein	*Y. pestis*	P58607	39	2.00E-16	No
	VSAL_I2419	806	Outer membrane protein assembly factor	*E. coli*	P0A940	56	2.70E-167	15951436

	VSAL_I2639	305	UDP-3-O-[3-hydroxymyristoyl] N-acetylglucosamine deacetylase (*lpxC*)	*E. coli*	P0A725	77	2.00E-91	8530464
	VSAL_I2676	476	Bifunctional protein (*hldE*)	*E. coli*	P76658	67	1.40E-112	10629197
	VSAL_I2894	807	Glycerol-3-phosphate acyltransferase (*plsB*)	*E. coli*	P0A7A7	58	1.10E-180	9515909
	**VSAL_II0518**	318	Lipid A biosynthesis lauroyl acyltransferase (*htrB*)	*E. coli*	P0ACV0	45	1.40E-47	2203778
	**VSAL_II0732**	249	3-deoxy-manno-octulosonate cytidylyltransferase (*kdsB*)	*E. coli*	P04951	65	3.40E-50	7543480
	**VSAL_II0733**	56	Hypothetical protein	*A. fischeri*	Q5E0F0	96	1.00E-18	No
	**VSAL_II0734**	335	Tetraacyldisaccharide 4'-kinase (*lpxK*)	*E. coli*	P27300	55	1.60E-57	9575203
	**VSAL_II0735**	768	Lipid A export ATP-binding/permease protein (*msbA*)	*E. coli*	P60752	63	6.70E-115	8094880
**Exopolysaccharide biosynthesis**								
	VSAL_I0165	367	Putative pseudaminic acid biosynthesis protein	*C. jejuni*	Q939J7	36	6.60E-39	16573682
	VSAL_I0166*	230	Putative acylneuraminate cytidylyltransferase	*E. coli*	Q93NQ1	42	3.80E-21	11404020
	VSAL_I0167	352	Nucleotidyl transferase (*ptmE*)	*C. coli*	Q7X521	43	5.90E-31	8825781
	VSAL_I0168	214	Putative acetyltransferase	*A. fischeri*	Q5E8K7	89	6.80E-65	No
	VSAL_I0169	357	N-acetylneuraminic acid synthase (*ptmC*)	*C. coli*	Q7X523	52	4.50E-31	8825781
	VSAL_I0170	394	UDP-N-acetylglucosamine 2-epimerase (*ptmD*)	*C. coli*	Q7X522	42	1.70E-50	8825781
	VSAL_I0171	381	Aminotransferase	*P. aeruginosa*	Q8KNA4	60	1.60E-91	12057956
	VSAL_I0172	396	Polysaccharide biosynthesis protein	*V. cholerae*	O05349	27	7.20E-15	9079898
	VSAL_I0173	373	Hypothetical protein	*V. vulnificus*	Q7MPQ6	44	9.00E-57	No
	VSAL_I0174	380	Lipopolysaccharide biosynthesis protein (*wavR*)	*V. cholerae*	Q8RTH1	65	3.20E-98	15469898
	VSAL_I0176	279	Putative glycosyl transferase	*V. cholerae*	Q8RTH7	48	1.30E-43	11953379
	VSAL_I0177	185	dTDP-6-deoxy-D-xylo-4-hexulose-3,5-epimerase (*rmlC*)	*V. cholerae*	Q842R9	81	1.00E-56	12949172
	VSAL_I0178	296	dTDP-6-deoxy-l-mannose-dehydrogenase (*rmlD*)	*V. cholerae*	Q842N3	58	2.30E-59	12949172
	VSAL_I0179	294	Glucose-1-phosphate thymidyl transferase (*rmlA*)	*V. cholerae*	Q842Q4	84	9.30E-96	12949172
	VSAL_I0180	363	dTDP-glucose 4,6-dehydratase (*rmlB*)	*V. cholerae*	Q842R1	84	1.30E-119	12949172
	VSAL_I0181	617	Membrane associated sulfatase	*A. fischeri*	Q5E8L4	88	2.10E-195	No
	VSAL_I0182	234	Lipopolysaccharide core oligosaccharide biosynthesis protein (*wavC*)	*V. cholerae*	Q8RJM8	66	2.00E-69	11953379
	VSAL_I0241	234	Lipopolysaccharide core oligosaccharide biosynthesis protein (*wavC*)	*V. cholerae*	Q8RJM8	66	2.00E-69	11953379
	VSAL_I0242	617	Membrane associated sulfatase	*A. fischeri*	Q5E8L4	88	2.10E-195	No
	VSAL_I0243	363	dTDP-glucose 4,6-dehydratase (*rmlB*)	*V. cholerae*	Q842R1	84	1.30E-119	12949172
	VSAL_I0244	294	Glucose-1-phosphate thymidyl transferase (*rmlA*)	*V. cholerae*	Q842Q4	84	9.30E-96	12949172
	VSAL_I0245*	296	dTDP-6-deoxy-l-mannose-dehydrogenase (*rmlD*)	*V. cholerae*	Q842N3	58	2.30E-59	12949172
	VSAL_I0246	185	dTDP-6-deoxy-D-xylo-4-hexulose-3,5-epimerase (*rmlC*)	*V. cholerae*	Q842R9	81	1.00E-56	12949172
	VSAL_I0247	279	Putative glycosyl transferase	*V. cholerae*	Q8RTH7	48	1.30E-43	11953379
	VSAL_I0249	380	Lipopolysaccharide biosynthesis protein (*wavR*)	*V. cholerae*	Q8RTH1	65	3.20E-98	15469898
	VSAL_I0250	373	Hypothetical protein	*V. vulnificus*	Q7MPQ6	44	9.00E-57	No
	VSAL_I0251	396	Polysaccharide biosynthesis protein	*V. cholerae*	O05349	27	7.20E-15	9079898
	VSAL_I0252	381	Aminotransferase	*P. aeruginosa*	Q8KNA4	60	1.60E-91	12057956
	VSAL_I0253	394	UDP-N-acetylglucosamine 2-epimerase (*ptmD*)	*C. coli*	Q7X522	42	1.70E-50	8825781
	VSAL_I0254	357	N-acetylneuraminic acid synthase (*ptmC*)	*C. coli*	Q7X523	52	4.50E-31	8825781
	VSAL_I0255	214	Putative acetyltransferase	*A. fischeri*	Q5E8K7	89	6.80E-65	No
	VSAL_I0256	352	Nucleotidyl transferase (*ptmE*)	*C. coli*	Q7X521	43	5.90E-31	8825781
	VSAL_I0257*	230	Putative acylneuraminate cytidylyltransferase	*E. coli*	Q93NQ1	42	3.80E-21	11404020
	VSAL_I0258	367	Putative pseudaminic acid biosynthesis protein	*C. jejuni*	Q939J7	36	6.60E-39	16573682
	VSAL_I0265	484	Putative metallo-beta-lactamase (*wbfZ*)	*V. cholerae*	O87142	59	1.20E-74	10521656
	VSAL_I0270	174	Putative lipoprotein (*wbfA*)	None				

		VSAL_I0272	735	Putative outer membrane protein (*wbfB*)	*V. cholerae*	O87145	50	4.40E-145	10521656
		VSAL_I0273	254	Exported protein (*wbfC*)	*V. cholerae*	Q56655	30	2.40E-09	10521656
		VSAL_I0274	221	Putative lipoprotein (*wbfD*)	*V. cholerae*	Q56656	34	1.30E-18	10521656
		VSAL_I0275	85	Membrane protein	*A. fischeri*	Q5E8J0	71	2.00E-10	No
		VSAL_I0276	455	Putative membrane protein	None				
		VSAL_I0277	869	Periplasmic protein involved in polysaccharide export (*wbfF*)	*V. cholerae*	Q56658	38	5.50E-58	8793876 and 10521656
		VSAL_I0278	325	O-antigen length determinant protein (*wzz*)	*V. cholerae*	O87149	53	3.80E-52	10521656
		VSAL_I3018	371	dTDP-glucose 4,6-dehydratase (*rmlB*)	*V. cholerae*	Q842N5	85	7.10E-124	12949172 and 16113305
	GI-VSA10	VSAL_I3019	295	Glucose-1-phosphate thymidyl transferase (*rmlA*)	*V. cholerae*	Q842R4	83	1.10E-93	12949172 and 16113305
		VSAL_I3020*	293	dTDP-6-deoxy-l-mannose-dehydrogenase (*rmlD*)	*V. cholerae*	Q842P5	68	2.00E-72	12949172 and 16113305
		VSAL_I3021	184	dTDP-6-deoxy-D-xylo-4-hexulose-3,5-epimerase (*rmlC*)	*V. cholerae*	Q842R2	84	7.60E-58	12949172 and 16113305
		VSAL_I3024	292	Putative glycosyl transferase	*E. coli*	Q9X4C3	33	1.50E-10	10200954
		VSAL_I3025	427	Membrane protein	None				
		VSAL_I3026	280	Putative glycosyl transferase (*wbfP*)	*S. boydii*	Q6QQW8	30	2.90E-07	15109730
	GI-VSA10	VSAL_I3027	267	Putative glycosyl transferase (*wbfO*)	*V. cholerae*	P74947	31	1.50E-07	10521656
		VSAL_I3028	190	Putative lipopolysaccharide biosynthesis O-acetyl transferase	*E. coli*	P37750	35	2.00E-08	7517391
		VSAL_I3029	378	Putative NAD dependent epimerase/dehydratase (*wbfW*)	*V. cholerae*	Q56626	26	9.00E-22	8820651
		VSAL_I3030	333	Putative lipopolysaccharide biosynthesis protein	*A. hydrophila*	Q8KXE8	29	1.80E-14	11953367
		VSAL_I3031	320	O-antigen biosynthetic gene (*wbfT*)	*V. cholerae*	Q56623	41	3.70E-33	8820651
		VSAL_I3032	182	Capsular polysaccharide synthesis enzyme (*wbfU*)	*V. cholerae*	Q56624	88	1.90E-56	8820651
		VSAL_I3033	644	Polysaccharide biosynthesis protein (*wbfY*)	*V. cholerae*	Q56627	71	6.80E-152	8820651
		VSAL_p54_01	642	Acyltransferase	*S. typhimurium*	P74874	34	6.30E-54	8830685
		VSAL_p43_02	321	Acyltransferase	Bacteriophage Sf6	P23214	29	4.20E-11	2014005
**Capsule polysaccharide biosynthesis**									
		VSAL_I3010	207	Putative capsular polysaccharide biosynthesis protein (*neuD*)	*E. coli*	Q46674	68	5.20E-49	7814319
		VSAL_I3011	346	Putative sialic acid synthase (*neuB*)	*E. coli*	Q46675	69	6.70E-84	7814319
	GI-VSA10	VSAL_I3012	417	N-acylneuraminate cytidylyltransferase (*neuA*)	*E. coli*	P13266	52	2.30E-77	2549035
		VSAL_I3013	388	Polysialic acid biosynthesis protein (*neuC*)	*E. coli*	Q47400	59	2.90E-84	1729218
		VSAL_I3014	422	Putative exopolysaccharide biosynthesis protein	*A. punctata*	Q7BR79	56	3.90E-79	11119490
		VSAL_I3015	298	Hypothetical protein	*R. baltica*	Q7ULW0	35	2.00E-18	No
		VSAL_I3016	321	Lipooligosaccharide sialyltransferase	*E. coli*	Q93NP9	29	5.90E-14	11404020
		**VSAL_II0295**	214	Bacterial sugar transferase	*A. fischeri*	Q5DYN9	88	5.90E-71	No
		**VSAL_II0296**	394	Putative transmembrane glycosyl transferase	*A. fischeri*	Q5DYP0	70	2.10E-105	No
		**VSAL_II0297**	362	Putative glycosyl transferase	*A. fischeri*	Q5DYP1	68	8.40E-100	No
		**VSAL_II0298**	499	Putative membrane protein	*A. fischeri*	Q5DYP2	81	7.40E-139	No
		**VSAL_II0299**	385	Putative glycosyl transferase	*V. cholerae*	Q06BC1	25	3.10E-16	17362509
		**VSAL_II0300**	251	Hypothetical protein	*A. fischeri*	Q5DYP4	85	3.00E-83	No
		**VSAL_II0301**	464	O-antigen polymerase	*A. fischeri*	Q5DYP5	80	2.00E-132	No
		**VSAL_II0302**	480	Putative capsular polysaccharide synthesis protein	*A. fischeri*	Q5DYP6	69	5.70E-119	No
		**VSAL_II0303**	387	Putative glycosyl transferase	*A. fischeri*	Q5DYP7	72	8.90E-124	No
		**VSAL_II0304**	426	Putative glycosyl transferase	*A. fischeri*	Q5DYP8	74	1.10E-125	No

	**VSAL_II0305**	330	Putative glycosyl transferase	*S. pneumoniae*	Q9AHA1	24	2.00E-05	11179285
	**VSAL_II0306**	501	Transcriptional regulatory protein (*luxO*)	*V. harveyi*	P54299	49	4.90E-79	8065259
	**VSAL_II0307**	755	Response regulator, histidine kinase	*A. fischeri*	Q5DYQ1	66	4.40E-145	No
	**VSAL_II0308**	506	Putative response regulator	*A. fischeri*	Q5DYQ2	66	6.40E-133	No
	**VSAL_II0309**	235	Putative capsular polysaccharide synthesis protein	*S. pneumoniae*	P72512	27	7.40E-05	8682774
	**VSAL_II0310**	703	Polysaccharide biosynthesis/export protein (*kpsD*)	*E. coli*	P42213	24	3.30E-07	8397187
	**VSAL_II0311**	275	Outer membrane protein	*A. fischeri*	Q5DYQ5	89	3.00E-93	No
**<u>Secretion</u>**								
**T1SS**								
	VSAL_I2675	441	Outer membrane protein (*tolC*)	*V. cholerae*	Q9K2Y1	74	1.50E-114	15547287
	**VSAL_II0528**	431	Hemolysin secretion protein precursor HlyB	*V. cholerae*	P15492	46	9.10E-24	2162464
**T1SSI**								
	**VSAL_II1098**	707	ABC transporter ATP-binding protein	*V. cholerae*	Q9KKL9	78	3.20E-180	No
	**VSAL_II1099**	641	Membrane signal transduction protein	*V. cholerae*	Q9KKM1	56	4.20E-78	No
	**VSAL_II1100***	220	Hypothetical protein	*V. cholerae*	Q9KKM2	67	8.40E-57	No
	**VSAL_II1101**	463	Secretion protein, HlyD family	*V. cholerae*	Q9KKM3	67	4.60E-109	15547287
	**VSAL_II1102**	717	Putative exported ATPase protein	*V. cholerae*	Q9KKJ5	36	4.10E-57	No
**T1SSII**								
	**VSAL_II0055**	104	Hypothetical protein	*A. fischeri*	Q5DYR3	59	1.30E-18	No
	**VSAL_II0056**	442	Putative type I secretion protein, HlyD family	*P. profundum*	Q1Z9Z3	55	2.10E-83	No
	**VSAL_II0057**	741	Putative membrane associated response regulator	*P. profundum*	Q6LGG3	39	1.20E-36	No
	**VSAL_II0058**	456	Putative type I toxin secretion system, outer membrane efflux protein	*P. aeruginosa*	Q03027	21	1.80E-12	1427098
	**VSAL_II0059**	527	Putative type I toxin secretion system, membrane transport protein	*P. fluorescens*	O67993	23	5.10E-09	9501431
	**VSAL_II0060**	717	Putative type I toxin secretion system, ATP-binding protein	*S. marcescens*	Q54456	24	5.40E-21	7592412
**T1SSIII**								
	**VSAL_II0063**	566	Putative type I secretion system, ATP-binding protein	*Y. ruckeri*	Q8VLR8	38	1.20E-60	12101310
	**VSAL_II0064**	429	Putative type I secretion protein, HlyD family	*P. profundum*	Q1Z578	58	3.00E-85	No
	**VSAL_II0065**	80	Membrane protein	None				
	**VSAL_II0066**	252	Membrane protein	None				
**T2SSI**								
	VSAL_I2914	253	General secretion pathway protein N (*epsN*)	*V. cholerae*	P41852	42	3.50E-39	9371445
	VSAL_I2915	163	General secretion pathway protein M (*epsM*)	*V. cholerae*	P41851	48	1.10E-29	9371445
	VSAL_I2916	411	General secretion pathway protein L (*epsL*)	*V. cholerae*	P45782	42	6.90E-24	9371445
	VSAL_I2917	339	General secretion pathway protein K (*epsK*)	*V. cholerae*	P45781	60	3.40E-76	9371445
	VSAL_I2918	215	General secretion pathway protein J (*epsJ*)	*V. cholerae*	P45776	54	3.80E-43	9371445
	VSAL_I2919	125	General secretion pathway protein I (*epsI*)	*V. cholerae*	P45775	62	3.00E-24	9371445
	VSAL_I2920	198	General secretion pathway protein H (*epsH*)	*V. cholerae*	P45774	46	1.00E-30	9371445
	VSAL_I2921	149	General secretion pathway protein G (*epsG*)	*V. cholerae*	P45773	85	7.80E-46	9371445
	VSAL_I2922	405	General secretion pathway protein F (*epsF*)	*V. cholerae*	P45780	72	7.00E-110	9371445
	VSAL_I2923	499	General secretion pathway protein E (*epsE*)	*V. cholerae*	P37093	78	3.50E-127	9371445
	VSAL_I2924	675	General secretion pathway protein D (*epsD*)	*V. cholerae*	P45779	79	3.90E-184	9371445
	VSAL_I2925	303	General secretion pathway protein C (*epsC*)	*V. cholerae*	P45777	48	4.20E-45	9371445

**Flp-type pilus (Subtype of T2SS)**									
	GI-VSA5	**VSAL_II0366**	69	Fimbrial protein, Flp/Fap pilin component Flp1	*A. actinomycetemcomitans*	O66149	32	4.00E-03	12717435
		**VSAL_II0367**	144	Type IV leader peptidase (*tadV*)	*A. actinomycetemcomitans*	Q7X0M3	33	5.70E-07	12717435
		**VSAL_II0368**	260	Putative Flp pilus assembly protein CpaB	*A. fischeri*	Q5E107	60	1.50E-49	No
		**VSAL_II0369**	445	Type II/III secretion system protein (*rcpA*)	*A. actinomycetemcomitans*	Q7X0M1	36	7.20E-27	12717435
		**VSAL_II0370**	169	Putative lipoprotein	*A. fischeri*	Q5E105	56	1.70E-36	No
		**VSAL_II0371**	405	Type II secretion system protein (*tadZ*)	*A. actinomycetemcomitans*	Q8GCZ9	25	2.00E-03	12717435
		**VSAL_II0372**	428	Type II secretion system protein (*tadA*)	*A. actinomycetemcomitans*	Q9XC06	53	1.30E-77	12717435
		**VSAL_II0373**	303	Type II secretion system protein (*tadB*)	*A. actinomycetemcomitans*	Q9XC05	37	5.30E-28	12717435
		**VSAL_II0374**	292	Type II secretion system protein (*tadC*)	*A. actinomycetemcomitans*	Q9S4A8	33	7.00E-23	12717435
		**VSAL_II0375**	247	Putative secretion system protein (*tadD*)	*A. actinomycetemcomitans*	Q9S4A7	30	7.10E-13	12717435
		**VSAL_II0376**	162	Membrane associated secretion system protein (*tadE*)	*A. actinomycetemcomitans*	Q7X0L4	24	7.50E-01	12717435
		**VSAL_II0377**	179	Membrane associated secretion system protein (*tadF*)	*A. actinomycetemcomitans*	Q9S4A5	31	7.20E-08	12717435
		**VSAL_II0378**	422	Membrane associated secretion system protein (*tadG*)	*A. actinomycetemcomitans*	Q9S4A4	21	4.60E-04	12717435
**T6SSI**									
	GI-VS5	VSAL_I1112	217	Serine/threonine phosphoprotein phosphatase (*vasL*)	*P. aeruginosa*	Q9I355	36	9.70E-18	16763151
		VSAL_I1113*	1172	Putative type VI secretion protein (*vasK*)	*P. aeruginosa*	Q9I356	36	7.20E-150	16763151
		VSAL_I1114	275	Putative membrane protein (*vasF*)	*P. aeruginosa*	Q9I357	50	1.30E-50	16763151
		VSAL_I1115	440	Putative type VI secretion protein (*vasE*)	*P. aeruginosa*	Q9I358	47	1.10E-82	16763151
		VSAL_I1116	162	Putative type VI secretion protein (*vasD*)	*P. aeruginosa*	Q9I359	40	3.00E-14	16763151
		VSAL_I1117	453	Putative type VI secretion protein (*vasC*)	*P. aeruginosa*	Q9I360	32	1.20E-07	16763151
		VSAL_I1118*	507	Transcriptional regulatory protein (*vasH*)	*P. aeruginosa*	Q9I362	40	3.00E-44	16763151
		VSAL_I1119	872	Chaperone ClpB (*vasG*)	*Y. enterocolitica*	Q9F746	42	9.00E-111	10986262
		VSAL_I1120	309	Putative type VI secretion protein (*vasB*)	*P. aeruginosa*	Q9I364	48	2.80E-56	16763151
		VSAL_I1121	583	Putative type VI secretion protein (*vasA*)	*V. cholerae*	Q9KN55	36	3.30E-74	16432199
		VSAL_I1122	141	Putative type VI secretion protein (*vasS*)	*P. aeruginosa*	Q9I366	33	0.0001	16763151
		VSAL_I1123	491	Putative type VI secretion protein (*vasR*)	*P. aeruginosa*	Q9I367	73	5.40E-150	16763151
		VSAL_I1124	164	Putative type VI secretion protein (*vasQ*)	*P. aeruginosa*	Q9I368	61	2.90E-31	16763151
		VSAL_I1125	513	Putative type VI secretion protein (*vasJ*)	*P. aeruginosa*	Q9I369	28	4.80E-40	16763151
		VSAL_I1357	172	Secreted hemolysin co-regulated protein Hcp-2	*V. cholerae*	P72350	54	2.30E-35	8557353 and 16432199
		VSAL_I1358	754	VgrG protein VgrG-2	*V. cholerae*	Q9KNE7	34	2.20E-68	16432199
		VSAL_I1359	197	Hypothetical protein	None				
		VSAL_I1360*	664	Putative peptidoglycan-binding LysM	None				
**Duplicated part of T6SSI**									
	pVS840	VSAL_p840_35	172	Secreted hemolysin co-regulated protein Hcp-2	*V. cholerae*	P72350	53	4.00E-35	8557353
		VSAL_p840_36	754	VgrG protein VgrG-2	*V. cholerae*	Q9KNE7	36	1.90E-69	16432199
		VSAL_p840_37	186	Hypothetical protein	None				
		VSAL_p840_38	814	Putative peptidoglycan-binding LysM	None				

T6SSII

	VSAL_I1167	186	Putative type VI secretion protein (*vasU-1*)	*V. splendidus*	A3USE8	64	7.80E-49	No
	VSAL_I1168	98	Putative type VI secretion protein (*vasV-1*)	*V. splendidus*	A3USE9	89	6.80E-36	No
	VSAL_I1169	461	Putative type VI secretion protein VasJ-1	*R. leguminosarum*	Q93ED3	24	0.0033	12580282
	VSAL_I1170	130	Secreted hemolysin-coregulated protein Hcp-3	*P. aeruginosa*	Q9I747	24	0.52	16763151
	VSAL_I1171	169	Putative type VI secretion protein VasQ-1	*R. leguminosarum*	Q93ED2	48	5.20E-21	12580282
	VSAL_I1172	493	Putative type VI secretion protein VasRA-1	*R. leguminosarum*	Q93ED1	51	1.20E-94	12580282
	VSAL_I1173	453	Putative type VI secretion protein VasRB-1	*R. leguminosarum*	Q93ED1	28	1.30E-19	12580282
	VSAL_I1174	140	Putative type VI secretion protein VasS-1	None				
GI-VS6	VSAL_I1175	593	Putative type VI secretion protein VasA-1	*R. leguminosarum*	Q93EC7	28	1.00E-44	12580282
	VSAL_I1176	327	Putative type VI secretion protein VasB-1	*P. aeruginosa*	Q9I743	20	0.0056	16763151
	VSAL_I1177	872	Chaperone ClpB-1	*Y. enterocolitica*	Q9F746	42	4.70E-112	10986262
	VSAL_I1178	52	Putative type VI secretion protein VasV-1	None				
	VSAL_I1179	314	Putative type VI secretion protein VasC-1	*P. aeruginosa*	Q9I751	30	4.9	16763151
	VSAL_I1180	154	Putative type VI secretion protein VasD-1	*P. aeruginosa*	Q9I752	34	2.80E-07	16763151
	VSAL_I1181	438	Putative type VI secretion protein VasE-1	*R. leguminosarum*	Q93EC4	29	7.30E-22	12580282
	VSAL_I1182	374	Putative type VI secretion protein VasF-1	*P. aeruginosa*	Q9I357	27	8.20E-12	16763151
	VSAL_I1183*	1144	Putative type VI secretion protein VasK-1	*P. aeruginosa*	Q9I356	26	1.10E-56	16763151
	VSAL_I1184	432	Transposase	*E. agglomerans*	P71168	57	4.10E-104	No
	VSAL_I1185	618	Putative type VI secretion protein VasX-1	*V. splendidus*	A3USC8	45	1.20E-29	No
	VSAL_I1202	173	Secreted hemolysin co-regulated protein Hcp-1	*V. cholerae*	P72350	54	1.10E-34	8557353 and 16432199
	VSAL_I1744*	695	VgrG protein VgrG-1	*V. cholerae*	Q9KNE7	42	1.20E-93	16432199

Exotoxin

VSAL_I0411	590	Hemolysin (*vah5*)	*V. anguillarum*	Q4LEW4	55	2.80E-126	16126365
VSAL_I0993	291	Hemolysin (*vah2*)	*V. anguillarum*	Q4LEW7	80	1.00E-74	16126365
VSAL_I0818	207	ATP-dependent protease (*clpP*)	*S. typhimurium*	P0A1D7	74	1.10E-57	11292737
VSAL_I1710*	4403	Putative hemolysin	*A. fischeri*	Q5E4P5	68	0.00E+00	No
VSAL_I0049	215	Hemolysin (h*lyIII*)	*V. vulnificus*	Q7X3Y6	69	7.40E-48	15386100
**VSAL_II0020**	2890	Hemolysin-type calcium-binding protein	*S. hyicus*	Q4ZHU0	25	2.40E-25	16000737
**VSAL_II0423**	323	Exported serine protease, trypsin elastase	*A. fischeri*	Q5E0V3	60	4.80E-72	No
VSAL_p320_15	205	Putative peptidase, S24-like	Bacteriophage phi-80	P14819	29	1.50E-07	3172225

Iron acquisition

Heme uptake

VSAL_I1748	176	Heme uptake and utilization protein (*huvZ*)	*V. anguillarum*	Q70YH4	69	5.00E-42	15342586
VSAL_I1749	168	Heme uptake and utilization protein (*huvX*)	*V. anguillarum*	Q70YH3	60	1.40E-34	15342586
VSAL_I1750	440	Putative coproporphyrinogen oxidase PhuW	*V. parahaemolyticus*	Q9XCY7	59	9.30E-98	10348876
VSAL_I1751*	248	TonB protein (*tonB1*)	*V. anguillarum*	Q70YH2	45	6.10E-33	15342586
VSAL_I1752	226	TonB system transport protein (*exbB1*)	*V. anguillarum*	Q70YH1	54	1.40E-40	15342586
VSAL_I1753	141	TonB system transport protein (*exbD1*)	*V. anguillarum*	Q70YH0	60	1.30E-24	15342586
VSAL_I1754	290	Heme transporter protein (*huvB*)	*V. anguillarum*	Q70YG9	62	8.90E-52	15342586
VSAL_I1755	345	Heme transporter protein (*huvC*)	*V. anguillarum*	Q70YG8	72	3.90E-71	15342586
VSAL_I1756	260	Heme transporter protein (*huvD*)	*V. anguillarum*	Q659V4	57	5.70E-47	15342586

		**VSAL_II0110**	693	TonB dependent receptor	*S. typhimurium*	Q56145	27	8.70E-31	10103258
		**VSAL_II0111**	255	Putative exported protein	*V. cholerae*	Q9ZHW2	57	6.50E-47	9781885 and 11169119
		**VSAL_II0112**	451	Biopolymer transport protein (*TolR2*)	*V. cholerae*	Q9ZHW1	58	2.00E-88	9781885 and 11169119
		**VSAL_II0113**	169	TonB system transport protein (*exbB2*)	*V. cholerae*	Q9ZHW0	74	1.30E-46	9781885 and 11169119
		**VSAL_II0114**	134	TonB system transport protein (*exbD2*)	*V. cholerae*	Q9ZHV9	88	6.10E-39	9781885 and 11169119
		**VSAL_II0115**	206	TonB protein (*tonB2*)	*V. cholerae*	Q9ZHV8	53	4.50E-39	9781885 and 11169119
		**VSAL_II0116**	400	Putative exported protein	*V. cholerae*	Q9ZHV7	51	4.10E-72	9781885 and 11169119
		**VSAL_II0901**	207	TonB3 protein (*tonB3*)	*V. anguillarum*	Q5SDB0	62	7.50E-45	15557661
		**VSAL_II0902**	134	TonB system transport protein (*exbD3*)	*V. anguillarum*	Q5SDB1	90	1.90E-40	15557661
		**VSAL_II0903**	177	TonB system transport protein (*exbB3*)	*V. anguillarum*	Q5SDB2	74	1.70E-47	15557661
		**VSAL_II0904**	453	Biopolymer transport protein (*tolR3*)	*V. anguillarum*	Q5SDB3	62	7.10E-94	15557661
**Siderophore synthesis/uptake**									
		**VSAL_II0905**	255	Putative exported protein	*A. fischeri*	Q5DZE6	79	3.60E-64	No
		**VSAL_II0906**	671	Iron(III) ABC transporter, membrane permease (*hatB*)	*V. hollisae*	Q4H463	38	4.20E-65	16809025
		**VSAL_II0907***	314	Iron(III) ABC transporter, plasmic binding protein (*hatD*)	*V. hollisae*	Q4H464	32	1.30E-25	16809025
		**VSAL_II0908**	255	Iron(III) ABC transporter, ATP-binding protein (*hatC*)	*V. hollisae*	Q4H465	44	1.40E-34	16809025
		**VSAL_II0909**	716	Ferrioxamine B receptor	*V. vulnificus*	Q59HH9	59	1.10E-164	16000245
		VSAL_I0134	515	L-2,4-diaminobutyrate decarboxylase	*R. meliloti*	Q9Z3R1	36	3.00E-36	11274118
		VSAL_I0135	442	Siderophore biosynthesis protein (*iucD*)	*E. coli*	Q47318	26	6.20E-41	8003107
		VSAL_I0136	818	Siderophore biosynthesis protein (*iucC*)	*E. coli*	P11295	33	1.00E-45	3275632
		VSAL_I0137	721	TonB-dependent iron-siderophore receptor (*fhuE*)	*E. coli*	P16869	35	6.90E-71	2162465
	GI-VS3	VSAL_I0138	316	Iron-siderophore ABC transporter (*fepB*)	*E. coli*	P0AEL6	22	4.80E-05	2529253 and 2651410
		VSAL_I0139	366	Iron-siderophore ABC transporter (*fepD*)	*E. coli*	P23876	40	1.70E-34	2651410
		VSAL_I0140	341	Iron-siderophore ABC transporter (*fecD*)	*E. coli*	P15029	36	9.80E-24	2651410
		VSAL_I0141	268	Iron-siderophore ABC transporter (*fepC*)	*E. coli*	P23878	44	2.30E-33	2651410
		**VSAL_II0150**	255	Ferrichrome transport ATP-binding protein (*fhuC*)	*V. mimicus*	Q76BT0	75	2.60E-66	15215626
		**VSAL_II0151**	296	Ferrichrome-binding periplasmic protein (*fhuD*)	*V. mimicus*	Q76BS9	56	4.30E-63	15215626
		**VSAL_II0152**	662	Ferrichrome transport protein (*fhuB*)	*V. mimicus*	Q76BS8	66	3.60E-144	15215626
		**VSAL_II0273**	546	Siderophore biosynthetis protein (*iucA*)	*V. mimicus*	Q76BS7	61	1.30E-135	15215626
		**VSAL_II0274**	303	Siderophore biosynthetis protein (*iucB*)	*V. mimicus*	Q76BS6	60	5.00E-77	15215626
		**VSAL_II0275***	585	Siderophore biosynthetis protein (*iucC*)	*V. mimicus*	Q76BS5	64	1.20E-145	15215626
		**VSAL_II0278**	437	Siderophore biosynthetis protein (*iucD*)	*V. mimicus*	Q76BS4	70	3.40E-123	15215626
		**VSAL_II0279***	723	Ferric aerobactin receptor (*iutA*)	*V. mimicus*	Q76BS3	73	1.60E-209	15215626
**Iron transport**									
		VSAL_I2257	75	Ferrous iron transport protein (*feoA*)	*S. typhimurium*	Q7CPK6		1.00E-03	8890205
		VSAL_I2258	758	Ferrous iron transport protein (*feoB*)	*S. typhimurium*	P74884	43	2.50E-107	8890205
		VSAL_I2586	346	Iron(III) ABC transporter (*fbpC*)	*P. haemolytica*	Q9ZHY0	50	7.40E-52	15175304
		VSAL_I2587	543	Iron(III) ABC transporter (*fbpB*)	*P. haemolytica*	Q9ZHY1	54	7.90E-94	15175304
		VSAL_I2588	337	Iron(III) ABC transporter (*fbpA*)	*P. haemolytica*	Q9Z4N6	62	1.00E-80	15175304
		**VSAL_II1074**	305	Putative iron transporterer, plasmic binding protein	*A. fischeri*	Q5DYV3	82	4.40E-87	No
		**VSAL_II1075**	347	Putative iron transporterer, membrane component	*Y. pestis*	Q56992	38	5.20E-33	10417152
		**VSAL_II1076**	259	Putative iron transporterer, ATP-binding component	*P. aeruginosa*	O68877	37	2.10E-23	8633080
		VSAL_p320_29	354	ABC-type iron ion transport system	*R. palustris*	Q6N7E9	26	6.30E-23	No
	pVS320	*VSAL_p320_30*	347	ABC-type iron ion transport system	*G. diazotrophicus*	Q288C8	38	1.80E-30	No
		VSAL_p320_31	248	ABC-type iron ion transport system	*R. palustris*	Q6N7E7	44	1.60E-21	No
		VSAL_p320_32	429	Hypothetical protein	*R. palustris*	Q6N7E6	28	6.80E-09	No

Miscellaneous

	VSAL_I0498	489	RNA polymerase sigma-54 factor (*rpoN*)	*V. anguillarum*	O08429	74	1.20E-130	9421909
	VSAL_I2058	313	Virulence protein VirC	*V. anguillarum*	Q56567	51	1.00E-52	7590330
	VSAL_I2777	88	RNA-binding protein Hfq	*V. cholerae*	Q9KV11	88	3.30E-28	15225327

[1)]Direction of arrows indicate transcriptional direction of putative operons (Operons predicted based on functional relatedness of genes within the transcriptional unit, intergenic distances and synteny with other species). Vertical typing indicate location on MGE (Table 2 and plasmids)

[2)]Bold gene_IDs indicate a location on chr II, while plasmid gene_IDs are shown in italic

[3)]*A. hydrophila (Areomonas hydrophila), P. aeruginosa (Pseudomonas aeruginosa), E. coli (Escherichia coli), S. frigidimarina (Shewanella frigidimarina), R. meliloti (Rhizobium meliloti), Y. pestis (Yersinia pestis), C. jejuni (Campylobacter jejuni), C. coli (Campylobacter coli), S. boydii (Shigella boydii), S. typhimurium (Salmonella typhimurium), A. punctata (Aeromonas punctata), R. baltica (Rhodopirellula baltica), S. pneumoniae (Streptococcus pneumoniae), P. fluorescens (Pseudomonas fluorescens), S. marcescens (Serratia marcescens), Y. ruckeri (Yersinia ruckeri), A. Actinomycetemcomitans (Actinobacillus actinomycetemcomitans), Y. enterocolitica (Yersinia enterocolitica), R. leguminosarum (Rhizobium leguminosarum), E. agglomerans (Enterobacter agglomerans), S. Hyicus (Staphylococcus hyicus), P. haemolytica (Pasteurella haemolytica), R. palustris (Rhodopseudomonas palustris) and G. diazotrophicus (Gluconacetobacter diazotrophicus)*

*Indicate pseudogenes and gene remnants
