# Supplementary material for: The genome sequence of the fish pathogen Aliivibrio salmonicida strain LFI1238 shows extensive evidence of gene decay
Source: BMC Genomics. 2008 Dec 19;9:616. doi: 10.1186/1471-2164-9-616 (PMC2627896; doi:10.1186/1471-2164-9-616)
Supplement: Additional file 11 — Functional distribution of putative inactivated genes in the A. salmonicida genome. The data provided in this table includes all CDSs disrupted or truncated by IS elements and CDSs containing translational frameshifts of premature stop codons. [file 1471-2164-9-616-S11.pdf]

Additional file 11. Functional distribution of inactivated genes in the *A. salmonicida* genome.

Gene ID	Redundancy*	Product	Type of inactivation
**Unknown function**			
VSAL_I0063		hypothetical protein	Contains a nonsense mutation
VSAL_I0064		hypothetical protein	Truncated by the downstream IS element
VSAL_I0200		hypothetical protein	Disrupted by an IS element
VSAL_I0285		hypothetical protein	Disrupted by an IS element
VSAL_I0482		hypothetical protein MshQ	Contains a frameshift
VSAL_I0776		hypothetical protein	Truncated at the N-terminus
VSAL_I0958		hypothetical protein	Disrupted by an IS element
VSAL_I0964		hypothetical protein	Contains a frameshift
VSAL_I1089		conserved hypothetical protein	Disrupted by an IS element
VSAL_I1111		hypothetical protein	Truncated at the C-terminus
VSAL_I1167		hypothetical protein	Contains a nonsense mutation
VSAL_I1219		hypothetical protein	Truncated by the downstream IS element
VSAL_I1277	Part of VSAL_I1992	hypothetical protein	Truncated by the up- and downstream IS element
VSAL_I1380		hypothetical protein	Truncated by the upstream IS element
VSAL_I1423		hypothetical protein	Contains a nonsense mutation
VSAL_I1458		hypothetical protein	Extended by the downstream IS element
VSAL_I1468		hypothetical protein	Truncated at the N-terminus
VSAL_I1474		hypothetical protein	Truncated at the C-terminus
VSAL_I1496		hypothetical protein	Truncated at the C-terminus
VSAL_I1661		hypothetical protein	Truncated by the upstream IS element
VSAL_I1682		hypothetical protein	Truncated by the downstream IS element
VSAL_I1704		hypothetical protein	Truncated by the upstream IS element
VSAL_I1717		hypothetical protein	Truncated by the upstream IS element and C-term truncation
VSAL_I1719		hypothetical protein	Truncated by the downstream IS element
VSAL_I1742		hypothetical protein	Contains a nonsense mutation
VSAL_I1771		hypothetical protein	Contains a frameshift
VSAL_I1832		hypothetical protein	Truncated by the downstream IS element
VSAL_I1992	Part of VSAL_I1277	hypothetical protein	Truncated by the downstream IS element
VSAL_I2021		conserved hypothetical protein	Contains a frameshift
VSAL_I2052		hypothetical protein	Contains a frameshift
VSAL_I2561		conserved hypothetical protein	Contains a frameshift
VSAL_I2698		hypothetical protein	Contains a frameshift
VSAL_I2957		hypothetical protein	Truncated by the upstream IS element
VSAL_II0004		hypothetical protein	Contains a nonsense mutation
VSAL_II0072		hypothetical protein	Truncated by the upstream IS element and truncated at the C-terminus
VSAL_II0105	Part of VSAL_II0209	hypothetical protein	Truncated by the downstream IS element
VSAL_II0187		hypothetical protein	Truncated by the upstream IS element
VSAL_II0207		hypothetical protein	Extended by the downstream IS element
VSAL_II0209	Part of VSAL_II0105	hypothetical protein	Truncated by the upstream IS element

VSAL_II0358	hypothetical protein	Disrupted by an IS element
VSAL_II0430	hypothetical protein	Contains a nonsense mutation
VSAL_II0647	hypothetical protein	Contains a frameshift
VSAL_II0746	conserved hypothetical protein	Disrupted by an IS element
VSAL_II0874	hypothetical protein	Truncated by the upstream IS element
VSAL_II0924	hypothetical protein	Contains a nonsense mutation, a frameshift and truncated by downstream IS element
VSAL_II1000	hypothetical protein	Contains a nonsense mutation
VSAL_II1015	hypothetical protein	Extended by the downstream IS element
VSAL_II1058	hypothetical protein	Truncated by the upstream IS element
VSAL_II1085	hypothetical protein	Truncated by the upstream IS element
VSAL_II1089	hypothetical protein	Truncated by the downstream IS element
VSAL_II1100	hypothetical protein	Contains a nonsense mutation

51 genes
30 affected by IS elements
21 mutations or truncations

Chemotaxis and mobility

VSAL_I1444	methyl-accepting chemotaxis protein	Contains a frameshift and is disrupted by an IS element
VSAL_I1462	methyl-accepting chemotaxis protein	Contains a frameshift and is disrupted by an IS element
VSAL_I1707	putative methyl-accepting chemotaxis protein	Disrupted by an IS element and truncated at the C-terminus
VSAL_I1812	putative methyl-accepting chemotaxis protein	Truncated by the downstream IS element
VSAL_I1822	methyl-accepting chemotaxis protein	Truncated by the upstream IS element
VSAL_I2117	methyl-accepting chemotaxis protein	Extended by the downstream IS element
VSAL_I2308	polar flagellar M-ring protein FliF	Contains a nonsense mutation
VSAL_I2316	polar flagellar protein FlaG	Contains a nonsense mutation
VSAL_I2601	methyl-accepting chemotaxis protein	Contains a frameshift
VSAL_I3036	methyl-accepting chemotaxis protein	Truncated by the downstream IS element
VSAL_II0282	methyl-accepting chemotaxis protein	Contains a nonsense mutation
VSAL_II0443	methyl-accepting chemotaxis protein	Contains a frameshift
VSAL_II0769	methyl-accepting chemotaxis protein	Truncated by the downstream IS element

13 genes
8 affected by IS elements
5 mutations or truncations

Transport/binding proteins

VSAL_I0071	Trk system potassium uptake protein	Extended by the downstream IS element
VSAL_I0094	ABC transporter, ATP-binding protein	Contains a frameshift
VSAL_I0202	membrane permease	Contains a frameshift
VSAL_I0657	ABC transporter protein, ATP-binding component	Contains a frameshift
VSAL_I0743	inner membrane transport protein	Disrupted by an IS element
VSAL_I0867	oligopeptide transport ATP-binding protein	Contains a nonsense mutation
VSAL_I0894	sodium/solute symporter	Truncated by the upstream IS element
VSAL_I1148	sodium/dicarboxylate symporter	Contains a frameshift

VSAL_I1165		putative sodium/sulfate symporter	Truncated by the downstream IS element
VSAL_I1261		PTS system EIIB, membrane component	Contains frameshifts and a 200 amino acid deletion
VSAL_I1338		tyrosine-specific transport protein	Extended by the downstream IS element
VSAL_I1409		transporter, BCCT family	Disrupted by an IS element
VSAL_I1631		sodium/solute symporter	Truncated by the downstream IS element
VSAL_I1657		PTS system, EIIC membrane component	Contains a frameshift
VSAL_I1751		TonB protein	Contains a frameshift
VSAL_I1794		Na(+) driven multidrug efflux pump	Truncated at the N-terminus
VSAL_I1947		oligopeptide transport system permease protein B	Contains a nonsense mutation
VSAL_I2008	Part of VSAL_I2045	putative long-chain fatty acid transport protein	Truncated by the downstream IS element
VSAL_I2013		ABC transporter, ATP binding protein	Contains a frameshift
VSAL_I2014		putative ABC transporter, ATP-binding protein	Truncated by the upstream IS element
VSAL_I2016		secretion protein, HlyD family	Truncated by the upstream IS element
VSAL_I2045	Part of VSAL_I2008	putative long-chain fatty acid transport protein	Truncated by the downstream IS element
VSAL_I2069		putative ABC transporter, ATP-binding protein	Truncated by the downstream IS element
VSAL_I2160		Na+/H+ antiporter	Contains a frameshift and truncated by the downstream IS element
VSAL_I2352		chitoporin	Contains a frameshift
VSAL_I2459		putative sodium/solute symporter	Contains a frameshift and disrupted by an IS element
VSAL_I2576		peptide ABC transporter, periplasmic peptide-binding protein	Contains a frameshift and a 8 amino acid deletion
VSAL_I2810		anaerobic C4-dicarboxylate transporter DcuA	Contains a frameshift
VSAL_II0094		membrane transport protein, LysE type	Extended by the downstream IS element
VSAL_II0133	Part of VSAL_II1051	putative sodium/sulfate symporter	Truncated by the downstream IS element
VSAL_II0157		ABC transporter, ATP-binding component	Disrupted by an IS element
VSAL_II0199		putative potassium channel	Contains a frameshift
VSAL_II0233		maltose/maltodextrin transport ATP-binding protein	Truncated by the upstream IS element
VSAL_II0828		secretion protein, HlyD family	Disrupted by an IS element
VSAL_II0841		Na+/H+ antiporter NhaC	Contains a frameshift and a 280 amino acid deletion
VSAL_II0898		putative PTS system, IIC component	Contains a frameshift
VSAL_II0907		iron(III) ABC transporter, periplasmic iron-compound-binding protein	Contains a frameshift
VSAL_II0919	Part of VSAL_II0927	maltose transport system permease protein MalF	Truncated by the upstream IS element
VSAL_II0927	Part of VSAL_II0919	maltose transport system permease protein MalF	Truncated by the upstream and downstream IS elements
VSAL_II1051	Part of VSAL_II0133	putative sodium/sulfate symporter	Truncated by the upstream IS element
VSAL_II1078		divalent cation transport protein	Contains a nonsense mutation
VSAL_II1087		MFS transporter	Truncated by the upstream IS element

42 genes
22 affected by IS elements
20 mutations or truncations

Protection responses

VSAL_I1336		multidrug efflux pump	Truncated at the C-terminus
VSAL_I1734		heme receptor	Contains a frameshift
VSAL_I1910		penicillin-binding protein 2 (PBP-2)	Disrupted by an IS element
VSAL_I1964		putative multidrug resistance protein	Contains a frameshift
VSAL_I2906		integral membrane protein, MarC family	Contains a frameshift

VSAL_I3052		multidrug efflux pump	Disrupted by an IS element
VSAL_II0016		putative plasmid-encoded multidrug efflux pump	Truncated by the downstream IS element
VSAL_II0029		transporter, acriflavin resistance protein	Disrupted by an IS element
VSAL_II0709		multidrug efflux pump	Contains a frameshift
VSAL_II0955		putative multidrug transport protein	Contains a frameshift

10 genes
4 affected by IS elements
6 mutations or truncations

Adaptation

VSAL_I1317		carbon starvation protein	Contains a nonsense mutation

1 gene
1 mutation

Macromolecule degradation

VSAL_I0763	Part of VSAL_I0902	chitinase A	Truncated by the upstream IS element
VSAL_I0819		ATP-dependent Clp protease ATP-binding subunit ClpX	Disrupted by an IS element
VSAL_I0902	Part of VSAL_I0763	chitinase A	Truncated by the downstream IS element
VSAL_I1108		chitodextrinase	Truncated by the downstream IS element and N-term truncation
VSAL_I1238		exported serine protease	Disrupted by an IS element
VSAL_I1414		putative chitinase	Disrupted by an IS element
VSAL_I1429		putative peptidase	Disrupted by an IS element
VSAL_I1493		microbial collagenase precursor	Contains a nonsense mutation
VSAL_I1546		aminopeptidase N	Contains a nonsense mutation
VSAL_I1942		chitinase	Disrupted by an IS element
VSAL_I2164		peptidase family M16	Extended by the downstream IS element
VSAL_I2483		putative type I restriction enzyme R protein	Truncated by the downstream IS element
VSAL_II0003		proline iminopeptidase	Contains a frameshift

13 genes
10 affected by IS elements
3 mutations or truncations

Macromolecule synthesis, modification

VSAL_I0166		putative acylneuraminate cytidylyltransferase	Contains a nonsense mutation
VSAL_I0175		lipopolysaccharide biosynthesis protein WavS	Contains a nonsense mutation
VSAL_I0178		putative dTDP-4-dehydrorhamnose reductase	Contains two nonsense mutations
VSAL_I0213		tRNA (guanosine-2'-o-)-methyltransferase	Truncated by the downstream IS element
VSAL_I0245		putative dTDP-4-dehydrorhamnose reductase	Contains a nonsense mutation
VSAL_I0248		lipopolysaccharide biosynthesis protein WavS	Contains a nonsense mutation
VSAL_I0257		putative acylneuraminate cytidylyltransferase	Contains a nonsense mutation
VSAL_I0460		colanic biosynthesis UDP-glucose lipid carrier transferase	Contains a framshift

VSAL_I0565		peptide chain release factor 2 (RF-2)	Contains a framshift
VSAL_I0970		DNA polymerase IV	Truncated at the N-terminus
VSAL_I1275	Part of VSAL_I1994	helicase	Truncated by the downstream IS element
VSAL_I1479		putative acyltransferase	Truncated by the upstream IS element
VSAL_I1679		type I restriction enzyme R protein	Contains a nonsense mutation
VSAL_I1691		tRNA-(ms[2]io[6]A)-hydroxylase	Disrupted by an IS element
VSAL_I1791		type I restriction-modification system specificity subunit	Contains a nonsense mutation
VSAL_I1994	Part of VSAL_I1275	helicase	Truncated by the upstream IS element
VSAL_I2606		glutamyl-Q tRNA(Asp) synthetase	Contains a frameshift
VSAL_I2852		NADH pyrophosphatase	Disrupted by an IS element
VSAL_II0040		1,4-alpha-glucan branching enzyme	Extended by the downstream IS element
VSAL_II0412		putative DNA methylase	Contains a nonsense mutation and truncated at the N-terminus

20 genes
7 affected by IS elements
13 mutations or truncations

Amino acid biosynthesis

VSAL_I0041	Part of VSAL_I0055	acetolactate synthase isozyme II large subunit	Truncated by the downstream IS element
VSAL_I0055	Part of VSAL_I0041	acetolactate synthase isozyme II large subunit	Truncated by the downstream IS element
VSAL_I0443		lysine-sensitive aspartokinase III	Disrupted by an IS element
VSAL_I0861	Part of VSAL_I0916	5-methyltetrahydropteroyltriglutamate--homocysteine methyltransferase	Truncated by the downstream IS element
VSAL_I0916	Part of VSAL_I0861	5-methyltetrahydropteroyltriglutamate--homocysteine methyltransferase	Truncated by the downstream IS element

5 genes
5 affected by IS elements

Biosynthesis of cofactors, carriers

VSAL_I0156	Part of VSAL_I0237	phosphopantetheine adenylyltransferase	Truncated by the upstream IS element
VSAL_I0237	Part of VSAL_I0156	phosphopantetheine adenylyltransferase	Truncated by the downstream IS element
VSAL_I0513		isopentenyl-diphosphate delta-isomerase	Disrupted by an IS element
VSAL_II0273		siderophore biosynthesis protein IucA	Truncated by the upstream IS element
VSAL_II0275		siderophore biosynthesis protein IucC	Disrupted by an IS element

5 genes
5 affected by IS elements

Central intermediary metabolism

VSAL_I0145		guanosine-5'-triphosphate,3'-diphosphate pyrophosphatase	Contains a frameshift
VSAL_I0622		purine nucleoside phosphorylase	Contains a nonsense mutation
VSAL_I1548		NAD-dependent glutamate dehydrogenase	Contains a frameshift
VSAL_II0255		agmatinase	Contains a frameshift and disrupted by an IS element
VSAL_II0830		putative aldo-keto reductase	Extended by the downstream IS element

5 genes
2 affected by IS elements
3 mutations or truncations

Degradation of small molecules

VSAL_I0906		long-chain-fatty-acid-CoA ligase	Truncated by the downstream IS element
VSAL_I1521		putative arginase	Truncated by the downstream IS element

2 genes
2 affected by IS elements

Energy metabolism, carbon

VSAL_I2366		periplasmic nitrate reductase precursor NapA	Contains a frameshift
VSAL_I2624		dihydrolipoyllysine-residue acetyltransferase	Truncated at the N-terminus
VSAL_II0005		cytochrome b561 CybB	Truncated by the upstream IS element
VSAL_II0108	Part of VSAL_II0193	biotin sulfoxide reductase	Truncated by the downstream IS element
VSAL_II0109		cytochrome C-type protein	Contains a frameshift
VSAL_II0162		cytochrome o ubiquinol oxidase subunit I	Contains a frameshift
VSAL_II0193	Part of VSAL_II0108	biotin sulfoxide reductase	Truncated by the upstream IS element
VSAL_II0514		major NAD(P)H-flavin oxidoreductase	Disrupted by an IS element

8 genes
4 affected by IS elements
4 mutations or truncations

Fatty acid and phosphatidic acid biosynthesis

VSAL_I2089		3-oxoacyl-[acyl-carrier-protein] synthase 2	Extended by the downstream IS element
VSAL_II0872		cyclopropane-fatty-acyl-phospholipid synthase	Contains a frameshift

2 genes
1 affected by IS elements
1 mutations or truncations

Membrane/exported/lipoproteins

VSAL_I0036		toxin coregulated pilus biosynthesis protein I	Truncated by the upstream IS element
VSAL_I0037		putative exported protein	Truncated by the downstream IS element
VSAL_I0455		putative chain length determinant protein	Disrupted by an IS element
VSAL_I0472		type IV pilus, mannose-sensitive hemagglutinin E	Contains a nonsense mutation
VSAL_I0943		outer membrane protein OmpK	Contains a nonsense mutation
VSAL_I0971		alanine racemase	Disrupted by an IS element
VSAL_I1104	part of VSAL_I1002	membrane protein	Truncated by the downstream IS element
VSAL_I1002	part of VSAL_I1104	membrane protein	Truncated by the downstream IS element
VSAL_I1113		putative type VI secretion protein VasK	Contains a nonsense mutation
VSAL_I1130		putative outer membrane protein	Contains a nonsense mutation
VSAL_I1183		putative type VI secretion protein VasK-1	Disrupted by an IS element

VSAL_I1208		putative exported protein	Extended by the downstream IS element
VSAL_I1265		putative exported protein	Truncated by the downstream IS element
VSAL_I1290		outer membrane protein, OmpA family	Disrupted by an IS element
VSAL_I1480		putative membrane protein	Truncated at the N-terminus
VSAL_I1523	Part of VSAL_I1602	putative membrane protein	Truncated by the upstream IS element
VSAL_I1525		putative lipoprotein	Contains a frameshift and truncated at the N-terminus
VSAL_I1602	Part of VSAL_I1523	putative membrane protein	Truncated by the downstream IS element
VSAL_I1604		putative exported protein	Truncated by the downstream IS element
VSAL_I1702		putative lipoprotein	Extended by the downstream IS element
VSAL_I1722		membrane associated GGDEF protein	Disrupted by an IS element
VSAL_I1735		membrane protein	Truncated at the C-terminus
VSAL_I1738		putative membrane protein	Truncated by the downstream IS element
VSAL_I1744		VgrG protein, VgrG-1	Disrupted by an IS element
VSAL_I1923		putative outer membrane protein OmpA	Contains a frameshift
VSAL_I2012		putative membrane protein	Truncated by the downstream IS element
VSAL_I2177		putative exported protein	Disrupted by an IS element
VSAL_I2403		putative membrane protein	Disrupted by an IS element
VSAL_I2447		putative exported protein	Extended by the downstream IS element
VSAL_I2468		putative lipoprotein	Disrupted by an IS element
VSAL_I2596		putative exported protein	Extended by the downstream IS element
VSAL_I2631		type IV pilus assembly protein PilC	Contains a frameshift
VSAL_I2720		fimbrial assembly protein PilQ precursor	Disrupted by an IS element
VSAL_I2855		curli production assembly/transport component, CsgG precursor	Contains a frameshift
VSAL_I2859		minor curlin subunit, CsgB like	Disrupted by an IS element
VSAL_I3022		putative membrane protein	Truncated by the downstream IS element
VSAL_II0049		putative membrane protein	Truncated by the upstream IS element
VSAL_II0336		membrane protein	Extended by the downstream IS element
VSAL_II0379		outer membrane protein, OmpA family	Contains a nonsense mutation
VSAL_II0388		putative secreted hydrolase	Disrupted by an IS element
VSAL_II0466		putative membrane protein	Truncated by the downstream IS element
VSAL_II0494		putative membrane protein	Truncated by the downstream IS element
VSAL_II0617		outer membrane efflux protein	Contains a frameshift
VSAL_II0679		putative membrane protein	Contains a nonsense mutation
VSAL_II0877		membrane protein	Truncated by the downstream IS element
VSAL_II0931		membrane protein	Truncated by the downstream IS element
VSAL_II0972		putative membrane protein	Truncated by the downstream IS element
VSAL_II0804		putative lipoprotein	Extended by the downstream IS element
VSAL_II0899		putative exported protein	Disrupted by an IS element
VSAL_II0989		putative exported protein	Truncated by the downstream IS element
VSAL_II1008		putative type IV pilus biogenesis protein	Truncated by the upstream IS element
VSAL_II1096		membrane protein	Truncated by the upstream IS element

51 genes
40 affected by IS elements
13 mutations or truncations

Transposon-related functions

VSAL_I0013	transposase	Disrupted by an IS element
VSAL_I0068	transposase	Contains a frameshift
VSAL_I0130	transposase	Truncated by the downstream IS element
VSAL_I0153	transposase	Contains a frameshift
VSAL_I0154	transposase	Contains a frameshift
VSAL_I0281	transposase	Contains a frameshift
VSAL_I0314	transposase	Disrupted by an IS element
VSAL_I0318	transposase	Contains a frameshift
VSAL_I0357	transposase	Contains a frameshift
VSAL_I0438	transposase	Truncated by the upstream IS element
VSAL_I0514	transposase	Contains several frameshifts
VSAL_I0764	transposase	Contains several frameshifts
VSAL_I0765	transposase	Contains a nonsense mutation
VSAL_I0808	transposase	Disrupted by an IS element
VSAL_I0897	transposase	Contains a frameshift
VSAL_I0905	transposase	Contains two nonsense mutations
VSAL_I1000	transposase	Disrupted by an IS element
VSAL_I1063	transposase	Contains a frameshift and a possible truncation
VSAL_I1106	transposase	Contains a frameshift
VSAL_I1107	transposase	Contains a frameshift
VSAL_I1241	transposase	Contains a frameshift
VSAL_I1258	transposase	Contains a frameshift
VSAL_I1267	transposase	Contains a frameshift
VSAL_I1268	transposase	Contains a frameshift
VSAL_I1315	transposase	Contains a frameshift
VSAL_I1316	transposase	Contains a frameshift
VSAL_I1361	transposase	Contains a frameshift
VSAL_I1440	transposase	Disrupted by an IS element
VSAL_I1653	transposase	Truncated by the insertion of the upstream and downstream IS element
VSAL_I1716	transposase	Contains a frameshift
VSAL_I1778	transposase	Contains a nonsense mutation
VSAL_I1811	transposase	Contains a frameshift
VSAL_I1821	transposase	Truncated at the N-terminus
VSAL_I1833	transposase	Contains several frameshifts
VSAL_I1835	transposase	Contains a frameshift
VSAL_I1945	transposase	Contains a nonsense mutation
VSAL_I2009	transposase	Contains a frameshift
VSAL_I2086	transposase	Contains two nonsense mutations
VSAL_I2265	transposase	Contains a frameshift
VSAL_I2320	transposase	Contains a frameshift
VSAL_I2321	transposase	Truncated by the insertion of the upstream IS element
VSAL_I2322	transposase	Disruptet by the insertion of an IS element
VSAL_I2462	transposase	Contains a frameshift

VSAL_I2487	transposase	Contains a frameshift
VSAL_I2612	transposase	Contains a frameshift
VSAL_I2620	transposase	Contains a frameshift
VSAL_I2621	transposase	Contains a frameshift
VSAL_I2809	transposase	Contains a frameshift
VSAL_I2862	transposase	Truncated by the insertion of the upstream IS element
VSAL_I2933	transposase	Contains a frameshift
VSAL_I2958	transposase	Contains a frameshift
VSAL_I3007	transposase	Contains a frameshift
VSAL_I3017	transposase	Contains a frameshift
VSAL_I3023	transposase	Contains a frameshift
VSAL_I3034	transposase	Truncated by the upstream IS element
VSAL_I3037	transposase	Contains a frameshift and two nonsense mutations
VSAL_I3042	transposase	Contains a frameshift
VSAL_I3053	transposase	Contains a frameshift
VSAL_II0007	transposase	Disrupted by an IS element
VSAL_II0009	transposase	Contains a nonsense mutation
VSAL_II0022	transposase	Truncated by the insertion of a downstream IS element
VSAL_II0025	transposase	Contains a frameshift
VSAL_II0030	transposase	Disrupted by an IS element
VSAL_II0044	transposase	Truncated by the downstream IS element
VSAL_II0045	transposase	Truncated by the downstream IS element
VSAL_II0071	transposase	Contains a frameshift
VSAL_II0082	transposase	Contains a nonsense mutation
VSAL_II0129	transposase	Disrupted by an IS element
VSAL_II0189	transposase	Truncated by the downstream IS element
VSAL_II0227	transposase	Contains a nonsense mutation
VSAL_II0257	transposase	Contains a frameshift
VSAL_II0272	transposase	Contains a frameshift
VSAL_II0535	transposase	Contains a nonsense mutation
VSAL_II0563	transposase	Truncated by the upstream IS element
VSAL_II0698	transposase	Contains a frameshift
VSAL_II0700	transposase	Contains a frameshift
VSAL_II0768	transposase	Contains a nonsense mutation
VSAL_II0831	transposase	Contains a frameshift
VSAL_II0976	transposase	Truncated by the upstream IS element
VSAL_II0992	transposase	Disrupted by an IS element
VSAL_II1005	transposase	Contains a frameshift
VSAL_II1049	transposase	Contains a frameshift
VSAL_II1092	transposase	Contains a frameshift

83 genes
22 affected by IS elements
61 mutations or truncations

Phage-related functions and prophages

VSAL_I0395		hypothetical protein	Truncated by the downstream IS element
VSAL_I0767		hypothetical protein, putative phage integrase	Truncated by the downstream IS element
VSAL_I0774		putative portal vertex protein	Contains a frameshift
VSAL_I0776		hypothetical protein	Truncated at the N-terminus
VSAL_I1033		hypothetical protein, putative phage gene	Contains a frameshift
VSAL_I1926		immunoglobulin-binding regulator	Contains a frameshift and a 24 amino acid deletion
VSAL_I1927		hypothetical protein	Truncated by the upstream IS element
VSAL_I2122		putative capsid portal protein	Truncated at the N-terminus
VSAL_I2123		putative bacteriocin immunity protein	Truncated at the N-terminus
VSAL_I2263		hypothetical protein, putative phage integrase	Truncated by the upstream and downstream IS elements
VSAL_II0410		hypothetical protein, putative phage gene	Truncated at the N-terminus
VSAL_II0502		putative bacteriophage integrase	Truncated at the C-terminus
VSAL_II0629		hypothetical protein	Truncated by the insertion of the upstream bacteriophage
VSAL_II0632		hypothetical protein, putative phage integrase	Truncated at the N-terminus
VSAL_II0635		hypothetical protein, putative phage integrase	Truncated at the N-terminus

15 genes
4 affected by IS elements
11 mutations or truncations

Pathogenicity island-related functions

VSAL_I0067		putative hemolysin/hemagglutinin-like protein	Truncated by the upstream and downstream IS elements
VSAL_I0863	Part of VSAL_I0918	accessory colonization factor AcfD precursor	Truncated by the downstream IS element
VSAL_I0918	Part of VSAL_I0863	accessory colonization factor AcfD precursor	Truncated by the upstream IS element
VSAL_II0528		hemolysin secretion protein	Contains a frameshift

4 genes
3 affected by IS elements
1 mutation

Regulation

VSAL_I0378	transcriptional regulator, AcrR/TetR family	Contains a nonsense mutation
VSAL_I0673	HTH-type transcriptional regulator, LysR family	Contains a nonsense mutation
VSAL_I0813	transcriptional regulator ToxR protein	Contains a frameshift
VSAL_I1118	transcriptional regulatory protein, Fis family VasH	Contains a frameshift
VSAL_I1402	putative sigma-54 dependent response regulator	Contains a frameshift
VSAL_I1434	HTH-type transcriptional regulator, LysR family	Contains a nonsense mutation
VSAL_I2185	histidine kinase	Contains a nonsense mutation
VSAL_I2256	putative regulatory protein, AraC family	Contains a frameshift
VSAL_II0186	HTH-type transcriptional regulator, LysR family	Contains a nonsense mutation
VSAL_II0195	putative response regulator	Contains a frameshift
VSAL_II0612	HTH-type transcriptional regulator, LysR family	Contains a frameshift
VSAL_II0642	putative membrane associated signal transducer	Disrupted by an IS element
VSAL_II0724	membrane associated histidine kinase	Contains a frameshift

VSAL_II0943	autoinducer 1 sensor kinase/phosphatase LuxN	Contains a frameshift
VSAL_II0945	HTH-type transcriptional regulator, GntR-family	Contains a frameshift
VSAL_II1004	HTH-type transcriptional regulator, LacI family	Truncated by the downstream IS element
VSAL_II1039	HTH-type transcriptional regulator, LysR family	Contains a nonsense mutation
VSAL_II1047	putative transcriptional regulator, LysR family	Truncated by the upstream IS element
VSAL_II1069	HTH-type transcriptional regulator, LysR family	Contains a frameshift
		19 genes<br>3 affected by IS elements<br>16 mutations or truncations

Others

VSAL_I0929	autoinducer 2-binding periplasmic protein LuxP precursor	Contains a frameshift
		1 gene<br>1 mutations or truncations

Not classified (included putative assignments)

VSAL_I1360	hypothetical protein, putative cell wall degradation protein	Extended by the downstream IS element
VSAL_I1461	putative signaling protein	Contains a nonsense mutation and truncated at C-terminus
VSAL_I1629	glycosyl transferase, family 2	Contains a frameshift
VSAL_I1710	putative hemolysin	Contains frameshift and nonsense mutation
VSAL_I1818	phosphoesterase	Contains a frameshift
VSAL_I2036	putative genetic competence protein	Truncated by the downstream IS element
VSAL_I2950	putative signaling protein	Disrupted by an IS element
VSAL_II0244	molybdopterin oxidoreductase	Contains a frameshift and truncated by the downstream IS element
VSAL_II0332	putative hemolysin-type calcium-binding protein	Truncated by the downstream IS element
VSAL_II0347	putative arylsulfatase-activating protein	Contains a frameshift
VSAL_II0453	putative HTH-type transcriptional regulator	Disrupted by an IS element
VSAL_II0655	putative phosphate-binding protein	Contains a frameshift
VSAL_II0761	putative signaling protein	Truncated by the upstream IS element
VSAL_II0801	putative RHS protein	Truncated by the upstream IS element
VSAL_II0971	putative cytotoxic necrotizing factor 1	Truncated by the upstream IS element
VSAL_II1063	putative HTH-type transcriptional regulator	Truncated by the upstream IS element
		16 genes<br>10 affected by IS elements<br>6 mutations or truncations

**Total in the chromosomal DNA**	**367 genes**<br>**181 affected by IS elements**<br>**186 mutations or truncations**

Plasmids

VSAL_p320_17	putative exported protein	Truncated by the downstream IS element
VSAL_p320_25	putative MobA protein	Truncated by the down- and upstream IS element
VSAL_p320_32	transposase	Contains a nonsense mutation

**Total in the genome**	**370 genes**<br>**183 disrupted (55) or truncated (115) or extended (13) by IS elements**<br>**187 mutations (164) or truncations (23)**

*Gene ID of the appurtenant part of CDS. Not merged due to distant localisation on the chromosome.
